# Functional diversity of human protein kinase splice variants marks significant expansion of human kinome

**DOI:** 10.1186/1471-2164-10-622

**Published:** 2009-12-22

**Authors:** Krishanpal Anamika, Nicolas Garnier, Narayanaswamy Srinivasan

**Affiliations:** 1 Molecular Biophysics Unit, Indian Institute of Science, Bangalore 560 012, India; 2 Current address: Department of Functional Genomics, Institute for Genetics and Molecular and Cellular Biology, 1 rue Laurent Fries/BP 10142/67404 Illkirch Cedex Strasbourg, France

## Abstract

**Background:**

Protein kinases are involved in diverse spectrum of cellular processes. Availability of draft version of the human genomic data in the year 2001 enabled recognition of repertoire of protein kinases. However, over the years the human genomic data is being refined and the current release of human genomic data has helped us to recognize a larger repertoire of over 900 human protein kinases represented mainly by splice variants.

**Results:**

Many of these identified protein kinases are alternatively spliced products. Interestingly, some of the human kinase splice variants appear to be significantly diverged in terms of their functional properties as represented by incorporation or absence of one or more domains. Many sets of protein kinase splice variants have substantially different domain organization and in a few sets of splice variants kinase domains belong to different subfamilies of kinases suggesting potential participation in different signal transduction pathways.

**Conclusions:**

Addition or deletion of a domain between splice variants of multi-domain kinases appears to be a means of generating differences in the functional features of otherwise similar kinases. It is intriguing that marked sequence diversity within the catalytic regions of some of the splice variant kinases result in kinases belonging to different subfamilies. These human kinase splice variants with different functions might contribute to diversity of eukaryotic cellular signaling.

## Background

Protein kinases represent one of the largest protein domain families in most of the higher eukaryotes. Members of protein kinase family are involved in innumerable signal transduction pathways contributing in the decision making on variety of cellular processes such as cell growth, development, differentiation, metabolism, cell communication and apoptosis. Availability of the first draft version of the human genomic data in the year 2001 [1] provided opportunity to recognize the repertoire of protein kinases in the human [2-4]. These studies employed bioinformatics approaches to recognize kinases starting from the genomic data and provided a classification of kinases that give hint to the signal transduction pathways in which the kinases participate. Due to differences in the human genome data set employed and also due to different bioinformatics approaches employed by different groups the number of protein kinases reported in these early publications differed slightly though a vast majority of kinases forming a common set [5].

Quality of the human genomic data is being continuously improved since its first release in 2001 [1]. In the early draft dataset the order of the base pairs in each chromosomal area has been determined 4 or 5 times (4× or 5×) or even more [1]. However in April 2003 human genome data of better quality was compiled in which additional sequencing improved the accuracy significantly http://www.ornl.gov/TechResources/Human_Genome/project/50yr.html. With the improvements in genome sequencing projects, order of the base pairs in each chromosomal area has been determined 8 or 9 times (8× or 9×) and the quality of the human genomic data improved significantly over the time. The present version of the human genomic data and the annotation are of superior quality compared to the first release about 8 years ago. This accurately annotated genomic sequence data set has impact in the quality of other kinds of genome-wide data sets such as, transcript sequences, expression data, disease influenced gene regulation and functional information.

Alternative splicing is an important mechanism to increase proteomic diversity [6] which produces diverse transcripts and hence potentially diverse protein products from the same gene locus which eventually add significant complexity to the genome. In typical alternative splicing process different combinations of exons within a gene are spliced from RNA precursor and reassembled in the mature mRNA. The nature of splice variant could be further related to the tissue type, developmental stage and disease versus normal conditions of the cell. Thus splicing could result in multitude of proteins originating from single gene and these protein products often have interesting diversity in functional properties. For example Drosophila *Dscam *gene, an axon guidance receptor gene, is a striking example which produces more than a thousand gene products [7]. Alternative splicing mechanism has been studied for various mammalian systems as well as for plants [[8-11]; http://www.itb.cnr.it/kinweb]. Analysis of some of the human kinase splice variants has been performed in the past [[11]; http://www.itb.cnr.it/kinweb] which also gives information about their domain combination. Though human and mouse are very close mammalian species, it has been observed that the conservation of alternative splicing pattern is low [12-14] which might be the reason for the generation of species-specific gene products. Though the human kinome analysis performed in 2001 [2-4] identified over 500 kinases, it was earlier anticipated that the number of human protein kinases might be around 1000 [15]. We suggest that higher estimate still holds good as the splicing mechanism in human is very common. Bioinformatics analysis indicates that approximately 40-60% of the human genes are known to alternatively spliced [16-19] with majority of splicing events occurring in 5' untranslated regions. Splicing event allows production of more than one protein isoform from a single gene which may have altered substrate affinities, sub-cellular localizations, and may exhibit different and sometimes antagonistic functional and structural properties [20-22]. In fact, various protein kinase splice variants have been experimentally characterized and they are shown to elicit different expression patterns, differential localization and hence different functional properties [23-26]. It is known from a previous study that alternative transcripts of protein kinases encode different domain structures and these variants are likely to play important roles in phosphorylation-dependent signaling pathways [8]. Splice variants of human centrosome kinase Nek2 are known to exhibit different pattern of expression in mitosis [24]. Two splice forms of human protein kinase B are shown to have distinct regulatory capacity depending upon presence or absence of phosphorylation site in the carboxy terminal hydrophobic domain [27]. Two splice forms of Mitogen-activated protein (MAP) kinase-interacting kinase (Mnk) derived from same gene differ only at their C termini. It has been seen that while Mnk2a contains a MAP kinase-binding site in this region, Mnk2b lacks such a sequence and is much less readily activated by MAP kinases *in vitro*. Mnk2a is cytoplasmic whereas substantial amount of Mnk2b is found in the nucleus [25]. Splice variants of Serum and Glucocorticoid-Inducible Kinase 1 (SGK1) which belongs to AGC group of protein kinase known to be regulated differentially following differentiation and shown to be markedly upregulated in tumor tissues [28]. TrkC, a receptor tyrosine kinase has a number of naturally occurring splice variants including one with a 14 residues insertion between subdomain VII and VIII in the C-terminal lobe of the kinase domain. This insert in the catalytic domain of TrkC results in defective MAPK activation which may result predominantly from an inhibition of high-affinity Shc binding [29]. Splicing mechanism is very carefully regulated as aberrant regulation of alternative splicing in human has been implicated in various diseases [30,31].

Most of the analyses on identification and analysis of comprehensive collection of human gene splice products have been performed before or around the time when the first draft of the human genome sequence data was released. Thus, these analyses were performed using incomplete set of human gene products. Moreover, despite availability of complete human genomic data in the subsequent years, the improvement in the quality of the genomic data has been gradual which has prompted us to identify and analyse repertoire of protein kinase gene products with the present information on splice variants of human kinases. Our objective in this analysis is to extend our understanding of repertoire of functions of human protein kinases and extent to which the functional diversity could occur among splice variants of human kinases.

## Results and discussion

### Identification and analysis of a comprehensive collection of human protein kinases

Currently, there are two online sites that constantly update the information on repertoire of human protein kinases gleaned periodically from the releases of human genome data. These data sets KinG [Kinases in Genome http://hodgkin.mbu.iisc.ernet.in/~king] [32] and kinase.com http://kinase.com/kinbase/FastaFiles/ are quite similar in terms of list of human kinases and their classification into subfamilies. Both these datasets of human kinome are obtained as a result of careful use of highly sophisticated sequence analysis tools. For example, in the recent releases of KinG database http://hodgkin.mbu.iisc.ernet.in/~king, we have employed highly sensitive multiple profile search approach [33,34]. During this update of the database and analysis many protein kinases have come into light which are splice variants of previously identified protein kinases listed in various kinome datasets such as the one in http://www.itb.cnr.it/kinweb[11]. This prompted us to extend and analyse the repertoire of protein kinase gene products by including protein kinase splice variants in the dataset. The improved human genome data enriched with information on splice variants available in ENSEMBL database http://www.ensembl.org/Homo_sapiens/index.html has helped us to analyze protein kinase splice variants which were not studied in the previous genome-wide studies on kinases using the draft version of human genomic data [2-4].

In the current analysis, using various sensitive profile search methods (discussed in Materials and Methods section), we report 918 putative human protein kinase gene products (Additional file 1). In addition this list integrated with user-friendly search options is also available as a part of KinG database at http://hodgkin.mbu.iisc.ernet.in/king/cgi/search. All these 918 kinase gene products have catalytic aspartate, which acts as catalytic base, suggesting that these gene products are likely to possess phosphotransferase activity. Sequences lacking the catalytic aspartate (protein kinase-like non-kinases) have not been included in the current list. There are 445 genes which are encoding 918 protein kinase products. There are 209 genes which are encoding only one protein kinase. Remaining 236 kinase genes give rise to more than one gene product which are splice variants. Maximum number of splice variants generated from a protein kinase gene is 12.

We checked for the expression of the human protein kinase splice variants using the publicly available cDNA data ftp://ftp.ensembl.org/pub/current_fasta/homo_sapiens/cdna/ and found that 894 out of 918 have known cDNA data. Hence overwhelming majority of the recognized protein kinase genes is probably expressed and represents functional proteins. We have compared our kinome data set to kinase.com http://www.kinase.com in which so far 516 protein kinase gene products have been listed of which 25 are atypical protein kinases which are different from typical eukaryotic protein kinase superfamily discussed in the current analysis.

### Classification of protein kinase gene products

These putative protein kinases have been further classified into various subfamilies as proposed by Hanks and Hunter [35]. The most represented protein subfamily in human is CAMK (Ca^2+^/Calmodulin dependent Protein Kinase) (Additional file 1 and http://hodgkin.mbu.iisc.ernet.in/king/cgi/search) group of protein kinases regulated by Calcium/Calmodulin having 161 members which is followed by CMGC (CDK, MAPK, GSK3, CK2) group of protein kinases which have 149 members. There are 130 protein products belonging to AGC group of kinases which are mainly second messenger regulated kinases. Several members which are closely related to protein tyrosine kinase family and Tyrosine kinase-like (TKL) subfamily have also been identified. TKL family is quite diverse which have members (e.g. Raf, Mixed-lineage kinase, Activin and beta-receptor kinase, Interleukin 1 receptor associated kinase etc.) resembling both tyrosine and Serine/Threonine kinase families.

### Human kinases splice variants with potentially diverse functions: revealed from sequence analysis

**Figure 1:**
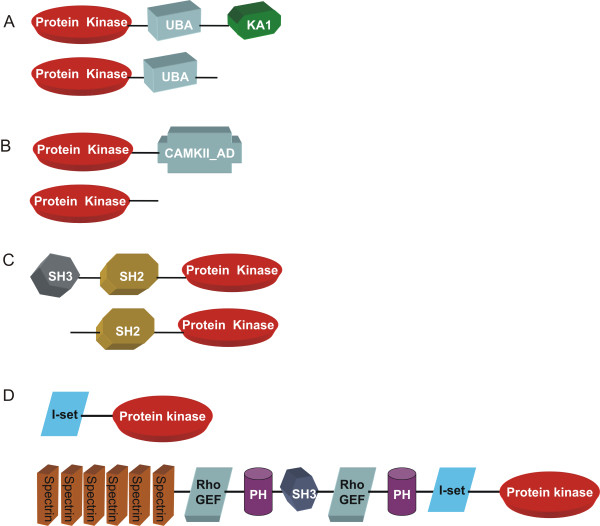
**Cartoon representation of domain organization of newly recognized splice variants encoded by a single gene which have different domain organization**. Splice variants shown in the figure are closely related to a) MARK kinase; b) CAMK II subfamily; c) Fyn tyrosine kinase d) Trio kinase. Abbreviations followed in the figure: UBA, Ubiquitin Associated Domain; KA1, Kinase Associated Domain 1; CAMKII_AD, Calcium/calmodulin dependent protein kinase II Association; SH3, Src homology 3; SH2, Src homology 2; I-set, Immunoglobulin domain; RhoGEF, Guanine nucleotide exchange factor for Rho/Rac/Cdc42-like GTPases; PH, Pleckstrin Homology domain.

Interestingly there are examples of newly recognized splice variants encoded by a single gene which differ markedly in length and have different domain organization. Such prominent differences indicate that these splice variants might have different mode of regulation and different functional properties. Additional File 2 represents set of such protein kinase splice variants which have different domain organisation. For example while two protein kinases (ENSP00000300843 and ENSP00000262893) with their catalytic domains closely resembling MARK subfamily has UBA (Ubiquitin-associated domain) domain following the kinase domain; however a splice variant (ENSP00000262891) has an extra domain, KA1 (Kinase-associated domain), apart from kinase and UBA domains (Figure 1A). KA1 domain tethered to protein kinase catalytic domain indicates a role of this kinase in ubiquitin pathway. Similarly, regulatory domain CAMII_AD domain which has role in multimerization is usually found tethered to the Calcium/Calmodulin dependent protein kinase II (CAMKII). CAMII_AD domain is absent in one of the splice variants which has a kinase domain closely related to CAMKII subfamily (ENSP00000369096) while this regulatory domain is present in three splice variants (ENSP00000339740, ENSP00000378032, ENSP00000378034) (Figure 1B). In the same way, one of the four splice variants (ENSP00000346671) encoded by the gene ENSG00000010810 lacks SH3 domain which is found tethered N-terminal to the Fyn tyrosine kinase (Figure 1C) which has role in increasing local concentration of proteins, altering their subcellular location and mediating the assembly of large multiprotein complexes. Interestingly, two splice variants (ENSP00000339291, ENSP00000339299) encoded by the gene ENSG00000038382 which belong to Trio subfamily of CAMK group regulated by calmodulin, may mediate apoptosis induced by interferon-gamma [36], have also different domain organizations. In this example one of the members (ENSP00000339299) has multiple domains like spectrin repeats, two copies of Rho GEF and PH domain, SH3 and I-set (Immunoglobulin) domains whereas another variant (ENSP00000339291) is lacking all these domains except I-set domain which is in the N-terminus of the kinase domain (Figure 1D). There are many more splice variants which are encoded by the same gene but lack one or more regulatory accessory domains and these are listed in Additional file 2.

Additional file 2 also lists few examples of splice variants which are encoded by same gene but their catalytic kinase domain belong to different protein kinase subfamilies. For example from a set of six splice variants (ENSP00000320622, ENSP00000346846, ENSP00000352088, ENSP00000353452, ENSP00000353530, ENSP00000355024) encoded by the gene ENSG00000065534 five splice variants belong to MLCK (Mixed Lineage Chain Kinase) subfamily whereas one of the splice variants belong to DRAK (death-associated protein kinase [DAP-kinase]-related apoptosis-inducing kinase) subfamily which plays important roles in apoptotic signal transduction [37]. There is another example in which one of the four splice variants (ENSP00000354170) encoded by ENSG00000068078 gene belong to Vascular Endothelial Growth Factor protein tyrosine kinase subfamily whereas other three (ENSP00000231803, ENSP00000260795, ENSP00000339824) belong to Fibroblast growth factor receptor subfamily (Additional file 2).

### Expression of human kinases splice variants in different cell types

Additional file 3 provides information on expression of many human kinase splice variants in different tissue or cell types. This information was obtained from ENSEMBL database. From this Table it can be observed that at least one splice variant product from each of 44 genes is expressed in two or more different tissues compared to other splice variants corresponding to the same gene. This number represents a proportion of ~49% of the genes implicated in variant splicing. In all such cases splice variants corresponding to a gene belong to the same kinase subfamily. For example, the gene ENSG00000141068 produces two kinases belonging to the same sub-family, one expressing in 4 tissues (ENSP00000323178) and the other expressing in 17 tissues (ENSP00000268763).

## Conclusions

Remarkable improvement in the quality of human genomic data enabled us to generate a more comprehensive repertoire of human kinases with identification of 918 protein kinase gene products with many of these representing splice variants with altered properties. Previous analyses on human kinome [1-3] presented much smaller set of kinases as splice variants were not analyzed in these early papers. Indeed Milensi et al [11] extended the early analysis of human kinome considering splice variation and, in particular, reported 5 further kinase genes and a pseudogene. However, present study indicates that the alternative splice forms of protein kinase genes are far more abundant than previously thought and seems to provide interesting variety with implications in biological pathways and processes. Present analysis has unearthed newly recognized protein kinase splice variants with almost all of them having known cDNA data available and many of them known to get expressed in various tissue or cell types. Our current survey enabled us to recognize many important kinase splice variants which might be involved in diverse signal transduction pathways and have different modes of regulation. List of newly recognized human kinase splice variants includes several examples which are not yet studied experimentally. Future direction could aim at studying sub-cellular localization of the kinase splice variants in the types of cells, their role in biological processes, modes of regulation and substrate specificity which will give insight into the human signal transduction pathway.

## Methods

The complete set of predicted protein sequences from the ORFs of the human genome has been obtained from ENSEMBL [release 52] http://www.ensembl.org. We have surveyed the genome, for Ser/Thr and Tyr protein kinases using sensitive sequence profile matching algorithms. The sequence search tools and other strategies for domain identification and sub-family classification used in this study by employing PSI-BLAST [38], Reverse PSI - BLAST (RPS-BLAST) [39], HMMer [40] which match Hidden Markov Models (HMMs) to identify protein kinase catalytic domain and their co-occurring domains. Search procedures such as PSI-BLAST and RPS-BLAST have been used at stringent E-value cut off of 0.0001. Searches performed using HMMer with an E-value cut-off of 0.01 or below have been shown to nearly eliminate any spurious hits. Hits lacking significant sequence similarity with the query have been further examined, manually. The final data set of predicted putative human protein kinases has been obtained from the compilation of hits obtained using various procedures. The program CD-HIT [41] was used in order to eliminate redundant sequences. The sequence identity shared between any two putative protein kinase sequences in the data set is less than 100%, suggesting that the data set is devoid of redundant sequences. Multiple sequence alignment of the kinase catalytic domains of the putative human protein kinases has been carried out using ClustalW [42]. Information on cell/tissue types where various kinases are expressed has been retrieved from ENSEMBL http://www.ensembl.org and it has been observed that 498 protein kinases are getting expressed in various tissue types (Additional file 3).

### Classification of human protein kinases into subfamilies

Protein kinases discussed in the current analysis have been classified into various protein kinase subfamilies proposed by Hanks and Hunter classification scheme. RPS-BLAST has been used to search each of the putative protein kinases as a query against the database containing 55 Multiple Position Specific Scoring Matrices (MulPSSMs) created for the various subgroups of protein kinases in each of the subfamilies. Criteria used in identifying kinase subfamilies involved use of stringent conditions such as alignment coverage of the sequence greater than 70% over the kinase domain region and sequence identity cut-off of 30% aside from satisfying the stringent e-value cutoff criteria.

Domain assignment to the non-catalytic regions of the kinase containing genes has been carried out using the HMM search methods by querying each of the kinase domain containing sequences against the 9318 protein family HMMs available in the Pfam database release 23 [43].

Information on splice variants for these putative protein kinases was taken from BioMart data mining tool available in ENSEMBL database which has entry for genes, their transcripts and protein products. In ENSEMBL, an initial alignment of mRNA and protein is performed against the genome. All overlapping transcripts are clustered under one gene name and deemed 'splice variants'. So the gene products pointing to a single gene are defined as splice variants by ENSEMBL http://www.ensembl.org.

## Authors' contributions

KA carried out the analysis. NG has developed the website. KA and NS have drafted the manuscript. All authors read and approved the final manuscript.

## Supplementary Material

Additional file 1 List of all the 918 human protein kinases. Click here for file

Additional file 2 List of human protein kinase splice variants with different domain organisations. Click here for file

Additional file 3 List of human kinase splice variants with information on their expression in various tissue/cell types obtained from ENSEMBL database. Click here for file

## References

[B1] Lander ES Linton LM Birren B Nusbaum C Zody MC Baldwin J Devon K Dewar K Doyle M FitzHugh W Funke R Gage D Harris K Heaford A Howland J Kann L Lehoczky J LeVine R McEwan P McKernan K Meldrim J Mesirov JP Miranda C Morris W Naylor J Raymond C Rosetti M Santos R Sheridan A Sougnez C Stange-Thomann N Stojanovic N Subramanian A Wyman D Rogers J Sulston J Ainscough R Beck S Bentley D Burton J Clee C Carter N Coulson A Deadman R Deloukas P Dunham A Dunham I Durbin R French L Grafham D Gregory S Hubbard T Humphray S Hunt A Jones M Lloyd C McMurray A Matthews L Mercer S Milne S Mullikin JC Mungall A Plumb R Ross M Shownkeen R Sims S Waterston RH Wilson RK Hillier LW McPherson JD Marra MA Mardis ER Fulton LA Chinwalla AT Pepin KH Gish WR Chissoe SL Wendl MC Delehaunty KD Miner TL Delehaunty A Kramer JB Cook LL Fulton RS Johnson DL Minx PJ Clifton SW Hawkins T Branscomb E Predki P Richardson P Wenning S Slezak T Doggett N Cheng JF Olsen A Lucas S Elkin C Uberbacher E Frazier M Gibbs RA Muzny DM Scherer SE Bouck JB Sodergren EJ Worley KC Rives CM Gorrell JH Metzker ML Naylor SL Kucherlapati RS Nelson DL Weinstock GM Sakaki Y Fujiyama A Hattori M Yada T Toyoda A Itoh T Kawagoe C Watanabe H Totoki Y Taylor T Weissenbach J Heilig R Saurin W Artiguenave F Brottier P Bruls T Pelletier E Robert C Wincker P Smith DR Doucette-Stamm L Rubenfield M Weinstock K Lee HM Dubois J Rosenthal A Platzer M Nyakatura G Taudien S Rump A Yang H Yu J Wang J Huang G Gu J Hood L Rowen L Madan A Qin S Davis RW Federspiel NA Abola AP Proctor MJ Myers RM Schmutz J Dickson M Grimwood J Cox DR Olson MV Kaul R Raymond C Shimizu N Kawasaki K Minoshima S Evans GA Athanasiou M Schultz R Roe BA Chen F Pan H Ramser J Lehrach H Reinhardt R McCombie WR de la Bastide M Dedhia N Blöcker H Hornischer K Nordsiek G Agarwala R Aravind L Bailey JA Bateman A Batzoglou S Birney E Bork P Brown DG Burge CB Cerutti L Chen HC Church D Clamp M Copley RR Doerks T Eddy SR Eichler EE Furey TS Galagan J Gilbert JG Harmon C Hayashizaki Y Haussler D Hermjakob H Hokamp K Jang W Johnson LS Jones TA Kasif S Kaspryzk A Kennedy S Kent WJ Kitts P Koonin EV Korf I Kulp D Lancet D Lowe TM McLysaght A Mikkelsen T Moran JV Mulder N Pollara VJ Ponting CP Schuler G Schultz J Slater G Smit AF Stupka E Szustakowski J Thierry-Mieg D Thierry-Mieg J Wagner L Wallis J Wheeler R Williams A Wolf YI Wolfe KH Yang SP Yeh RF Collins F Guyer MS Peterson J Felsenfeld A Wetterstrand KA Patrinos A Morgan MJ de Jong P Catanese JJ Osoegawa K Shizuya H Choi S Chen YJ Initial sequencing and analysis of the human genome Nature 2001 409 860 921 10.1038/35057062 11237011

[B2] Krupa A Srinivasan N The repertoire of protein kinases encoded in the draft version of the human genome: atypical variations and uncommon domain combinations Genome Biol 2002 3 RESEARCH0066 12537555 10.1186/gb-2002-3-12-research0066 PMC151168

[B3] Manning G Whyte DB Martinez R Hunter T Sudarsanam S The protein kinase complement of the human genome Science 2002 298 1912 1934 10.1126/science.1075762 12471243

[B4] Kostich M English J Madison V Gheyas F Wang L Qiu P Greene J Laz TM Human members of the eukaryotic protein kinase family Genome Biol 2002 3 RESEARCH0043 12225582 10.1186/gb-2002-3-9-research0043 PMC126868

[B5] Hanks SK Genomic analysis of the eukaryotic protein kinase superfamily: a perspective Genome Biol 2003 4 111 12734000 10.1186/gb-2003-4-5-111 PMC156577

[B6] Gilbert W Why genes in pieces? Nature 1978 271 501 10.1038/271501a0 622185

[B7] Wojtowicz WM Flanagan JJ Millard SS Zipursky SL Clemens JC Alternative splicing of Drosophila Dscam generates axon guidance receptors that exhibit isoform-specific homophilic binding Cell 2004 118 619 633 15339666 10.1016/j.cell.2004.08.021 PMC2691713

[B8] Forrest AR Taylor DF Crowe ML Chalk AM Waddell NJ Kolle G Faulkner GJ Kodzius R Katayama S Wells C Genome-wide review of transcriptional complexity in mouse protein kinases and phosphatases Genome Biol 2006 7 R5 16507138 10.1186/gb-2006-7-1-r5 PMC1431701

[B9] Wang BB Brendel V Genomewide comparative analysis of alternative splicing in plants Proc Natl Acad Sci USA 2006 103 7175 7180 16632598 10.1073/pnas.0602039103 PMC1459036

[B10] Ner-Gaon H Leviatan N Rubin E Fluhr R Comparative cross-species alternative splicing in plants Plant Physiol 2007 144 1632 1641 17496110 10.1104/pp.107.098640 PMC1914131

[B11] Milanesi L Petrillo M Sepe L Boccia A D'Agostino N Passamano M Di Nardo S Tasco G Casadio R Paolella G Systematic analysis of human kinase genes: a large number of genes and alternative splicing events result in functional and structural diversity BMC Bioinformatics 2005 6 Suppl 4 S20 16351747 10.1186/1471-2105-6-S4-S20 PMC1866387

[B12] Sorek R Shamir R Ast G How prevalent is functional alternative splicing in the human genome? Trends Genet 2004 20 68 71 10.1016/j.tig.2003.12.004 14746986

[B13] Nurtdinov RN Artamonova II Mironov AA Gelfand MS Low conservation of alternative splicing patterns in the human and mouse genomes Hum Mol Genet 2003 12 1313 1320 10.1093/hmg/ddg137 12761046

[B14] Modrek B Lee CJ Alternative splicing in the human, mouse and rat genomes is associated with an increased frequency of exon creation and/or loss Nat Genet 2003 34 177 180 10.1038/ng1159 12730695

[B15] Hunter T A thousand and one protein kinases Cell 1987 50 823 829 10.1016/0092-8674(87)90509-5 3113737

[B16] Modrek B Lee C A genomic view of alternative splicing Nat Genet 2002 30 13 19 10.1038/ng0102-13 11753382

[B17] Modrek B Resch A Grasso C Lee C Genome-wide detection of alternative splicing in expressed sequences of human genes Nucleic Acids Res 2001 29 2850 2859 11433032 10.1093/nar/29.13.2850 PMC55780

[B18] Mironov AA Fickett JW Gelfand MS Frequent alternative splicing of human genes Genome Res 1999 9 1288 1293 10613851 10.1101/gr.9.12.1288 PMC310997

[B19] Brett D Hanke J Lehmann G Haase S Delbruck S Krueger S Reich J Bork P EST comparison indicates 38% of human mRNAs contain possible alternative splice forms FEBS Lett 2000 474 83 86 10.1016/S0014-5793(00)01581-7 10828456

[B20] Garcia-Blanco MA Baraniak AP Lasda EL Alternative splicing in disease and therapy Nat Biotechnol 2004 22 535 546 10.1038/nbt964 15122293

[B21] Lopez AJ Alternative splicing of pre-mRNA: developmental consequences and mechanisms of regulation Annu Rev Genet 1998 32 279 305 10.1146/annurev.genet.32.1.279 9928482

[B22] Kamatkar S Radha V Nambirajan S Reddy RS Swarup G Two splice variants of a tyrosine phosphatase differ in substrate specificity, DNA binding, and subcellular location J Biol Chem 1996 271 26755 26761 10.1074/jbc.271.43.26755 8900155

[B23] Zhang J Gross SD Schroeder MD Anderson RA Casein kinase I alpha and alpha L: alternative splicing-generated kinases exhibit different catalytic properties Biochemistry 1996 35 16319 16327 10.1021/bi9614444 8973207

[B24] Hames RS Fry AM Alternative splice variants of the human centrosome kinase Nek2 exhibit distinct patterns of expression in mitosis Biochem J 2002 361 77 85 11742531 10.1042/0264-6021:3610077 PMC1222281

[B25] Scheper GC Parra JL Wilson M Van Kollenburg B Vertegaal AC Han ZG Proud CG The N and C termini of the splice variants of the human mitogen-activated protein kinase-interacting kinase Mnk2 determine activity and localization Mol Cell Biol 2003 23 5692 5705 12897141 10.1128/MCB.23.16.5692-5705.2003 PMC166352

[B26] Kreis P Rousseau V Thevenot E Combeau G Barnier JV The four mammalian splice variants encoded by the p21-activated kinase 3 gene have different biological properties J Neurochem 2008 106 1184 1197 10.1111/j.1471-4159.2008.05474.x 18507705

[B27] Brodbeck D Hill MM Hemmings BA Two splice variants of protein kinase B gamma have different regulatory capacity depending on the presence or absence of the regulatory phosphorylation site serine 472 in the carboxyl-terminal hydrophobic domain J Biol Chem 2001 276 29550 29558 10.1074/jbc.M104633200 11387345

[B28] Simon P Schneck M Hochstetter T Koutsouki E Mittelbronn M Merseburger A Weigert C Niess A Lang F Differential regulation of serum- and glucocorticoid-inducible kinase 1 (SGK1) splice variants based on alternative initiation of transcription Cell Physiol Biochem 2007 20 715 728 10.1159/000110432 17982254

[B29] Gunn-Moore FJ Williams AG Tavare JM Analysis of mitogen-activated protein kinase activation by naturally occurring splice variants of TrkC, the receptor for neurotrophin-3 Biochem J 1997 322 193 198 9078261 10.1042/bj3220193 PMC1218176

[B30] Stoilov P Meshorer E Gencheva M Glick D Soreq H Stamm S Defects in pre-mRNA processing as causes of and predisposition to diseases DNA Cell Biol 2002 21 803 818 10.1089/104454902320908450 12489991

[B31] Nissim-Rafinia M Kerem B Splicing regulation as a potential genetic modifier Trends Genet 2002 18 123 127 10.1016/S0168-9525(01)02619-1 11858835

[B32] Krupa A Abhinandan KR Srinivasan N KinG: a database of protein kinases in genomes Nucleic Acids Res 2004 32 D153 155 14681382 10.1093/nar/gkh019 PMC308754

[B33] Anand B Gowri VS Srinivasan N Use of multiple profiles corresponding to a sequence alignment enables effective detection of remote homologues Bioinformatics 2005 21 2821 2826 10.1093/bioinformatics/bti432 15817691

[B34] Gowri VS Krishnadev O Swamy CS Srinivasan N MulPSSM: a database of multiple position-specific scoring matrices of protein domain families Nucleic Acids Res 2006 34 D243 246 16381855 10.1093/nar/gkj043 PMC1347406

[B35] Hanks SK Hunter T Protein kinases 6. The eukaryotic protein kinase superfamily: kinase (catalytic) domain structure and classification Faseb J 1995 9 576 596 7768349

[B36] Debant A Serra-Pages C Seipel K O'Brien S Tang M Park SH Streuli M The multidomain protein Trio binds the LAR transmembrane tyrosine phosphatase, contains a protein kinase domain, and has separate rac-specific and rho-specific guanine nucleotide exchange factor domains Proc Natl Acad Sci USA 1996 93 5466 5471 8643598 10.1073/pnas.93.11.5466 PMC39269

[B37] Kogel D Prehn JH Scheidtmann KH The DAP kinase family of pro-apoptotic proteins: novel players in the apoptotic game Bioessays 2001 23 352 358 10.1002/bies.1050 11268041

[B38] Altschul SF Madden TL Schaffer AA Zhang J Zhang Z Miller W Lipman DJ Gapped BLAST and PSI-BLAST: a new generation of protein database search programs Nucleic Acids Res 1997 25 3389 3402 9254694 10.1093/nar/25.17.3389 PMC146917

[B39] Marchler-Bauer A Anderson JB DeWeese-Scott C Fedorova ND Geer LY He S Hurwitz DI Jackson JD Jacobs AR Lanczycki CJ Liebert CA Liu C Madej T Marchler GH Mazumder R Nikolskaya AN Panchenko AR Rao BS Shoemaker BA Simonyan V Song JS Thiessen PA Vasudevan S Wang Y Yamashita RA Yin JJ Bryant SH CDD: a curated Entrez database of conserved domain alignments Nucleic Acids Res 2003 31 383 387 12520028 10.1093/nar/gkg087 PMC165534

[B40] Eddy SR Profile hidden Markov models Bioinformatics 1998 14 755 763 10.1093/bioinformatics/14.9.755 9918945

[B41] Li W Jaroszewski L Godzik A Clustering of highly homologous sequences to reduce the size of large protein databases Bioinformatics 2001 17 282 283 10.1093/bioinformatics/17.3.282 11294794

[B42] Chenna R Sugawara H Koike T Lopez R Gibson TJ Higgins DG Thompson JD Multiple sequence alignment with the Clustal series of programs Nucleic Acids Res 2003 31 3497 3500 12824352 10.1093/nar/gkg500 PMC168907

[B43] Bateman A Birney E Cerruti L Durbin R Etwiller L Eddy SR Griffiths-Jones S Howe KL Marshall M Sonnhammer EL The Pfam protein families database Nucleic Acids Res 2002 30 276 280 11752314 10.1093/nar/30.1.276 PMC99071

