# Supplementary material for: Functional diversity of human protein kinase splice variants marks significant expansion of human kinome
Source: BMC Genomics. 2009 Dec 22;10:622. doi: 10.1186/1471-2164-10-622 (PMC2805699; doi:10.1186/1471-2164-10-622)
Supplement: Additional file 1 — List of all the 918 human protein kinases. [file 1471-2164-10-622-S1.DOC]

**Additional file 1:** List of all the 918 human protein kinases. Their gene accession codes, protein accession code, number of residues in the gene product, subfamily assignment and domain architecture are also provided. A web version of this Table with search options is available at <http://hodgkin.mbu.iisc.ernet.in/king/cgi/search>

| **Gene accession code** | **Protein accession code** | **Number of residues** | **Protein kinase Subfamily** | **Domain name and domain boundary** |
| --- | --- | --- | --- | --- |
| ENSG00000000938 | ENSP00000382126 | 529 | YES_Mm_TK_Src | SH3_1, 80 136 * SH3_2, 81 136 * SH2, 144 226 * Pkinase, 263 512 * |
| ENSG00000004660 | ENSP00000323118 | 505 | CAMKK1_Mm_Other_CAMKK | Pkinase, 128 409 * |
| ENSG00000004660 | ENSP00000371188 | 532 | CAMKK1_Mm_Other_CAMKK | Pkinase, 155 436 * |
| ENSG00000004660 | ENSP00000371190 | 543 | CAMKK1_Mm_Other_CAMKK | Pkinase, 128 447 * |
| ENSG00000006062 | ENSP00000342059 | 946 | MAP3K1_Hs_STE_STE11 | Pkinase, 398 650 * |
| ENSG00000006062 | ENSP00000366125 | 433 | MAP3K1_Hs_STE_STE11 | Pkinase, 182 415 * |
| ENSG00000006432 | ENSP00000005198 | 1118 | MLK1_Mm_TKL_MLK | SH3_1, 55 114 * SH3_2, 56 114 * Pkinase, 144 403 * |
| ENSG00000006432 | ENSP00000370649 | 1104 | MLK1_Mm_TKL_MLK | SH3_1, 55 114 * SH3_2, 56 114 * Pkinase, 144 403 * |
| ENSG00000006837 | ENSP00000265334 | 592 | CDKL3_Mm_CMGC_CDKL | Pkinase, 4 286 * |
| ENSG00000007047 | ENSP00000262891 | 752 | MARK1_Hs_CAMK_CAMKL | Pkinase, 59 310 * UBA, 330 366 * KA1, 706 752 * |
| ENSG00000007047 | ENSP00000262893 | 462 | MARK2_Hs_CAMK_CAMKL | Pkinase, 59 340 * UBA, 360 396 * |
| ENSG00000007047 | ENSP00000300843 | 688 | MARK2_Hs_CAMK_CAMKL | Pkinase, 59 310 * UBA, 330 366 * |
| ENSG00000007264 | ENSP00000308734 | 507 | CTK_Mm_TK_Csk | SH2, 122 196 * Pkinase, 235 478 * |
| ENSG00000007264 | ENSP00000378481 | 466 | CTK_Mm_TK_Csk | SH2, 81 155 * Pkinase, 194 437 * |
| ENSG00000007264 | ENSP00000378485 | 508 | CTK_Mm_TK_Csk | SH2, 123 197 * Pkinase, 236 479 * |
| ENSG00000008086 | ENSP00000369325 | 1030 | CDKL5_Hs_CMGC_CDKL | Pkinase, 13 297 * |
| ENSG00000008118 | ENSP00000354861 | 476 | CaMK1g_Hs_CAMK_CAMK1 | Pkinase, 23 277 * |
| ENSG00000008128 | ENSP00000348529 | 746 | PITSLRE_Mm_CMGC_CDK | Pkinase, 389 674 * |
| ENSG00000008128 | ENSP00000350403 | 779 | PITSLRE_Mm_CMGC_CDK | Pkinase, 422 707 * |
| ENSG00000008128 | ENSP00000367900 | 783 | PITSLRE_Mm_CMGC_CDK | Pkinase, 426 711 * |
| ENSG00000008128 | ENSP00000367902 | 770 | PITSLRE_Mm_CMGC_CDK | Pkinase, 413 698 * |
| ENSG00000008128 | ENSP00000383875 | 780 | PITSLRE_Mm_CMGC_CDK | Pkinase, 423 708 * |
| ENSG00000010219 | ENSP00000010132 | 520 | 5306_Tt_CMGC_DYRK | Pkinase, 104 400 * |
| ENSG00000010671 | ENSP00000308176 | 659 | BTK_Mm_TK_Tec | PH, 4 133 * BTK, 140 171 * SH3_1, 217 272 * SH3_2, 218 272 * SH2, 281 362 * Pkinase, 402 651 * |
| ENSG00000010671 | ENSP00000361946 | 252 | BTK_Mm_TK_Tec | SH2, 1 77 * Pkinase, 7 244 * |
| ENSG00000010810 | ENSP00000346671 | 485 | FYN_Hs_TK_Src | SH2, 100 182 * Pkinase, 219 468 * |
| ENSG00000010810 | ENSP00000348295 | 482 | FYN_Hs_TK_Src | SH3_1, 85 141 * SH3_2, 86 141 * SH2, 149 231 * Pkinase, 221 465 * |
| ENSG00000010810 | ENSP00000357656 | 537 | FYN_Hs_TK_Src | SH3_1, 85 141 * SH3_2, 86 141 * SH2, 149 231 * Pkinase, 271 520 * |
| ENSG00000010810 | ENSP00000357671 | 534 | FYN_Hs_TK_Src | SH3_1, 85 141 * SH3_2, 86 141 * SH2, 149 231 * Pkinase, 268 517 * |
| ENSG00000011566 | ENSP00000263881 | 894 | KHS2_Hs_STE_STE20 | Pkinase, 16 273 * CNH, 562 874 * |
| ENSG00000011566 | ENSP00000345434 | 873 | KHS2_Hs_STE_STE20 | Pkinase, 16 273 * CNH, 541 853 * |
| ENSG00000012983 | ENSP00000013125 | 846 | KHS1_Mm_STE_STE20 | Pkinase, 20 277 * CNH, 512 826 * |
| ENSG00000013441 | ENSP00000326830 | 484 | CLK1_Mm_CMGC_CLK | Pkinase, 161 477 * |
| ENSG00000013441 | ENSP00000386875 | 526 | CLK1_Mm_CMGC_CLK | Pkinase, 203 519 * |
| ENSG00000013441 | ENSP00000386358 | 307 | CLK1_Mm_CMGC_CLK | Pkinase, 2 300 * |
| ENSG00000027075 | ENSP00000329127 | 683 | PKCeta_Mm_AGC_PKC | C2, 12 102 * C1_1, 172 225 * C1_1, 246 298 * Pkinase, 355 614 * Pkinase_C, 634 680 * |
| ENSG00000027644 | ENSP00000357178 | 1297 | IGF1R_Hs_TK_InsR | Recep_L_domain, 47 159 * Furin-like, 173 329 * Recep_L_domain, 346 461 * fn3, 605 795 * fn3, 815 902 * Pkinase, 979 1246 * |
| ENSG00000028116 | ENSP00000342381 | 508 | VRK2_Mm_CK1_VRK | Pkinase, 29 292 * |
| ENSG00000028116 | ENSP00000378045 | 400 | VRK2_Mm_CK1_VRK | Pkinase, 29 292 * |
| ENSG00000030304 | ENSP00000189978 | 867 | MUSK_Mm_TK_Musk | I-set, 28 117 * ig, 42 101 * V-set, 119 209 * I-set, 121 208 * ig, 135 192 * I-set, 212 299 * ig, 226 284 * Fz, 317 454 * Pkinase, 573 857 * |
| ENSG00000030304 | ENSP00000363570 | 783 | MUSK_Mm_TK_Musk | I-set, 28 117 * ig, 42 101 * V-set, 119 209 * I-set, 121 208 * ig, 135 192 * I-set, 222 309 * ig, 236 294 * Fz, 269 370 * Pkinase, 489 773 * |
| ENSG00000030304 | ENSP00000363571 | 875 | MUSK_Mm_TK_Musk | I-set, 28 117 * ig, 42 101 * V-set, 119 209 * I-set, 121 208 * ig, 135 192 * I-set, 212 299 * ig, 226 284 * Fz, 317 454 * Pkinase, 581 865 * |
| ENSG00000034152 | ENSP00000319139 | 351 | MAP2K3_Mm_STE_STE7 | Pkinase, 68 329 * |
| ENSG00000034152 | ENSP00000345083 | 347 | MAP2K3_Mm_STE_STE7 | Pkinase, 64 325 * |
| ENSG00000034152 | ENSP00000378870 | 318 | MAP2K3_Mm_STE_STE7 | Pkinase, 35 296 * |
| ENSG00000035664 | ENSP00000379136 | 370 | DAPK2_Mm_CAMK_DAPK | Pkinase, 23 285 * |
| ENSG00000037280 | ENSP00000261937 | 1363 | FLT4_Mm_TK_VEGFR | ig, 44 113 * I-set, 230 327 * V-set, 231 328 * ig, 245 312 * ig, 349 401 * V-set, 557 675 * ig, 571 655 * V-set, 677 778 * I-set, 678 765 * ig, 692 750 * Pkinase, 845 1169 * |
| ENSG00000037280 | ENSP00000377016 | 1298 | FLT4_Mm_TK_VEGFR | ig, 44 113 * I-set, 230 327 * V-set, 231 328 * ig, 245 312 * ig, 349 401 * V-set, 557 675 * ig, 571 655 * V-set, 677 778 * I-set, 678 765 * ig, 692 750 * Pkinase, 845 1169 * |
| ENSG00000038382 | ENSP00000339291 | 596 | Trio_Hs_CAMK_Trio | I-set, 184 275 * V-set, 184 276 * ig, 198 260 * Pkinase, 295 549 * |
| ENSG00000038382 | ENSP00000339299 | 3097 | Trio_Hs_CAMK_Trio | Spectrin, 218 338 * Spectrin, 340 446 * Spectrin, 566 670 * Spectrin, 671 784 * Spectrin, 907 1012 * Spectrin, 1138 1244 * RhoGEF, 1296 1466 * PH, 1480 1591 * SH3_1, 1659 1719 * SH3_2, 1660 1719 * RhoGEF, 1973 2144 * PH, 2158 2271 * GRP, 2240 2324 * I-set, 2685 2776 * V-set, 2685 2777 * ig, 2699 2761 * Pkinase, 2796 3050 * |
| ENSG00000044524 | ENSP00000337451 | 983 | EphB4_Mm_TK_Eph | Ephrin_lbd, 29 202 * GCC2_GCC3, 255 304 * fn3, 326 419 * fn3, 437 521 * Pkinase, 621 878 * SAM_2, 908 975 * SAM_1, 909 973 * |
| ENSG00000047936 | ENSP00000357493 | 2341 | ROS_Mm_TK_Sev | fn3, 108 192 * fn3, 203 284 * fn3, 567 656 * fn3, 943 1025 * fn3, 1036 1135 * fn3, 1552 1642 * fn3, 1653 1739 * Pkinase, 1939 2211 * |
| ENSG00000047936 | ENSP00000357494 | 2347 | ROS_Mm_TK_Sev | fn3, 99 183 * fn3, 194 275 * fn3, 558 661 * fn3, 948 1030 * fn3, 1041 1140 * fn3, 1558 1648 * fn3, 1659 1745 * Pkinase, 1945 2217 * |
| ENSG00000050748 | ENSP00000345524 | 382 | JNK3_Hs_CMGC_MAPK | Pkinase, 26 321 * |
| ENSG00000050748 | ENSP00000321410 | 424 | JNK3_Hs_CMGC_MAPK | Pkinase, 26 321 * |
| ENSG00000050748 | ENSP00000377028 | 382 | JNK3_Hs_CMGC_MAPK | Pkinase, 26 321 * |
| ENSG00000050748 | ENSP00000377029 | 424 | JNK3_Hs_CMGC_MAPK | Pkinase, 26 321 * |
| ENSG00000055332 | ENSP00000341954 | 510 | PKR_Hs_Other_PEK | dsrm, 10 75 * dsrm, 101 165 * Pkinase, 171 495 * |
| ENSG00000055332 | ENSP00000368453 | 551 | PKR_Hs_Other_PEK | dsrm, 10 75 * dsrm, 101 165 * Pkinase, 267 536 * |
| ENSG00000058091 | ENSP00000265741 | 451 | PFTAIRE1_Mm_CMGC_CDK | Pkinase, 117 401 * |
| ENSG00000058091 | ENSP00000369390 | 469 | PFTAIRE1_Mm_CMGC_CDK | Pkinase, 135 419 * |
| ENSG00000058091 | ENSP00000385034 | 423 | PFTAIRE1_Mm_CMGC_CDK | Pkinase, 89 373 * |
| ENSG00000058404 | ENSP00000326518 | 449 | CaMK2g_Hs_CAMK_CAMK2 | Pkinase, 14 272 * CaMKII_AD, 317 444 * |
| ENSG00000058404 | ENSP00000326544 | 492 | CaMK2g_Hs_CAMK_CAMK2 | Pkinase, 14 272 * CaMKII_AD, 386 487 * |
| ENSG00000058404 | ENSP00000326375 | 542 | CaMK2g_Hs_CAMK_CAMK2 | Pkinase, 14 272 * CaMKII_AD, 410 537 * |
| ENSG00000058404 | ENSP00000326600 | 518 | CaMK2g_Hs_CAMK_CAMK2 | Pkinase, 14 272 * CaMKII_AD, 386 513 * |
| ENSG00000058404 | ENSP00000326427 | 479 | CaMK2g_Hs_CAMK_CAMK2 | Pkinase, 14 272 * CaMKII_AD, 347 474 * |
| ENSG00000058404 | ENSP00000351542 | 503 | CaMK2g_Hs_CAMK_CAMK2 | Pkinase, 14 272 * CaMKII_AD, 371 498 * |
| ENSG00000058404 | ENSP00000379096 | 517 | CaMK2g_Hs_CAMK_CAMK2 | Pkinase, 14 272 * CaMKII_AD, 385 512 * |
| ENSG00000058404 | ENSP00000379098 | 666 | CaMK2g_Hs_CAMK_CAMK2 | Pkinase, 14 272 * CaMKII_AD, 534 661 * |
| ENSG00000059758 | ENSP00000261211 | 523 | PCTAIRE2_Mm_CMGC_CDK | Pkinase, 192 473 * |
| ENSG00000060140 | ENSP00000075503 | 422 | TK1_Sp_TK_TK-Sp1 | Pkinase, 114 380 * |
| ENSG00000060237 | ENSP00000252477 | 2136 | Wnk3_Mm_Other_WNK | Pkinase, 221 479 * |
| ENSG00000060237 | ENSP00000313059 | 2382 | Wnk3_Mm_Other_WNK | Pkinase, 221 479 * |
| ENSG00000061938 | ENSP00000323216 | 528 | ACK_Mm_TK_Ack | Pkinase, 126 385 * SH3_1, 391 446 * GTPase_binding, 447 504 * |
| ENSG00000061938 | ENSP00000371341 | 1086 | ACK_Mm_TK_Ack | Pkinase, 189 448 * SH3_1, 454 509 * GTPase_binding, 510 577 * |
| ENSG00000061938 | ENSP00000376201 | 1038 | ACK_Mm_TK_Ack | Pkinase, 126 385 * SH3_1, 391 446 * GTPase_binding, 447 514 * |
| ENSG00000062524 | ENSP00000263800 | 864 | ALK_Mm_TK_ALK | Pkinase, 510 777 * |
| ENSG00000062524 | ENSP00000347293 | 803 | ALK_Mm_TK_ALK | Pkinase, 449 716 * |
| ENSG00000064393 | ENSP00000263551 | 1198 | HIPK2_Mm_CMGC_DYRK | Pkinase, 199 527 * |
| ENSG00000064393 | ENSP00000343108 | 918 | HIPK2_Mm_CMGC_DYRK | Pkinase, 199 527 * |
| ENSG00000065243 | ENSP00000359552 | 984 | PKN2_Mm_AGC_PKN | HR1, 47 119 * HR1, 136 213 * HR1, 217 294 * Pkinase, 657 916 * Pkinase_C, 936 983 * |
| ENSG00000065534 | ENSP00000320622 | 1845 | smMLCK_Hs_CAMK_MLCK | I-set, 33 123 * V-set, 33 120 * ig, 47 108 * I-set, 161 250 * V-set, 161 262 * ig, 175 235 * I-set, 445 531 * V-set, 447 532 * ig, 459 516 * I-set, 554 643 * V-set, 554 644 * ig, 568 628 * I-set, 652 742 * V-set, 652 743 * ig, 666 727 * I-set, 1029 1118 * V-set, 1029 1119 * ig, 1043 1103 * V-set, 1168 1259 * I-set, 1169 1258 * ig, 1183 1243 * fn3, 1262 1347 * Pkinase, 1395 1650 * I-set, 1740 1830 * V-set, 1740 1831 * ig, 1754 1815 * |
| ENSG00000065534 | ENSP00000346846 | 1845 | smMLCK_Hs_CAMK_MLCK | I-set, 33 123 * V-set, 33 120 * ig, 47 108 * I-set, 161 250 * V-set, 161 272 * ig, 175 235 * I-set, 445 531 * V-set, 447 532 * ig, 459 516 * I-set, 554 643 * V-set, 554 644 * ig, 568 628 * I-set, 652 742 * V-set, 652 743 * ig, 666 727 * I-set, 1029 1118 * V-set, 1029 1119 * ig, 1043 1103 * V-set, 1168 1259 * I-set, 1169 1258 * ig, 1183 1243 * fn3, 1262 1347 * Pkinase, 1395 1650 * I-set, 1740 1830 * V-set, 1740 1831 * ig, 1754 1815 * |
| ENSG00000065534 | ENSP00000352088 | 1863 | smMLCK_Hs_CAMK_MLCK | I-set, 33 123 * V-set, 33 120 * ig, 47 108 * I-set, 161 250 * V-set, 161 272 * ig, 175 235 * V-set, 349 505 * I-set, 414 504 * ig, 428 489 * I-set, 514 600 * V-set, 516 601 * ig, 528 585 * I-set, 623 712 * V-set, 623 713 * ig, 637 697 * I-set, 721 811 * V-set, 721 812 * ig, 735 796 * I-set, 1098 1187 * V-set, 1098 1188 * ig, 1112 1172 * V-set, 1237 1328 * I-set, 1238 1327 * ig, 1252 1312 * fn3, 1331 1416 * Pkinase, 1464 1668 * I-set, 1758 1848 * V-set, 1758 1849 * ig, 1772 1833 * |
| ENSG00000065534 | ENSP00000353452 | 1914 | smMLCK_Hs_CAMK_MLCK | I-set, 33 123 * V-set, 33 120 * ig, 47 108 * I-set, 161 250 * V-set, 161 262 * ig, 175 235 * V-set, 349 505 * I-set, 414 504 * ig, 428 489 * I-set, 514 600 * V-set, 516 601 * ig, 528 585 * I-set, 623 712 * V-set, 623 713 * ig, 637 697 * I-set, 721 811 * V-set, 721 812 * ig, 735 796 * I-set, 1098 1187 * V-set, 1098 1188 * ig, 1112 1172 * V-set, 1237 1328 * I-set, 1238 1327 * ig, 1252 1312 * fn3, 1331 1416 * Pkinase, 1464 1719 * I-set, 1809 1899 * V-set, 1809 1900 * ig, 1823 1884 * |
| ENSG00000065534 | ENSP00000353530 | 1841 | DRAK_Sp_CAMK_DAPK | I-set, 33 123 * V-set, 33 120 * ig, 47 108 * I-set, 161 250 * V-set, 161 262 * ig, 175 235 * V-set, 349 505 * I-set, 414 504 * ig, 428 489 * I-set, 514 600 * V-set, 516 601 * ig, 528 585 * I-set, 623 712 * V-set, 623 713 * ig, 637 697 * I-set, 721 811 * V-set, 721 812 * ig, 735 796 * I-set, 1098 1187 * V-set, 1098 1188 * ig, 1112 1172 * V-set, 1237 1328 * I-set, 1238 1327 * ig, 1252 1312 * fn3, 1331 1416 * Pkinase, 1421 1646 * I-set, 1736 1826 * V-set, 1736 1827 * ig, 1750 1811 * |
| ENSG00000065534 | ENSP00000355024 | 992 | smMLCK_Hs_CAMK_MLCK | I-set, 176 265 * V-set, 176 266 * ig, 190 250 * V-set, 315 406 * I-set, 316 405 * ig, 330 390 * fn3, 409 494 * Pkinase, 542 797 * I-set, 887 977 * V-set, 887 978 * ig, 901 962 * |
| ENSG00000065559 | ENSP00000262445 | 399 | MKK4_Sp_STE_STE7 | Pkinase, 102 367 * |
| ENSG00000065613 | ENSP00000336824 | 1204 | SLK_Mm_STE_STE20 | Pkinase, 34 292 * |
| ENSG00000065613 | ENSP00000358770 | 1235 | SLK_Mm_STE_STE20 | Pkinase, 34 292 * |
| ENSG00000065675 | ENSP00000380360 | 478 | PKCt_Mm_AGC_PKC | Pkinase, 152 406 * Pkinase_C, 426 472 * |
| ENSG00000065675 | ENSP00000380361 | 643 | AktA_Sp_AGC_Akt | C1_1, 160 212 * C1_3, 171 202 * C1_1, 232 284 * C1_3, 243 274 * Pkinase, 380 627 * Pkinase_C, 591 637 * |
| ENSG00000065675 | ENSP00000380363 | 706 | PKCt_Mm_AGC_PKC | C1_1, 160 212 * C1_3, 171 202 * C1_1, 232 284 * C1_3, 243 274 * Pkinase, 380 634 * Pkinase_C, 654 700 * |
| ENSG00000065883 | ENSP00000181839 | 1512 | CRK7_Mm_CMGC_CDK | Pkinase, 705 998 * |
| ENSG00000065883 | ENSP00000313986 | 324 | CRK7_Mm_CMGC_CDK | Pkinase, 91 324 * |
| ENSG00000065883 | ENSP00000340557 | 1452 | CRK7_Mm_CMGC_CDK | Pkinase, 705 998 * |
| ENSG00000066056 | ENSP00000361553 | 541 | TIE1_Mm_TK_Tie | fn3, 47 132 * Pkinase, 242 510 * |
| ENSG00000066056 | ENSP00000361554 | 1138 | TIE1_Mm_TK_Tie | ig, 139 197 * EGF, 224 255 * EGF_2, 228 255 * Laminin_EGF, 231 272 * EGF, 315 344 * EGF_2, 319 344 * ig, 365 428 * fn3, 446 533 * fn3, 546 632 * fn3, 644 729 * Pkinase, 839 1107 * |
| ENSG00000066468 | ENSP00000337665 | 785 | FGFR2_Mm_TK_FGFR | V-set, 41 125 * ig, 55 109 * V-set, 159 249 * I-set, 160 248 * ig, 172 233 * I-set, 256 359 * V-set, 256 360 * ig, 271 344 * Pkinase, 479 755 * |
| ENSG00000066468 | ENSP00000263454 | 731 | FGFR2_Mm_TK_FGFR | V-set, 70 160 * I-set, 71 159 * ig, 83 144 * I-set, 167 268 * V-set, 167 269 * ig, 182 253 * Pkinase, 391 667 * |
| ENSG00000066468 | ENSP00000263451 | 819 | FGFR2_Mm_TK_FGFR | V-set, 41 125 * ig, 55 109 * V-set, 159 249 * I-set, 160 248 * ig, 172 233 * I-set, 256 359 * V-set, 256 360 * ig, 271 344 * Pkinase, 479 755 * |
| ENSG00000066468 | ENSP00000309878 | 841 | FGFR2_Mm_TK_FGFR | V-set, 60 144 * ig, 74 128 * V-set, 178 268 * I-set, 179 267 * ig, 191 252 * I-set, 275 376 * V-set, 275 377 * ig, 290 361 * Pkinase, 501 777 * |
| ENSG00000066468 | ENSP00000348559 | 704 | FGFR2_Mm_TK_FGFR | V-set, 44 134 * I-set, 45 133 * ig, 57 118 * I-set, 141 244 * V-set, 141 245 * ig, 156 229 * Pkinase, 364 640 * |
| ENSG00000066468 | ENSP00000350166 | 840 | FGFR2_Mm_TK_FGFR | V-set, 60 144 * ig, 74 128 * V-set, 178 268 * I-set, 179 267 * ig, 191 252 * I-set, 275 378 * V-set, 275 379 * ig, 290 363 * Pkinase, 500 776 * |
| ENSG00000066468 | ENSP00000353262 | 680 | FGFR2_Mm_TK_FGFR | V-set, 70 160 * I-set, 71 159 * ig, 83 144 * I-set, 167 268 * V-set, 167 269 * ig, 182 253 * Pkinase, 393 669 * |
| ENSG00000066468 | ENSP00000358054 | 772 | FGFR2_Mm_TK_FGFR | V-set, 41 125 * ig, 55 109 * V-set, 159 249 * I-set, 160 248 * ig, 172 233 * I-set, 256 357 * V-set, 256 358 * ig, 271 342 * Pkinase, 482 758 * |
| ENSG00000066468 | ENSP00000358055 | 707 | FGFR2_Mm_TK_FGFR | V-set, 44 134 * I-set, 45 133 * ig, 57 118 * I-set, 141 242 * V-set, 141 243 * ig, 156 227 * Pkinase, 367 643 * |
| ENSG00000066468 | ENSP00000358056 | 705 | FGFR2_Mm_TK_FGFR | V-set, 41 125 * ig, 55 109 * V-set, 159 249 * I-set, 160 248 * ig, 172 233 * Pkinase, 365 641 * |
| ENSG00000066468 | ENSP00000358057 | 709 | FGFR2_Mm_TK_FGFR | V-set, 41 125 * ig, 55 109 * V-set, 159 249 * I-set, 160 248 * ig, 172 233 * Pkinase, 369 645 * |
| ENSG00000066468 | ENSP00000358058 | 822 | FGFR2_Mm_TK_FGFR | V-set, 41 125 * ig, 55 109 * V-set, 159 249 * I-set, 160 248 * ig, 172 233 * I-set, 256 362 * V-set, 256 363 * ig, 271 347 * Pkinase, 482 758 * |
| ENSG00000067606 | ENSP00000367830 | 592 | PKCz_Hs_AGC_PKC | PB1, 15 98 * C1_1, 131 183 * Pkinase, 252 518 * Pkinase_C, 538 584 * |
| ENSG00000067606 | ENSP00000383712 | 409 | PKCz_Hs_AGC_PKC | Pkinase, 69 335 * Pkinase_C, 355 401 * |
| ENSG00000067900 | ENSP00000261535 | 1354 | ROCK2_Mm_AGC_DMPK | Pkinase, 76 338 * Pkinase_C, 358 403 * HR1, 458 542 * Rho_Binding, 948 1014 * PH, 1119 1317 * C1_1, 1229 1284 * |
| ENSG00000067900 | ENSP00000382697 | 1354 | ROCK2_Mm_AGC_DMPK | Pkinase, 76 338 * Pkinase_C, 358 403 * HR1, 458 542 * Rho_Binding, 948 1014 * PH, 1119 1317 * C1_1, 1229 1284 * |
| ENSG00000068078 | ENSP00000260795 | 806 | FGFR3_Mm_TK_FGFR | ig, 54 111 * V-set, 122 246 * I-set, 157 245 * ig, 169 230 * I-set, 253 356 * V-set, 253 357 * ig, 268 341 * Pkinase, 472 748 * |
| ENSG00000068078 | ENSP00000339824 | 808 | FGFR3_Mm_TK_FGFR | ig, 54 111 * V-set, 122 246 * I-set, 157 245 * ig, 169 230 * I-set, 253 355 * V-set, 253 356 * ig, 268 340 * Pkinase, 474 750 * |
| ENSG00000068078 | ENSP00000231803 | 694 | FGFR3_Mm_TK_FGFR | ig, 54 111 * V-set, 122 246 * I-set, 157 245 * ig, 169 230 * Pkinase, 360 636 * |
| ENSG00000068078 | ENSP00000354170 | 879 | FLT4_Mm_TK_VEGFR | ig, 141 198 * V-set, 209 333 * I-set, 244 332 * ig, 256 317 * I-set, 340 443 * V-set, 340 444 * ig, 355 428 * Pkinase, 560 875 * |
| ENSG00000069020 | ENSP00000261569 | 2432 | MAST3_Mm_AGC_MAST | DUF1908, 62 341 * Pkinase, 379 652 * PDZ, 949 1035 * |
| ENSG00000069020 | ENSP00000370294 | 2444 | MAST3_Mm_AGC_MAST | DUF1908, 74 353 * Pkinase, 391 664 * PDZ, 961 1047 * |
| ENSG00000069020 | ENSP00000385727 | 2623 | MAST3_Mm_AGC_MAST | DUF1908, 256 532 * Pkinase, 570 843 * PDZ, 1140 1226 * |
| ENSG00000069020 | ENSP00000384313 | 2434 | MAST3_Mm_AGC_MAST | DUF1908, 67 343 * Pkinase, 381 654 * PDZ, 951 1037 * |
| ENSG00000069020 | ENSP00000385048 | 2429 | MAST3_Mm_AGC_MAST | DUF1908, 62 338 * Pkinase, 376 649 * PDZ, 946 1032 * |
| ENSG00000069020 | ENSP00000384099 | 2444 | MAST3_Mm_AGC_MAST | DUF1908, 74 353 * Pkinase, 391 664 * PDZ, 961 1047 * |
| ENSG00000069956 | ENSP00000261845 | 721 | ERK3_Mm_CMGC_MAPK | Pkinase, 20 316 * |
| ENSG00000070759 | ENSP00000361155 | 555 | TESK2_Hs_TKL_LISK | Pkinase, 59 293 * |
| ENSG00000070759 | ENSP00000361156 | 542 | TESK2_Hs_TKL_LISK | Pkinase, 59 335 * |
| ENSG00000070759 | ENSP00000361158 | 571 | TESK2_Hs_TKL_LISK | Pkinase, 59 309 * |
| ENSG00000070770 | ENSP00000262506 | 350 | CK2a2_Mm_CMGC_CK2 | Pkinase, 40 325 * APH, 42 223 * |
| ENSG00000070808 | ENSP00000261793 | 529 | CaMK2g_Hs_CAMK_CAMK2 | Pkinase, 64 322 * CaMKII_AD, 397 524 * |
| ENSG00000070808 | ENSP00000305090 | 489 | CaMK2g_Hs_CAMK_CAMK2 | Pkinase, 13 271 * CaMKII_AD, 357 484 * |
| ENSG00000070808 | ENSP00000381411 | 478 | CaMK2g_Hs_CAMK_CAMK2 | Pkinase, 13 271 * CaMKII_AD, 346 473 * |
| ENSG00000070808 | ENSP00000381412 | 540 | CaMK2g_Hs_CAMK_CAMK2 | Pkinase, 64 322 * CaMKII_AD, 408 535 * |
| ENSG00000070886 | ENSP00000166244 | 1005 | EphB4_Mm_TK_Eph | Ephrin_lbd, 31 204 * fn3, 329 422 * fn3, 437 524 * Pkinase, 635 892 * SAM_2, 927 994 * SAM_1, 931 992 * |
| ENSG00000071054 | ENSP00000313644 | 1320 | HGK_Hs_STE_STE20 | Pkinase, 25 289 * CNH, 1007 1298 * |
| ENSG00000071054 | ENSP00000314363 | 1239 | HGK_Hs_STE_STE20 | Pkinase, 25 289 * CNH, 926 1217 * |
| ENSG00000071054 | ENSP00000281111 | 1166 | HGK_Hs_STE_STE20 | Pkinase, 25 289 * CNH, 845 1144 * |
| ENSG00000071054 | ENSP00000343658 | 1242 | HGK_Hs_STE_STE20 | Pkinase, 25 289 * CNH, 921 1220 * |
| ENSG00000071242 | ENSP00000265678 | 733 | RSK1_Mm_AGC_RSK | Pkinase, 59 318 * Pkinase_C, 338 382 * Pkinase, 415 672 * |
| ENSG00000071242 | ENSP00000355831 | 741 | RSK1_Mm_AGC_RSK | Pkinase, 67 326 * Pkinase_C, 346 390 * Pkinase, 423 680 * |
| ENSG00000071242 | ENSP00000386050 | 758 | RSK1_Mm_AGC_RSK | Pkinase, 84 343 * Pkinase_C, 363 407 * Pkinase, 440 697 * |
| ENSG00000071575 | ENSP00000155926 | 343 | Trb2_Mm_CAMK_Trbl | Pkinase, 64 308 * |
| ENSG00000071575 | ENSP00000370874 | 207 | Trb2_Mm_CAMK_Trbl | Pkinase, 1 172 * |
| ENSG00000071909 | ENSP00000314213 | 1277 | MYO3B_Mm_STE_STE20 | Pkinase, 26 292 * Myosin_head, 344 1045 * IQ, 1060 1080 * IQ, 1087 1107 * |
| ENSG00000071909 | ENSP00000314650 | 1250 | MYO3B_Mm_STE_STE20 | Pkinase, 26 292 * Myosin_head, 344 1045 * |
| ENSG00000071909 | ENSP00000335100 | 1274 | MYO3B_Mm_STE_STE20 | Pkinase, 26 292 * Myosin_head, 344 1045 * IQ, 1060 1080 * IQ, 1087 1107 * |
| ENSG00000071909 | ENSP00000386213 | 1341 | MYO3B_Mm_STE_STE20 | Pkinase, 27 293 * Myosin_head, 345 1046 * IQ, 1061 1081 * IQ, 1088 1108 * |
| ENSG00000071909 | ENSP00000386497 | 1314 | MYO3B_Mm_STE_STE20 | Pkinase, 27 293 * Myosin_head, 345 1046 * |
| ENSG00000071909 | ENSP00000386573 | 1191 | MYO3B_Mm_STE_STE20 | Pkinase, 27 293 * Myosin_head, 345 1045 * IQ, 1060 1080 * IQ, 1087 1107 * |
| ENSG00000072062 | ENSP00000309591 | 351 | PKACa_Hs_AGC_PKA | Pkinase, 44 298 * |
| ENSG00000072062 | ENSP00000340940 | 343 | PKACa_Hs_AGC_PKA | Pkinase, 36 290 * |
| ENSG00000072133 | ENSP00000262752 | 745 | RSK4_Mm_AGC_RSK | Pkinase, 73 330 * Pkinase_C, 350 394 * Pkinase, 426 683 * |
| ENSG00000072195 | ENSP00000265327 | 3224 | SPEG_Mm_CAMK_Trio | I-set, 43 125 * ig, 57 110 * V-set, 720 812 * I-set, 722 811 * ig, 736 796 * V-set, 859 960 * I-set, 869 959 * ig, 883 944 * V-set, 967 1059 * I-set, 968 1058 * ig, 982 1043 * V-set, 1062 1154 * I-set, 1064 1153 * ig, 1078 1138 * I-set, 1188 1277 * ig, 1202 1262 * fn3, 1282 1371 * I-set, 1390 1481 * I-set, 1485 1574 * ig, 1499 1559 * Pkinase, 1601 1854 * I-set, 2584 2674 * ig, 2598 2659 * fn3, 2678 2760 * Pkinase, 2966 3217 * |
| ENSG00000072195 | ENSP00000311684 | 3267 | SPEG_Mm_CAMK_Trio | I-set, 43 125 * ig, 57 110 * V-set, 720 812 * I-set, 722 811 * ig, 736 796 * V-set, 859 960 * I-set, 869 959 * ig, 883 944 * V-set, 967 1059 * I-set, 968 1058 * ig, 982 1043 * V-set, 1062 1154 * I-set, 1064 1153 * ig, 1078 1138 * I-set, 1188 1277 * ig, 1202 1262 * fn3, 1282 1371 * I-set, 1390 1481 * I-set, 1485 1574 * ig, 1499 1559 * Pkinase, 1601 1854 * I-set, 2584 2674 * ig, 2598 2659 * fn3, 2678 2760 * Pkinase, 2966 3218 * |
| ENSG00000072195 | ENSP00000387344 | 2475 | SPEG_Mm_CAMK_Trio | V-set, 67 168 * I-set, 77 167 * ig, 91 152 * V-set, 175 267 * I-set, 176 266 * ig, 190 251 * V-set, 270 362 * I-set, 272 361 * ig, 286 346 * I-set, 396 485 * ig, 410 470 * fn3, 490 579 * I-set, 598 689 * I-set, 693 782 * ig, 707 767 * Pkinase, 809 1062 * I-set, 1792 1882 * ig, 1806 1867 * fn3, 1886 1968 * Pkinase, 2174 2426 * |
| ENSG00000072518 | ENSP00000294247 | 753 | MARK1_Hs_CAMK_CAMKL | Pkinase, 82 333 * UBA, 353 389 * KA1, 707 753 * |
| ENSG00000072518 | ENSP00000355091 | 701 | MARK1_Hs_CAMK_CAMKL | Pkinase, 20 271 * UBA, 291 327 * KA1, 655 701 * |
| ENSG00000072518 | ENSP00000367040 | 740 | MARK1_Hs_CAMK_CAMKL | Pkinase, 20 271 * UBA, 291 327 * KA1, 694 740 * |
| ENSG00000072518 | ENSP00000367041 | 691 | MARK1_Hs_CAMK_CAMKL | Pkinase, 20 271 * UBA, 291 327 * KA1, 645 691 * |
| ENSG00000072518 | ENSP00000385751 | 755 | MARK1_Hs_CAMK_CAMKL | Pkinase, 20 271 * UBA, 291 327 * KA1, 709 755 * |
| ENSG00000072518 | ENSP00000386128 | 745 | MARK1_Hs_CAMK_CAMKL | Pkinase, 20 271 * UBA, 291 327 * KA1, 699 745 * |
| ENSG00000072786 | ENSP00000176763 | 968 | LOK_Mm_STE_STE20 | Pkinase, 36 294 * |
| ENSG00000073803 | ENSP00000265026 | 966 | LZK_Sp_TKL_MLK | Pkinase, 168 407 * |
| ENSG00000074590 | ENSP00000261402 | 661 | NuaK1_Mm_CAMK_CAMKL | Pkinase, 55 306 * |
| ENSG00000074966 | ENSP00000264316 | 527 | TXK_Hs_TK_Tec | SH3_1, 85 140 * SH2, 150 231 * Pkinase, 271 520 * |
| ENSG00000075413 | ENSP00000216288 | 744 | MARK1_Hs_CAMK_CAMKL | Pkinase, 56 307 * UBA, 327 363 * KA1, 698 744 * |
| ENSG00000075413 | ENSP00000303698 | 729 | MARK1_Hs_CAMK_CAMKL | Pkinase, 56 307 * UBA, 327 363 * KA1, 683 729 * |
| ENSG00000075413 | ENSP00000335347 | 776 | MARK3_Hs_CAMK_CAMKL | Pkinase, 56 330 * UBA, 350 386 * KA1, 730 776 * |
| ENSG00000076984 | ENSP00000381066 | 419 | MAP2K7_Hs_STE_STE7 | Pkinase, 120 380 * |
| ENSG00000076984 | ENSP00000381068 | 426 | MAP2K7_Hs_STE_STE7 | Pkinase, 120 387 * |
| ENSG00000076984 | ENSP00000381070 | 435 | MAP2K7_Hs_STE_STE7 | Pkinase, 136 396 * |
| ENSG00000077264 | ENSP00000353864 | 580 | PAK3_Mm_STE_STE20 | PBD, 69 163 * Pkinase, 304 555 * APH, 306 482 * |
| ENSG00000077264 | ENSP00000361077 | 544 | PAK3_Mm_STE_STE20 | PBD, 69 127 * Pkinase, 268 519 * APH, 270 446 * |
| ENSG00000077264 | ENSP00000361080 | 559 | PAK3_Mm_STE_STE20 | PBD, 69 142 * Pkinase, 283 534 * APH, 285 461 * |
| ENSG00000077782 | ENSP00000327229 | 820 | FGFR2_Mm_TK_FGFR | I-set, 34 118 * V-set, 35 119 * ig, 48 103 * V-set, 158 248 * I-set, 159 247 * ig, 171 232 * I-set, 255 358 * V-set, 255 359 * ig, 270 343 * Pkinase, 476 752 * |
| ENSG00000077782 | ENSP00000337247 | 812 | FGFR2_Mm_TK_FGFR | I-set, 26 110 * V-set, 27 111 * ig, 40 95 * V-set, 150 240 * I-set, 151 239 * ig, 163 224 * I-set, 247 350 * V-set, 247 351 * ig, 262 335 * Pkinase, 468 744 * |
| ENSG00000077782 | ENSP00000340636 | 822 | FGFR2_Mm_TK_FGFR | I-set, 34 118 * V-set, 35 119 * ig, 48 103 * V-set, 158 248 * I-set, 159 247 * ig, 171 232 * I-set, 255 358 * V-set, 255 359 * ig, 270 343 * Pkinase, 478 754 * |
| ENSG00000077782 | ENSP00000348537 | 733 | FGFR2_Mm_TK_FGFR | V-set, 69 159 * I-set, 70 158 * ig, 82 143 * I-set, 166 269 * V-set, 166 270 * ig, 181 254 * Pkinase, 389 665 * |
| ENSG00000077782 | ENSP00000380292 | 733 | FGFR2_Mm_TK_FGFR | V-set, 67 157 * I-set, 68 156 * ig, 80 141 * I-set, 164 265 * V-set, 164 266 * ig, 179 250 * Pkinase, 389 665 * |
| ENSG00000077782 | ENSP00000380297 | 731 | FGFR2_Mm_TK_FGFR | V-set, 67 157 * I-set, 68 156 * ig, 80 141 * I-set, 164 267 * V-set, 164 268 * ig, 179 252 * Pkinase, 387 663 * |
| ENSG00000077782 | ENSP00000380302 | 820 | FGFR2_Mm_TK_FGFR | I-set, 34 118 * V-set, 35 119 * ig, 48 103 * V-set, 156 246 * I-set, 157 245 * ig, 169 230 * I-set, 253 356 * V-set, 253 357 * ig, 268 341 * Pkinase, 476 752 * |
| ENSG00000078061 | ENSP00000290277 | 609 | ARAF_Mm_TKL_RAF | RBD, 19 91 * C1_1, 99 147 * Pkinase, 313 570 * |
| ENSG00000078061 | ENSP00000366244 | 606 | ARAF_Mm_TKL_RAF | RBD, 19 91 * C1_1, 99 147 * Pkinase, 310 567 * |
| ENSG00000079277 | ENSP00000339573 | 347 | MNK1_Hs_CAMK_MAPKAPK | Pkinase, 49 333 * |
| ENSG00000079277 | ENSP00000361012 | 329 | MNK1_Hs_CAMK_MAPKAPK | Pkinase, 1 238 * |
| ENSG00000079277 | ENSP00000361013 | 424 | MNK1_Hs_CAMK_MAPKAPK | Pkinase, 49 333 * |
| ENSG00000079277 | ENSP00000361014 | 465 | MNK1_Hs_CAMK_MAPKAPK | Pkinase, 49 374 * |
| ENSG00000080224 | ENSP00000374323 | 1036 | EphA6_Hs_TK_Eph | Ephrin_lbd, 34 207 * GCC2_GCC3, 268 312 * fn3, 332 425 * fn3, 440 527 * Pkinase, 631 930 * SAM_2, 958 1025 * SAM_1, 959 1023 * |
| ENSG00000080823 | ENSP00000355304 | 419 | MOK_Mm_CMGC_RCK | Pkinase, 4 285 * |
| ENSG00000080823 | ENSP00000376529 | 419 | MOK_Mm_CMGC_RCK | Pkinase, 4 285 * |
| ENSG00000081320 | ENSP00000263955 | 372 | DRAK2_Hs_CAMK_DAPK | Pkinase, 33 293 * |
| ENSG00000082701 | ENSP00000264235 | 420 | GSK3A_Hs_CMGC_GSK | Pkinase, 56 340 * |
| ENSG00000082701 | ENSP00000324806 | 433 | GSK3A_Hs_CMGC_GSK | Pkinase, 56 353 * |
| ENSG00000083290 | ENSP00000354877 | 1036 | ULK2_Mm_Other_ULK | Pkinase, 9 271 * |
| ENSG00000083290 | ENSP00000378915 | 1036 | ULK2_Mm_Other_ULK | Pkinase, 9 271 * |
| ENSG00000085511 | ENSP00000297332 | 1558 | MAP3K4_Mm_STE_STE11 | Pkinase, 1293 1551 * |
| ENSG00000085511 | ENSP00000355886 | 1498 | MAP3K4_Mm_STE_STE11 | Pkinase, 1293 1498 * |
| ENSG00000085511 | ENSP00000355887 | 1544 | MAP3K4_Mm_STE_STE11 | Pkinase, 1339 1544 * |
| ENSG00000085511 | ENSP00000375985 | 1558 | MAP3K4_Mm_STE_STE11 | Pkinase, 1293 1551 * |
| ENSG00000085511 | ENSP00000375986 | 1608 | MAP3K4_Mm_STE_STE11 | Pkinase, 1343 1601 * |
| ENSG00000086015 | ENSP00000354671 | 1798 | MAST2_Mm_AGC_MAST | DUF1908, 199 476 * Pkinase, 512 785 * Pkinase_C, 803 848 * PDZ, 1104 1189 * |
| ENSG00000086015 | ENSP00000361078 | 792 | MAST2_Mm_AGC_MAST | DUF1908, 84 361 * Pkinase, 397 670 * Pkinase_C, 688 733 * |
| ENSG00000086015 | ENSP00000361079 | 1608 | MAST2_Mm_AGC_MAST | DUF1908, 199 406 * Pkinase, 442 715 * Pkinase_C, 733 778 * PDZ, 1009 1096 * |
| ENSG00000086232 | ENSP00000199389 | 630 | HRI_Hs_Other_PEK | Pkinase, 167 583 * |
| ENSG00000087095 | ENSP00000384625 | 527 | NLK_Hs_CMGC_MAPK | Pkinase, 138 427 * |
| ENSG00000087586 | ENSP00000379243 | 347 | AurC_Mm_Other_AUR | Pkinase, 133 344 * |
| ENSG00000087586 | ENSP00000379245 | 403 | AurC_Mm_Other_AUR | Pkinase, 133 383 * |
| ENSG00000089022 | ENSP00000202788 | 467 | MAPKAPK5_Hs_CAMK_MAPKAPK | Pkinase, 16 298 * |
| ENSG00000091436 | ENSP00000340257 | 455 | ZAK_Mm_TKL_MLK | Pkinase, 16 260 * |
| ENSG00000091436 | ENSP00000364361 | 800 | ZAK_Mm_TKL_MLK | Pkinase, 16 260 * SAM_2, 336 410 * SAM_1, 337 408 * |
| ENSG00000091436 | ENSP00000387259 | 800 | ZAK_Mm_TKL_MLK | Pkinase, 16 260 * SAM_2, 336 410 * SAM_1, 337 408 * |
| ENSG00000092445 | ENSP00000263798 | 890 | TYRO3_Mm_TK_Axl | V-set, 42 136 * I-set, 43 135 * ig, 57 119 * ig, 153 205 * fn3, 225 310 * fn3, 322 406 * Pkinase, 518 786 * |
| ENSG00000095015 | ENSP00000382423 | 1512 | MAP3K1_Hs_STE_STE11 | SWIM, 338 366 * Pkinase, 1243 1508 * |
| ENSG00000095777 | ENSP00000265944 | 1616 | MYO3A_Mm_STE_STE20 | Pkinase, 21 287 * Myosin_head, 340 1041 * IQ, 1056 1076 * IQ, 1083 1103 * IQ, 1347 1367 * |
| ENSG00000095777 | ENSP00000365478 | 197 | 7147_Tt_STE_STE11 | Pkinase, 21 197 * |
| ENSG00000095777 | ENSP00000365479 | 247 | MYO3A_Mm_STE_STE20 | Pkinase, 21 246 * |
| ENSG00000095777 | ENSP00000379679 | 664 | MYO3A_Mm_STE_STE20 | Pkinase, 21 287 * Myosin_head, 340 663 * |
| ENSG00000096063 | ENSP00000339450 | 825 | SRPK1_Hs_CMGC_SRPK | Pkinase, 250 823 * |
| ENSG00000096063 | ENSP00000354674 | 654 | SRPK1_Hs_CMGC_SRPK | Pkinase, 80 652 * |
| ENSG00000096063 | ENSP00000362928 | 547 | SRPK1_Hs_CMGC_SRPK | Pkinase, 2 545 * |
| ENSG00000096063 | ENSP00000362931 | 654 | SRPK1_Hs_CMGC_SRPK | Pkinase, 80 652 * |
| ENSG00000097007 | ENSP00000323315 | 1130 | ABL_Mm_TK_Abl | SH3_1, 64 119 * SH3_2, 65 119 * SH2, 127 202 * Pkinase, 242 493 * F_actin_bind, 953 1130 * |
| ENSG00000097007 | ENSP00000361423 | 1149 | ABL_Mm_TK_Abl | SH3_1, 83 138 * SH3_2, 84 138 * SH2, 146 221 * Pkinase, 261 512 * F_actin_bind, 972 1149 * |
| ENSG00000097046 | ENSP00000234626 | 574 | CDC7_Hs_Other_CDC7 | Pkinase, 58 569 * |
| ENSG00000099308 | ENSP00000262811 | 1309 | MAST3_Mm_AGC_MAST | DUF1908, 56 332 * Pkinase, 367 640 * APH, 369 552 * PDZ, 950 1035 * |
| ENSG00000099725 | ENSP00000372489 | 277 | Pka-C1_Dm_AGC_PKA | Pkinase, 49 277 * |
| ENSG00000099875 | ENSP00000250896 | 465 | MNK2_Hs_CAMK_MAPKAPK | Pkinase, 83 368 * |
| ENSG00000099875 | ENSP00000309485 | 414 | MNK2_Hs_CAMK_MAPKAPK | Pkinase, 83 368 * |
| ENSG00000100030 | ENSP00000215832 | 360 | ERK2_Mm_CMGC_MAPK | Pkinase, 25 313 * |
| ENSG00000100077 | ENSP00000317578 | 688 | BARK1_Hs_AGC_GRK | RGS, 54 174 * Pkinase, 191 453 * PH, 559 652 * |
| ENSG00000100490 | ENSP00000216378 | 358 | CDKL1_Mm_CMGC_CDKL | Pkinase, 5 288 * |
| ENSG00000100749 | ENSP00000216639 | 396 | VRK1_Mm_CK1_VRK | Pkinase, 37 303 * |
| ENSG00000100784 | ENSP00000261991 | 802 | MSK1_Hs_AGC_RSK | Pkinase, 49 318 * Pkinase_C, 338 381 * Pkinase, 426 687 * |
| ENSG00000101049 | ENSP00000340608 | 427 | SGK2_Mm_AGC_SGK | Pkinase, 95 352 * Pkinase_C, 372 421 * |
| ENSG00000101049 | ENSP00000362168 | 366 | SGK2_Mm_AGC_SGK | Pkinase, 35 291 * Pkinase_C, 311 360 * |
| ENSG00000101049 | ENSP00000362192 | 367 | SGK2_Mm_AGC_SGK | Pkinase, 35 292 * Pkinase_C, 312 361 * |
| ENSG00000101109 | ENSP00000361887 | 462 | MST1_Mm_STE_STE20 | Pkinase, 30 281 * |
| ENSG00000101109 | ENSP00000361892 | 487 | MST1_Mm_STE_STE20 | Pkinase, 30 281 * |
| ENSG00000101213 | ENSP00000217185 | 451 | BRK_Hs_TK_Src | SH3_1, 11 70 * SH3_2, 12 70 * SH2, 78 155 * Pkinase, 191 441 * |
| ENSG00000101255 | ENSP00000217233 | 358 | Trb3_Hs_CAMK_Trbl | Pkinase, 71 315 * |
| ENSG00000101266 | ENSP00000339247 | 391 | CK2a2_Mm_CMGC_CK2 | Pkinase, 39 324 * APH, 41 222 * |
| ENSG00000101266 | ENSP00000371400 | 395 | CK2a2_Mm_CMGC_CK2 | Pkinase, 39 324 * APH, 41 222 * |
| ENSG00000101266 | ENSP00000383076 | 255 | 27541_Tt_CMGC_MAPK | Pkinase, 1 188 * |
| ENSG00000101266 | ENSP00000383086 | 385 | CK2a2_Mm_CMGC_CK2 | Pkinase, 39 324 * APH, 41 222 * |
| ENSG00000101306 | ENSP00000365152 | 596 | skMLCK_Hs_CAMK_MLCK | Pkinase, 285 540 * |
| ENSG00000101336 | ENSP00000262651 | 526 | HCK_Hs_TK_Src | SH3_1, 81 136 * SH3_2, 82 136 * SH2, 144 226 * Pkinase, 262 514 * |
| ENSG00000101336 | ENSP00000365012 | 505 | HCK_Hs_TK_Src | SH3_1, 60 115 * SH3_2, 61 115 * SH2, 123 205 * Pkinase, 241 493 * |
| ENSG00000101336 | ENSP00000365022 | 504 | HCK_Hs_TK_Src | SH3_1, 59 114 * SH3_2, 60 114 * SH2, 122 204 * Pkinase, 240 492 * |
| ENSG00000101349 | ENSP00000367679 | 719 | PAK5_Hs_STE_STE20 | PBD, 10 67 * Pkinase, 449 700 * |
| ENSG00000102010 | ENSP00000340082 | 675 | BMX_Mm_TK_Tec | PH, 5 111 * BTK, 118 149 * SH2, 296 377 * Pkinase, 417 666 * |
| ENSG00000102096 | ENSP00000365692 | 311 | PIM2_Mm_CAMK_PIM | Pkinase, 32 286 * |
| ENSG00000102225 | ENSP00000276052 | 496 | PCTAIRE1_Mm_CMGC_CDK | Pkinase, 165 446 * |
| ENSG00000102572 | ENSP00000365716 | 439 | MST3_Mm_STE_STE20 | Pkinase, 12 262 * |
| ENSG00000102572 | ENSP00000365730 | 443 | MST3_Mm_STE_STE20 | Pkinase, 36 286 * |
| ENSG00000102572 | ENSP00000365737 | 431 | MST3_Mm_STE_STE20 | Pkinase, 24 274 * |
| ENSG00000102572 | ENSP00000380651 | 484 | MST3_Mm_STE_STE20 | Pkinase, 77 327 * |
| ENSG00000102755 | ENSP00000282397 | 1338 | FLT1_Hs_TK_VEGFR | ig, 46 109 * ig, 151 209 * V-set, 231 339 * ig, 245 313 * I-set, 332 426 * V-set, 558 656 * ig, 570 638 * I-set, 661 748 * V-set, 661 749 * ig, 675 733 * Pkinase, 827 1154 * |
| ENSG00000102882 | ENSP00000263025 | 379 | ERK1_Mm_CMGC_MAPK | Pkinase, 42 330 * |
| ENSG00000102882 | ENSP00000327293 | 335 | ERK1_Mm_CMGC_MAPK | Pkinase, 42 286 * |
| ENSG00000102882 | ENSP00000378625 | 357 | ERK1_Mm_CMGC_MAPK | Pkinase, 42 330 * |
| ENSG00000102882 | ENSP00000378626 | 311 | ERK1_Mm_CMGC_MAPK | Pkinase, 13 262 * |
| ENSG00000103653 | ENSP00000220003 | 450 | CSK_Mm_TK_Csk | SH3_1, 12 68 * SH3_2, 13 68 * SH2, 82 156 * Pkinase, 195 440 * |
| ENSG00000104205 | ENSP00000262211 | 496 | SGK3_Mm_AGC_SGK | PX, 11 120 * Pkinase, 162 419 * Pkinase_C, 439 491 * |
| ENSG00000104205 | ENSP00000331816 | 464 | SGK3_Mm_AGC_SGK | PX, 11 120 * Pkinase, 162 387 * Pkinase_C, 407 459 * |
| ENSG00000104312 | ENSP00000220751 | 540 | RIPK2_Mm_TKL_RIPK | Pkinase, 18 290 * CARD, 437 524 * |
| ENSG00000104365 | ENSP00000339151 | 756 | IKKb_Mm_Other_IKK | Pkinase, 15 312 * |
| ENSG00000104365 | ENSP00000369030 | 483 | IKKb_Mm_Other_IKK | Pkinase, 1 263 * |
| ENSG00000104375 | ENSP00000347009 | 491 | MST2_Hs_STE_STE20 | Pkinase, 27 278 * |
| ENSG00000104814 | ENSP00000221409 | 833 | HPK1_Mm_STE_STE20 | Pkinase, 17 274 * CNH, 501 807 * |
| ENSG00000104814 | ENSP00000380066 | 821 | HPK1_Mm_STE_STE20 | Pkinase, 17 274 * CNH, 501 807 * |
| ENSG00000104936 | ENSP00000291270 | 629 | DMPK1_Hs_AGC_DMPK | Pkinase, 71 339 * Pkinase_C, 357 409 * DMPK_coil, 470 530 * |
| ENSG00000104936 | ENSP00000345997 | 631 | DMPK1_Hs_AGC_DMPK | Pkinase, 81 349 * Pkinase_C, 367 414 * DMPK_coil, 475 535 * |
| ENSG00000104936 | ENSP00000314954 | 609 | DMPK1_Hs_AGC_DMPK | Pkinase, 81 349 * Pkinase_C, 367 419 * DMPK_coil, 480 540 * |
| ENSG00000104936 | ENSP00000346168 | 575 | DMPK1_Hs_AGC_DMPK | Pkinase, 81 349 * Pkinase_C, 367 419 * DMPK_coil, 480 540 * |
| ENSG00000104936 | ENSP00000366979 | 655 | DMPK1_Hs_AGC_DMPK | Pkinase, 97 365 * Pkinase_C, 383 435 * DMPK_coil, 496 556 * |
| ENSG00000105146 | ENSP00000302898 | 309 | AurC_Mm_Other_AUR | Pkinase, 43 293 * |
| ENSG00000105204 | ENSP00000312789 | 629 | DYRK1B_Hs_CMGC_DYRK | Pkinase, 111 431 * |
| ENSG00000105204 | ENSP00000221803 | 601 | DYRK1B_Hs_CMGC_DYRK | Pkinase, 111 403 * |
| ENSG00000105221 | ENSP00000309428 | 438 | AKT2_Mm_AGC_Akt | PH, 6 108 * Pkinase, 152 366 * Pkinase_C, 386 436 * |
| ENSG00000105221 | ENSP00000375892 | 481 | AKT2_Mm_AGC_Akt | PH, 6 108 * Pkinase, 152 409 * Pkinase_C, 429 479 * |
| ENSG00000105287 | ENSP00000291281 | 878 | PKD2_Mm_CAMK_PKD | C1_1, 139 191 * C1_3, 150 181 * C1_1, 265 317 * C1_3, 276 307 * PH, 398 509 * Pkinase, 551 807 * |
| ENSG00000105397 | ENSP00000264818 | 1187 | TYK2_Hs_TK_JakA | Pkinase, 589 866 * Pkinase, 897 1172 * |
| ENSG00000105613 | ENSP00000251472 | 1570 | MAST1_Mm_AGC_MAST | DUF1908, 59 338 * Pkinase, 374 647 * Pkinase_C, 665 710 * PDZ, 967 1052 * |
| ENSG00000105639 | ENSP00000355038 | 1124 | JAK3_Hs_TK_JakA | SH2, 377 457 * Pkinase, 822 1095 * |
| ENSG00000105723 | ENSP00000222330 | 483 | GSK3A_Hs_CMGC_GSK | Pkinase, 119 403 * |
| ENSG00000105723 | ENSP00000381301 | 401 | GSK3A_Hs_CMGC_GSK | Pkinase, 37 321 * |
| ENSG00000105810 | ENSP00000265734 | 326 | CDK6_Mm_CMGC_CDK | Pkinase, 13 300 * |
| ENSG00000105976 | ENSP00000317272 | 1408 | MET_Mm_TK_Met | Sema, 55 500 * PSI, 519 562 * TIG, 563 654 * TIG, 657 738 * TIG, 762 853 * Pkinase, 1096 1355 * |
| ENSG00000105976 | ENSP00000380860 | 1390 | MET_Mm_TK_Met | Sema, 55 500 * PSI, 519 562 * TIG, 563 654 * TIG, 657 738 * TIG, 742 835 * Pkinase, 1078 1337 * |
| ENSG00000106683 | ENSP00000336740 | 647 | LIMK1_Mm_TKL_LISK | LIM, 25 80 * LIM, 84 142 * PDZ, 165 255 * Pkinase, 339 604 * Kdo, 341 514 * |
| ENSG00000106799 | ENSP00000364129 | 426 | ALK4_Hs_TKL_STKR | Activin_recp, 34 114 * Pkinase, 128 415 * |
| ENSG00000106799 | ENSP00000364133 | 503 | ALK4_Hs_TKL_STKR | Activin_recp, 34 114 * TGF_beta_GS, 175 203 * Pkinase, 205 492 * |
| ENSG00000107140 | ENSP00000338127 | 626 | TESK1_Mm_TKL_LISK | Pkinase, 57 311 * |
| ENSG00000107643 | ENSP00000353483 | 427 | JNK3_Hs_CMGC_MAPK | Pkinase, 26 321 * |
| ENSG00000107643 | ENSP00000363289 | 218 | 19912_Tt_CMGC_GSK | Pkinase, 26 217 * |
| ENSG00000107643 | ENSP00000363291 | 427 | JNK3_Hs_CMGC_MAPK | Pkinase, 26 321 * |
| ENSG00000107643 | ENSP00000363294 | 384 | JNK3_Hs_CMGC_MAPK | Pkinase, 26 321 * |
| ENSG00000107643 | ENSP00000363297 | 384 | JNK3_Hs_CMGC_MAPK | Pkinase, 26 321 * |
| ENSG00000107779 | ENSP00000361107 | 532 | BMPR1A_Mm_TKL_STKR | Activin_recp, 59 138 * TGF_beta_GS, 204 232 * Pkinase, 234 521 * |
| ENSG00000107968 | ENSP00000263056 | 467 | DDB0216377_Dd_STE_STE20 | Pkinase, 136 388 * |
| ENSG00000108443 | ENSP00000225577 | 525 | p70S6Kb_Mm_AGC_RSK | Pkinase, 91 352 * Pkinase_C, 372 417 * |
| ENSG00000108443 | ENSP00000384335 | 451 | p70S6Kb_Mm_AGC_RSK | Pkinase, 91 352 * Pkinase_C, 372 417 * |
| ENSG00000108504 | ENSP00000293215 | 305 | CDC2_Sp_CMGC_CDK | Pkinase, 4 286 * |
| ENSG00000108984 | ENSP00000351997 | 334 | MAP2K6_Mm_STE_STE7 | Pkinase, 53 314 * |
| ENSG00000109339 | ENSP00000309857 | 343 | JNK3_Hs_CMGC_MAPK | Pkinase, 1 238 * |
| ENSG00000109339 | ENSP00000352157 | 464 | JNK3_Hs_CMGC_MAPK | Pkinase, 64 359 * |
| ENSG00000109339 | ENSP00000355297 | 422 | JNK3_Hs_CMGC_MAPK | Pkinase, 64 359 * |
| ENSG00000109339 | ENSP00000378586 | 277 | JNK3_Hs_CMGC_MAPK | Pkinase, 1 214 * |
| ENSG00000109339 | ENSP00000378587 | 422 | JNK3_Hs_CMGC_MAPK | Pkinase, 64 359 * |
| ENSG00000109339 | ENSP00000378590 | 277 | JNK3_Hs_CMGC_MAPK | Pkinase, 1 214 * |
| ENSG00000109339 | ENSP00000378592 | 319 | JNK3_Hs_CMGC_MAPK | Pkinase, 1 214 * |
| ENSG00000109339 | ENSP00000378598 | 426 | JNK3_Hs_CMGC_MAPK | Pkinase, 26 321 * |
| ENSG00000110422 | ENSP00000304226 | 1215 | HIPK3_Mm_CMGC_DYRK | Pkinase, 197 525 * |
| ENSG00000110422 | ENSP00000368301 | 1194 | HIPK3_Mm_CMGC_DYRK | Pkinase, 197 525 * |
| ENSG00000110931 | ENSP00000312741 | 588 | CAMKK2_Mm_Other_CAMKK | Pkinase, 165 446 * |
| ENSG00000110931 | ENSP00000336634 | 541 | CAMKK2_Mm_Other_CAMKK | Pkinase, 165 446 * |
| ENSG00000110931 | ENSP00000321230 | 545 | CAMKK2_Mm_Other_CAMKK | Pkinase, 165 444 * |
| ENSG00000110931 | ENSP00000353636 | 498 | CAMKK2_Mm_Other_CAMKK | Pkinase, 165 444 * |
| ENSG00000110931 | ENSP00000376265 | 624 | CAMKK2_Mm_Other_CAMKK | Pkinase, 256 537 * |
| ENSG00000111816 | ENSP00000357615 | 505 | FRK_Hs_TK_Src | SH3_1, 45 108 * SH3_2, 46 108 * SH2, 116 193 * Pkinase, 234 487 * |
| ENSG00000111837 | ENSP00000313021 | 623 | MAK_Mm_CMGC_RCK | Pkinase, 4 284 * |
| ENSG00000112062 | ENSP00000229794 | 360 | p38a_Mm_CMGC_MAPK | Pkinase, 24 308 * |
| ENSG00000112062 | ENSP00000229795 | 360 | p38a_Mm_CMGC_MAPK | Pkinase, 24 308 * |
| ENSG00000112062 | ENSP00000308669 | 307 | p38a_Mm_CMGC_MAPK | Pkinase, 24 271 * |
| ENSG00000112079 | ENSP00000385174 | 465 | NDR1_Hs_AGC_NDR | Pkinase, 89 382 * Pkinase_C, 400 449 * |
| ENSG00000112144 | ENSP00000263043 | 632 | ICK_Mm_CMGC_RCK | Pkinase, 4 284 * |
| ENSG00000112655 | ENSP00000230419 | 1070 | CCK4_Mm_TK_CCK4 | I-set, 32 121 * ig, 46 103 * I-set, 128 217 * V-set, 128 220 * ig, 143 202 * I-set, 225 319 * V-set, 225 316 * ig, 239 303 * I-set, 331 408 * ig, 336 393 * I-set, 412 498 * V-set, 412 499 * ig, 426 483 * I-set, 502 588 * V-set, 507 589 * ig, 517 572 * V-set, 591 682 * I-set, 592 681 * ig, 606 666 * Pkinase, 796 1061 * |
| ENSG00000112655 | ENSP00000324119 | 396 | CCK4_Mm_TK_CCK4 | I-set, 7 84 * ig, 12 69 * Pkinase, 122 387 * |
| ENSG00000112655 | ENSP00000325992 | 1030 | CCK4_Mm_TK_CCK4 | I-set, 32 121 * ig, 46 103 * I-set, 128 217 * V-set, 128 220 * ig, 143 202 * I-set, 225 319 * V-set, 225 316 * ig, 239 303 * I-set, 331 408 * ig, 336 393 * I-set, 412 548 * V-set, 412 499 * ig, 426 532 * V-set, 551 642 * I-set, 552 641 * ig, 566 626 * Pkinase, 756 1021 * |
| ENSG00000112655 | ENSP00000325462 | 940 | CCK4_Mm_TK_CCK4 | I-set, 32 121 * ig, 46 103 * I-set, 128 217 * V-set, 128 220 * ig, 143 202 * I-set, 225 319 * V-set, 225 316 * ig, 239 303 * I-set, 331 408 * ig, 336 393 * ig, 401 442 * V-set, 461 552 * I-set, 462 551 * ig, 476 536 * Pkinase, 666 931 * |
| ENSG00000112655 | ENSP00000326029 | 1014 | CCK4_Mm_TK_CCK4 | I-set, 32 121 * ig, 46 103 * I-set, 128 217 * V-set, 128 220 * ig, 143 202 * I-set, 225 319 * V-set, 225 316 * ig, 239 303 * I-set, 331 408 * ig, 336 393 * I-set, 412 498 * V-set, 412 499 * ig, 426 483 * I-set, 502 588 * V-set, 507 589 * ig, 517 572 * Pkinase, 740 1005 * |
| ENSG00000112739 | ENSP00000337194 | 1007 | PRP4_Sp_CMGC_DYRK | Pkinase, 687 1003 * |
| ENSG00000112739 | ENSP00000369541 | 374 | DYRK4_Sp_CMGC_DYRK | Pkinase, 176 371 * |
| ENSG00000112742 | ENSP00000230510 | 857 | TTK_Mm_Other_TTK | Pkinase, 525 791 * |
| ENSG00000113240 | ENSP00000316948 | 481 | CLK4_Hs_CMGC_CLK | Pkinase, 159 475 * |
| ENSG00000113263 | ENSP00000231189 | 620 | ITK_Mm_TK_Tec | PH, 5 111 * BTK, 118 149 * SH3_1, 174 229 * SH3_2, 175 229 * SH2, 239 323 * Pkinase, 363 612 * |
| ENSG00000113712 | ENSP00000261798 | 365 | C03C10.1_Ce_CK1_CK1 | Pkinase, 17 309 * |
| ENSG00000113712 | ENSP00000367074 | 337 | C03C10.1_Ce_CK1_CK1 | Pkinase, 17 281 * |
| ENSG00000113721 | ENSP00000261799 | 1106 | PDGFRb_Hs_TK_PDGFR | V-set, 32 119 * ig, 47 102 * V-set, 213 311 * I-set, 214 310 * ig, 228 293 * ig, 331 398 * Pkinase, 600 958 * |
| ENSG00000114124 | ENSP00000264952 | 553 | GPRK7_Hs_AGC_GRK | RGS, 55 175 * Pkinase, 191 454 * |
| ENSG00000114670 | ENSP00000264996 | 470 | NEK11_Mm_Other_NEK | Pkinase, 29 287 * |
| ENSG00000114670 | ENSP00000349389 | 482 | NEK11_Mm_Other_NEK | Pkinase, 29 287 * |
| ENSG00000114670 | ENSP00000372857 | 645 | NEK11_Mm_Other_NEK | Pkinase, 29 287 * |
| ENSG00000114738 | ENSP00000350639 | 382 | MAPKAPK3_Mm_CAMK_MAPKAPK | Pkinase, 43 304 * |
| ENSG00000114739 | ENSP00000340361 | 512 | ACTR2B_Mm_TKL_STKR | Activin_recp, 27 117 * Pkinase, 190 478 * |
| ENSG00000114904 | ENSP00000233027 | 841 | NEK4_Mm_Other_NEK | Pkinase, 6 261 * |
| ENSG00000114904 | ENSP00000373227 | 781 | NEK4_Mm_Other_NEK | Pkinase, 6 261 * |
| ENSG00000115085 | ENSP00000264972 | 619 | ZAP70_Mm_TK_Syk | SH2, 10 87 * SH2, 163 239 * Pkinase, 338 593 * |
| ENSG00000115085 | ENSP00000374169 | 312 | ZAP70_Mm_TK_Syk | Pkinase, 31 286 * |
| ENSG00000115170 | ENSP00000263640 | 509 | ALK2_Hs_TKL_STKR | Activin_recp, 33 107 * TGF_beta_GS, 178 206 * Pkinase, 208 495 * |
| ENSG00000115661 | ENSP00000387156 | 350 | MPSK1_Mm_Other_NAK | Pkinase, 65 335 * |
| ENSG00000115661 | ENSP00000386928 | 305 | MPSK1_Mm_Other_NAK | Pkinase, 20 290 * |
| ENSG00000115661 | ENSP00000386553 | 273 | MPSK1_Mm_Other_NAK | Pkinase, 20 258 * |
| ENSG00000115687 | ENSP00000234040 | 1323 | PASK_Mm_CAMK_CAMKL | Pkinase, 999 1251 * |
| ENSG00000115687 | ENSP00000351475 | 1330 | PASK_Mm_CAMK_CAMKL | Pkinase, 999 1258 * |
| ENSG00000115694 | ENSP00000385687 | 426 | YSK1_Mm_STE_STE20 | Pkinase, 20 270 * |
| ENSG00000115694 | ENSP00000384444 | 349 | YSK1_Mm_STE_STE20 | Pkinase, 1 193 * |
| ENSG00000115825 | ENSP00000234179 | 890 | PKD3_Hs_CAMK_PKD | C1_1, 155 207 * C1_3, 166 197 * C1_1, 272 324 * C1_3, 283 314 * PH, 417 532 * Pkinase, 574 832 * |
| ENSG00000115977 | ENSP00000375072 | 863 | AAK1_Mm_Other_NAK | Pkinase, 46 310 * |
| ENSG00000115977 | ENSP00000386342 | 674 | AAK1_Mm_Other_NAK | Pkinase, 46 310 * |
| ENSG00000115977 | ENSP00000386456 | 961 | AAK1_Mm_Other_NAK | Pkinase, 46 310 * |
| ENSG00000116106 | ENSP00000281821 | 986 | EphB4_Mm_TK_Eph | Ephrin_lbd, 30 204 * fn3, 329 420 * fn3, 441 525 * Pkinase, 621 878 * SAM_2, 908 975 * SAM_1, 909 973 * |
| ENSG00000116141 | ENSP00000355884 | 795 | MARK1_Hs_CAMK_CAMKL | Pkinase, 60 311 * UBA, 331 367 * KA1, 749 795 * |
| ENSG00000116141 | ENSP00000355885 | 758 | MARK1_Hs_CAMK_CAMKL | Pkinase, 60 289 * UBA, 309 345 * KA1, 712 758 * |
| ENSG00000116141 | ENSP00000386017 | 780 | MARK1_Hs_CAMK_CAMKL | Pkinase, 60 311 * UBA, 331 367 * KA1, 734 780 * |
| ENSG00000116783 | ENSP00000322251 | 835 | HH498_Hs_TKL_MLK | Ank, 66 99 * Ank, 100 132 * Ank, 133 165 * Ank, 166 198 * Ank, 199 233 * Ank, 234 264 * Ank, 269 302 * Ank, 304 338 * Ank, 339 371 * Ank, 381 413 * Pkinase, 463 719 * |
| ENSG00000116783 | ENSP00000359928 | 936 | HH498_Hs_TKL_MLK | Ank, 167 200 * Ank, 201 233 * Ank, 234 266 * Ank, 267 299 * Ank, 300 334 * Ank, 335 365 * Ank, 370 403 * Ank, 405 439 * Ank, 440 472 * Ank, 482 514 * Pkinase, 564 820 * |
| ENSG00000116783 | ENSP00000359936 | 843 | HH498_Hs_TKL_MLK | Ank, 167 200 * Ank, 201 233 * Ank, 234 266 * Ank, 267 299 * Ank, 300 334 * Ank, 335 365 * Ank, 370 403 * Ank, 405 439 * Ank, 440 472 * Ank, 482 514 * Pkinase, 564 838 * |
| ENSG00000117020 | ENSP00000263826 | 507 | AKT2_Mm_AGC_Akt | PH, 34 135 * Pkinase, 176 433 * Pkinase_C, 453 505 * |
| ENSG00000117020 | ENSP00000336943 | 465 | AKT2_Mm_AGC_Akt | PH, 6 107 * Pkinase, 148 405 * Pkinase_C, 425 464 * |
| ENSG00000117020 | ENSP00000355497 | 479 | AKT2_Mm_AGC_Akt | PH, 6 107 * Pkinase, 148 405 * Pkinase_C, 425 477 * |
| ENSG00000117020 | ENSP00000375720 | 493 | AKT2_Mm_AGC_Akt | PH, 34 135 * Pkinase, 176 433 * Pkinase_C, 453 492 * |
| ENSG00000117266 | ENSP00000342589 | 504 | PCTAIRE3_Mm_CMGC_CDK | Pkinase, 174 455 * |
| ENSG00000117266 | ENSP00000353176 | 472 | PCTAIRE3_Mm_CMGC_CDK | Pkinase, 142 423 * |
| ENSG00000117650 | ENSP00000355965 | 384 | NEK2_Mm_Other_NEK | Pkinase, 8 271 * |
| ENSG00000117650 | ENSP00000355966 | 445 | NEK2_Mm_Other_NEK | Pkinase, 8 271 * |
| ENSG00000117676 | ENSP00000363277 | 744 | RSK3_Mm_AGC_RSK | Pkinase, 71 328 * Pkinase_C, 350 394 * Pkinase, 427 684 * |
| ENSG00000117676 | ENSP00000363278 | 578 | RSK3~b_Mm_AGC_RSK | Pkinase, 1 162 * Pkinase_C, 184 228 * Pkinase, 261 518 * |
| ENSG00000117676 | ENSP00000363283 | 735 | RSK3_Mm_AGC_RSK | Pkinase, 62 319 * Pkinase_C, 341 385 * Pkinase, 418 675 * |
| ENSG00000118046 | ENSP00000384275 | 433 | LKB1_Mm_CAMK_CAMKL | Pkinase, 49 309 * |
| ENSG00000118515 | ENSP00000237305 | 431 | SGK1_Mm_AGC_SGK | Pkinase, 98 355 * Pkinase_C, 375 427 * |
| ENSG00000118515 | ENSP00000356829 | 421 | SGK1_Mm_AGC_SGK | Pkinase, 88 345 * Pkinase_C, 365 417 * |
| ENSG00000118515 | ENSP00000356831 | 445 | SGK1_Mm_AGC_SGK | Pkinase, 112 369 * Pkinase_C, 389 441 * |
| ENSG00000118515 | ENSP00000356832 | 526 | SGK1_Mm_AGC_SGK | Pkinase, 193 450 * Pkinase_C, 470 522 * |
| ENSG00000119408 | ENSP00000319734 | 313 | NEK6_Hs_Other_NEK | Pkinase, 45 306 * |
| ENSG00000119408 | ENSP00000362703 | 245 | NEK6_Hs_Other_NEK | Pkinase, 15 238 * |
| ENSG00000119408 | ENSP00000377749 | 347 | NEK6_Hs_Other_NEK | Pkinase, 79 340 * |
| ENSG00000119638 | ENSP00000238616 | 979 | NEK9_Mm_Other_NEK | Pkinase, 52 308 * RCC1, 387 441 * RCC1, 444 495 * RCC1, 500 547 * RCC1, 615 665 * |
| ENSG00000120156 | ENSP00000343716 | 1124 | TIE2_Mm_TK_Tie | Ig_Tie2_1, 23 118 * Laminin_EGF, 219 268 * EGF, 224 251 * EGF_2, 224 251 * EGF, 268 298 * EGF_2, 315 340 * fn3, 444 529 * fn3, 543 626 * fn3, 639 724 * Pkinase, 824 1092 * |
| ENSG00000120156 | ENSP00000369375 | 1081 | TIE2_Mm_TK_Tie | Ig_Tie2_1, 23 118 * Laminin_EGF, 219 268 * EGF, 224 251 * EGF_2, 224 251 * EGF, 268 298 * fn3, 401 486 * fn3, 500 583 * fn3, 596 681 * Pkinase, 781 1049 * |
| ENSG00000120156 | ENSP00000383977 | 1157 | TIE2_Mm_TK_Tie | Ig_Tie2_1, 56 151 * Laminin_EGF, 252 301 * EGF, 257 284 * EGF_2, 257 284 * EGF, 301 331 * EGF_2, 348 373 * fn3, 477 562 * fn3, 576 659 * fn3, 672 757 * Pkinase, 857 1125 * |
| ENSG00000120539 | ENSP00000343446 | 840 | MASTL_Hs_AGC_MAST | Pkinase, 35 291 * APH, 37 214 * |
| ENSG00000120539 | ENSP00000365107 | 879 | MASTL_Hs_AGC_MAST | Pkinase, 35 835 * APH, 37 214 * |
| ENSG00000120539 | ENSP00000365113 | 878 | MASTL_Hs_AGC_MAST | Pkinase, 35 834 * APH, 37 214 * |
| ENSG00000120899 | ENSP00000342242 | 967 | PYK2_Mm_TK_FAK | FERM_M, 143 265 * Pkinase, 425 679 * Focal_AT, 828 966 * |
| ENSG00000120899 | ENSP00000332816 | 1009 | PYK2_Mm_TK_FAK | FERM_M, 143 265 * Pkinase, 425 679 * Focal_AT, 870 1008 * |
| ENSG00000120899 | ENSP00000380634 | 596 | PYK2_Mm_TK_FAK | Pkinase, 171 425 * |
| ENSG00000121989 | ENSP00000241416 | 513 | ACTR2_Hs_TKL_STKR | Activin_recp, 28 118 * Pkinase, 192 479 * |
| ENSG00000121989 | ENSP00000385568 | 543 | ACTR2_Hs_TKL_STKR | Activin_recp, 58 148 * Pkinase, 222 509 * |
| ENSG00000122025 | ENSP00000241453 | 993 | FLT3_Hs_TK_PDGFR | ig, 265 332 * Pkinase, 610 943 * |
| ENSG00000122025 | ENSP00000370369 | 985 | FLT3_Hs_TK_PDGFR | ig, 254 321 * Pkinase, 599 935 * |
| ENSG00000122025 | ENSP00000370374 | 996 | FLT3_Hs_TK_PDGFR | ig, 265 332 * Pkinase, 610 946 * |
| ENSG00000122966 | ENSP00000261833 | 2027 | CRIK_Hs_AGC_DMPK | Pkinase, 97 360 * Pkinase_C, 378 425 * M, 664 684 * Filament, 761 1074 * M, 821 841 * M, 906 926 * M, 944 964 * C1_1, 1363 1414 * PH, 1444 1563 * CNH, 1593 1883 * |
| ENSG00000122966 | ENSP00000376306 | 2069 | CRIK_Hs_AGC_DMPK | Pkinase, 97 360 * Pkinase_C, 378 425 * M, 664 684 * Filament, 803 1116 * M, 863 883 * M, 948 968 * M, 986 1006 * C1_1, 1405 1456 * PH, 1486 1605 * CNH, 1635 1925 * |
| ENSG00000123143 | ENSP00000242783 | 942 | PKN1_Mm_AGC_PKN | HR1, 37 110 * HR1, 126 203 * HR1, 213 290 * Pkinase, 615 874 * Pkinase_C, 894 941 * |
| ENSG00000123143 | ENSP00000343325 | 948 | PKN1_Mm_AGC_PKN | HR1, 43 116 * HR1, 132 209 * HR1, 219 296 * Pkinase, 621 880 * Pkinase_C, 900 947 * |
| ENSG00000123374 | ENSP00000266970 | 298 | CDK2_Hs_CMGC_CDK | Pkinase, 4 286 * |
| ENSG00000123374 | ENSP00000243067 | 264 | CDK2_Hs_CMGC_CDK | Pkinase, 4 252 * |
| ENSG00000123572 | ENSP00000243300 | 1583 | ZC4_Mm_STE_STE20 | Pkinase, 25 313 * CNH, 1210 1557 * |
| ENSG00000123612 | ENSP00000243349 | 493 | ALK7_Mm_TKL_STKR | Activin_recp, 26 100 * TGF_beta_GS, 165 193 * Pkinase, 195 482 * |
| ENSG00000123612 | ENSP00000335178 | 413 | ALK7_Mm_TKL_STKR | Activin_recp, 26 100 * Pkinase, 115 402 * |
| ENSG00000123612 | ENSP00000335139 | 336 | ALK7_Mm_TKL_STKR | Activin_recp, 26 100 * Pkinase, 38 325 * |
| ENSG00000123612 | ENSP00000387168 | 443 | ALK7_Mm_TKL_STKR | TGF_beta_GS, 115 143 * Pkinase, 145 432 * |
| ENSG00000125388 | ENSP00000264764 | 685 | GPRK5_Hs_AGC_GRK | RGS, 205 324 * Pkinase, 340 602 * |
| ENSG00000125388 | ENSP00000381125 | 545 | GPRK6_Hs_AGC_GRK | RGS, 19 138 * Pkinase, 154 416 * |
| ENSG00000125388 | ENSP00000381128 | 532 | GPRK5_Hs_AGC_GRK | RGS, 52 171 * Pkinase, 187 449 * |
| ENSG00000125388 | ENSP00000381129 | 578 | GPRK6_Hs_AGC_GRK | RGS, 52 171 * Pkinase, 187 449 * |
| ENSG00000125508 | ENSP00000217188 | 488 | SFK6_Sp_TK_Src | SH3_1, 54 110 * SH3_2, 55 110 * SH2, 120 197 * Pkinase, 230 480 * |
| ENSG00000125834 | ENSP00000370891 | 401 | CLIK1_Hs_Other_NKF4 | Pkinase, 69 394 * |
| ENSG00000126562 | ENSP00000246914 | 1243 | Wnk4_Mm_Other_WNK | Pkinase, 174 432 * |
| ENSG00000126583 | ENSP00000263431 | 697 | PKCg_Mm_AGC_PKC | C1_1, 36 88 * C1_1, 101 153 * C2, 173 260 * Pkinase, 351 614 * Pkinase_C, 634 679 * |
| ENSG00000126934 | ENSP00000262948 | 400 | MAP2K2_Hs_STE_STE7 | Pkinase, 72 369 * |
| ENSG00000126934 | ENSP00000378336 | 303 | MAP2K2_Hs_STE_STE7 | Pkinase, 1 272 * |
| ENSG00000127334 | ENSP00000342105 | 601 | DYRK2_Hs_CMGC_DYRK | Pkinase, 222 535 * |
| ENSG00000127334 | ENSP00000377186 | 528 | DYRK2_Hs_CMGC_DYRK | Pkinase, 149 462 * |
| ENSG00000127564 | ENSP00000371675 | 480 | MYT1_Mm_Other_WEE | Pkinase, 110 359 * |
| ENSG00000127564 | ENSP00000384251 | 499 | MYT1_Mm_Other_WEE | Pkinase, 110 359 * |
| ENSG00000128052 | ENSP00000263923 | 1356 | KDR_Mm_TK_VEGFR | ig, 46 105 * V-set, 225 325 * ig, 239 309 * I-set, 330 417 * V-set, 333 418 * ig, 345 400 * ig, 564 644 * V-set, 666 766 * I-set, 667 754 * ig, 681 739 * Pkinase, 834 1160 * |
| ENSG00000128829 | ENSP00000263791 | 1649 | GCN2_Hs_Other_PEK | RWD, 18 134 * Pkinase, 315 539 * Pkinase, 590 1001 * tRNA-synt_2b, 1063 1223 * |
| ENSG00000128829 | ENSP00000372174 | 1621 | GCN2_Hs_Other_PEK | RWD, 18 134 * Pkinase, 315 539 * Pkinase, 590 973 * tRNA-synt_2b, 1035 1195 * |
| ENSG00000128881 | ENSP00000263802 | 1649 | TTBK2_Mm_CK1_TTBK | Pkinase, 13 277 * Filament, 342 591 * |
| ENSG00000128881 | ENSP00000267890 | 1244 | TTBK2_Mm_CK1_TTBK | Pkinase, 21 279 * |
| ENSG00000128881 | ENSP00000382403 | 1174 | TTBK2_Mm_CK1_TTBK | Pkinase, 1 209 * |
| ENSG00000129465 | ENSP00000216274 | 518 | RIPK3_Hs_TKL_RIPK | Pkinase, 21 287 * |
| ENSG00000129465 | ENSP00000371997 | 426 | RIPK3_Hs_TKL_RIPK | Pkinase, 21 287 * |
| ENSG00000130413 | ENSP00000320754 | 514 | DDB0216308_Dd_CAMK_CAMK1 | Pkinase, 116 381 * |
| ENSG00000130413 | ENSP00000351743 | 327 | 23956_Tt_Other_Ciliate-E2 | Pkinase, 1 194 * |
| ENSG00000130413 | ENSP00000379906 | 448 | DDB0216308_Dd_CAMK_CAMK1 | Pkinase, 116 381 * |
| ENSG00000130669 | ENSP00000326864 | 438 | PAK4_Mm_STE_STE20 | PBD, 10 67 * Pkinase, 168 419 * |
| ENSG00000130669 | ENSP00000353625 | 591 | PAK4_Mm_STE_STE20 | PBD, 10 67 * Pkinase, 321 572 * |
| ENSG00000130758 | ENSP00000253055 | 954 | MLK2_Mm_TKL_MLK | SH3_1, 19 79 * SH3_2, 20 79 * Pkinase, 98 357 * |
| ENSG00000130822 | ENSP00000359164 | 366 | CaMK1b_Hs_CAMK_CAMK1 | Pkinase, 15 293 * |
| ENSG00000130822 | ENSP00000359169 | 343 | CaMK1b_Hs_CAMK_CAMK1 | Pkinase, 15 270 * |
| ENSG00000130822 | ENSP00000377417 | 395 | CaMK1b_Hs_CAMK_CAMK1 | Pkinase, 43 322 * |
| ENSG00000131023 | ENSP00000253339 | 1130 | LATS1_Hs_AGC_NDR | UBA, 101 138 * Pkinase, 705 1010 * Pkinase_C, 1029 1084 * |
| ENSG00000132155 | ENSP00000251849 | 648 | RAF1_Hs_TKL_RAF | RBD, 56 131 * C1_1, 139 187 * Pkinase, 349 606 * |
| ENSG00000132356 | ENSP00000296800 | 207 | PASK_Hs_CAMK_CAMKL | Pkinase, 18 203 * |
| ENSG00000132356 | ENSP00000346148 | 574 | AMPKa1_Mm_CAMK_CAMKL | Pkinase, 27 294 * |
| ENSG00000132356 | ENSP00000380317 | 559 | AMPKa1_Mm_CAMK_CAMKL | Pkinase, 27 279 * |
| ENSG00000132964 | ENSP00000370938 | 464 | CDK8_Mm_CMGC_CDK | Pkinase, 20 335 * |
| ENSG00000133059 | ENSP00000356129 | 884 | SgK496_Hs_Other_SgK496 | Pkinase, 652 864 * |
| ENSG00000133059 | ENSP00000356130 | 929 | SgK496_Hs_Other_SgK496 | Pkinase, 652 909 * |
| ENSG00000133083 | ENSP00000255448 | 729 | DCAMKL1_Mm_CAMK_DCAMKL | DCX, 74 138 * DCX, 203 264 * Pkinase, 390 647 * |
| ENSG00000133083 | ENSP00000353846 | 740 | DCAMKL1_Mm_CAMK_DCAMKL | DCX, 74 138 * DCX, 203 264 * Pkinase, 390 647 * |
| ENSG00000133083 | ENSP00000369223 | 433 | DCAMKL1_Mm_CAMK_DCAMKL | Pkinase, 83 340 * |
| ENSG00000133083 | ENSP00000382257 | 421 | DCAMKL1_Mm_CAMK_DCAMKL | Pkinase, 82 339 * |
| ENSG00000133216 | ENSP00000363755 | 981 | EphB4_Mm_TK_Eph | Ephrin_lbd, 14 191 * GCC2_GCC3, 253 297 * fn3, 319 415 * fn3, 430 515 * Pkinase, 616 875 * SAM_2, 905 972 * SAM_1, 906 970 * |
| ENSG00000133216 | ENSP00000363758 | 946 | EphB4_Mm_TK_Eph | Ephrin_lbd, 14 191 * GCC2_GCC3, 253 297 * fn3, 319 415 * fn3, 430 515 * Pkinase, 616 875 * SAM_1, 906 946 * |
| ENSG00000133216 | ENSP00000363761 | 1055 | EphB4_Mm_TK_Eph | Ephrin_lbd, 20 197 * GCC2_GCC3, 259 303 * fn3, 325 421 * fn3, 436 520 * Pkinase, 621 880 * SAM_2, 910 977 * SAM_1, 911 975 * |
| ENSG00000133216 | ENSP00000363763 | 987 | EphB4_Mm_TK_Eph | Ephrin_lbd, 20 197 * GCC2_GCC3, 259 303 * fn3, 325 421 * fn3, 436 520 * Pkinase, 622 881 * SAM_2, 911 978 * SAM_1, 912 976 * |
| ENSG00000133216 | ENSP00000383053 | 986 | EphB4_Mm_TK_Eph | Ephrin_lbd, 20 197 * GCC2_GCC3, 259 303 * fn3, 325 421 * fn3, 436 520 * Pkinase, 621 880 * SAM_2, 910 977 * SAM_1, 911 975 * |
| ENSG00000133275 | ENSP00000255641 | 415 | gish_Dm_CK1_CK1 | Pkinase, 46 312 * |
| ENSG00000134058 | ENSP00000256443 | 346 | CDK7_Mm_CMGC_CDK | Pkinase, 12 295 * |
| ENSG00000134072 | ENSP00000380447 | 370 | CaMK1a_Hs_CAMK_CAMK1 | Pkinase, 20 276 * |
| ENSG00000134318 | ENSP00000317985 | 1428 | ROCK2_Mm_AGC_DMPK | Pkinase, 132 394 * Pkinase_C, 414 459 * M, 501 521 * HR1, 515 599 * M, 564 584 * M, 630 650 * M, 743 763 * M, 813 833 * M, 876 896 * M, 932 952 * M, 1013 1033 * Rho_Binding, 1018 1086 * PH, 1191 1298 * C1_1, 1301 1353 * |
| ENSG00000134318 | ENSP00000382039 | 1388 | ROCK2_Mm_AGC_DMPK | Pkinase, 92 354 * Pkinase_C, 374 419 * M, 461 481 * HR1, 475 559 * M, 524 544 * M, 590 610 * M, 703 723 * M, 773 793 * M, 836 856 * M, 892 912 * M, 973 993 * Rho_Binding, 978 1046 * PH, 1151 1258 * C1_1, 1261 1313 * |
| ENSG00000134398 | ENSP00000256797 | 974 | IRE1_Mm_Other_IRE | Pkinase, 568 829 * Ribonuc_2-5A, 834 960 * |
| ENSG00000134602 | ENSP00000310468 | 354 | MST4_Mm_STE_STE20 | Pkinase, 24 305 * |
| ENSG00000134602 | ENSP00000354757 | 392 | MST4_Mm_STE_STE20 | Pkinase, 24 274 * |
| ENSG00000134602 | ENSP00000359924 | 496 | MST4_Mm_STE_STE20 | Pkinase, 104 354 * |
| ENSG00000134602 | ENSP00000377867 | 416 | MST4_Mm_STE_STE20 | Pkinase, 24 274 * |
| ENSG00000134602 | ENSP00000377868 | 419 | MST4_Mm_STE_STE20 | Pkinase, 25 277 * |
| ENSG00000134853 | ENSP00000257290 | 1089 | PDGFRa_Mm_TK_PDGFR | ig, 42 102 * ig, 228 292 * I-set, 319 413 * ig, 330 396 * Pkinase, 593 953 * |
| ENSG00000135090 | ENSP00000376317 | 898 | TAO3_Mm_STE_STE20 | Pkinase, 24 277 * APH, 26 205 * |
| ENSG00000135250 | ENSP00000337679 | 730 | SRPK2_Hs_CMGC_SRPK | Pkinase, 123 728 * |
| ENSG00000135250 | ENSP00000349863 | 688 | SRPK2_Hs_CMGC_SRPK | Pkinase, 81 686 * |
| ENSG00000135250 | ENSP00000377260 | 688 | SRPK2_Hs_CMGC_SRPK | Pkinase, 81 686 * |
| ENSG00000135250 | ENSP00000377262 | 699 | SRPK2_Hs_CMGC_SRPK | Pkinase, 92 697 * |
| ENSG00000135333 | ENSP00000358309 | 998 | EphB4_Mm_TK_Eph | Ephrin_lbd, 32 205 * NCD3G, 258 302 * GCC2_GCC3, 268 312 * fn3, 332 427 * fn3, 443 527 * Pkinase, 633 890 * SAM_2, 920 987 * SAM_1, 921 985 * |
| ENSG00000135341 | ENSP00000358331 | 518 | TAK1_Hs_TKL_MLK | Pkinase, 36 284 * |
| ENSG00000135341 | ENSP00000358333 | 491 | TAK1_Hs_TKL_MLK | Pkinase, 36 284 * |
| ENSG00000135341 | ENSP00000358335 | 606 | TAK1_Hs_TKL_MLK | Pkinase, 36 284 * |
| ENSG00000135341 | ENSP00000358338 | 579 | TAK1_Hs_TKL_MLK | Pkinase, 36 284 * |
| ENSG00000135409 | ENSP00000257863 | 573 | MISR2_Mm_TKL_STKR | Pkinase, 203 505 * |
| ENSG00000135409 | ENSP00000369117 | 478 | BMPR1_Sp_TKL_STKR | Pkinase, 203 410 * |
| ENSG00000135446 | ENSP00000257904 | 303 | CDK4_Mm_CMGC_CDK | Pkinase, 6 295 * APH, 8 204 * |
| ENSG00000135503 | ENSP00000257963 | 505 | ALK4_Hs_TKL_STKR | Activin_recp, 32 109 * TGF_beta_GS, 177 205 * Pkinase, 207 494 * |
| ENSG00000135605 | ENSP00000370912 | 631 | TEC_Hs_TK_Tec | PH, 5 111 * BTK, 118 149 * SH3_1, 182 237 * SH3_2, 183 234 * SH2, 247 330 * Pkinase, 370 619 * |
| ENSG00000136098 | ENSP00000258597 | 507 | NEK3_Hs_Other_NEK | Pkinase, 4 255 * |
| ENSG00000136098 | ENSP00000339429 | 506 | NEK3_Hs_Other_NEK | Pkinase, 4 255 * |
| ENSG00000136098 | ENSP00000383210 | 489 | NEK3_Hs_Other_NEK | Pkinase, 4 255 * |
| ENSG00000136573 | ENSP00000259089 | 505 | BLK_Hs_TK_Src | SH3_1, 61 116 * SH3_2, 62 116 * SH2, 124 205 * Pkinase, 241 493 * |
| ENSG00000136643 | ENSP00000355926 | 1054 | RSKL1_Hs_AGC_RSKL | PX, 9 116 * MIT, 227 295 * Pkinase, 826 1044 * |
| ENSG00000136643 | ENSP00000355927 | 1066 | RSKL1_Hs_AGC_RSKL | PX, 9 128 * MIT, 239 307 * Pkinase, 838 1056 * |
| ENSG00000136807 | ENSP00000362361 | 372 | CDK9_Mm_CMGC_CDK | Pkinase, 19 315 * |
| ENSG00000136807 | ENSP00000362362 | 489 | CDK9_Mm_CMGC_CDK | Pkinase, 136 432 * |
| ENSG00000137193 | ENSP00000362606 | 404 | PIM1_Mm_CAMK_PIM | Pkinase, 129 381 * |
| ENSG00000137193 | ENSP00000362608 | 313 | PIM1_Mm_CAMK_PIM | Pkinase, 38 290 * |
| ENSG00000137275 | ENSP00000259808 | 671 | RIPK1_Hs_TKL_RIPK | Pkinase, 17 285 * Death, 584 669 * |
| ENSG00000137332 | ENSP00000383262 | 876 | DDR1_Mm_TK_DDR | F5_F8_type_C, 46 182 * Pkinase, 573 876 * |
| ENSG00000137601 | ENSP00000309742 | 1214 | NEK1_Mm_Other_NEK | Pkinase, 4 258 * |
| ENSG00000137601 | ENSP00000336715 | 1258 | NEK1_Mm_Other_NEK | Pkinase, 4 258 * |
| ENSG00000137601 | ENSP00000368363 | 1286 | NEK1_Mm_Other_NEK | Pkinase, 4 258 * |
| ENSG00000137764 | ENSP00000178640 | 448 | MAP2K5_Mm_STE_STE7 | PB1, 18 97 * Pkinase, 166 419 * |
| ENSG00000137764 | ENSP00000342101 | 258 | MAP2K5_Mm_STE_STE7 | Pkinase, 4 229 * |
| ENSG00000137764 | ENSP00000346493 | 442 | MAP2K5_Hs_STE_STE7 | PB1, 18 97 * Pkinase, 166 409 * |
| ENSG00000137764 | ENSP00000378859 | 438 | MAP2K5_Hs_STE_STE7 | PB1, 18 97 * Pkinase, 166 409 * |
| ENSG00000137843 | ENSP00000260404 | 681 | PAK6_Mm_STE_STE20 | PBD, 11 67 * Pkinase, 407 658 * |
| ENSG00000138395 | ENSP00000260967 | 384 | PFTAIRE2_Mm_CMGC_CDK | Pkinase, 52 336 * |
| ENSG00000138395 | ENSP00000363726 | 345 | PFTAIRE2_Mm_CMGC_CDK | Pkinase, 103 325 * |
| ENSG00000138395 | ENSP00000387055 | 378 | PFTAIRE2_Mm_CMGC_CDK | Pkinase, 52 336 * |
| ENSG00000138669 | ENSP00000264399 | 762 | PKG2_Mm_AGC_PKG | cNMP_binding, 186 271 * cNMP_binding, 304 396 * Pkinase, 453 711 * |
| ENSG00000138696 | ENSP00000264568 | 502 | BMPR1B_Mm_TKL_STKR | Activin_recp, 30 110 * TGF_beta_GS, 174 202 * Pkinase, 204 491 * |
| ENSG00000138756 | ENSP00000264889 | 676 | BIKE_Mm_Other_NAK | Pkinase, 51 314 * |
| ENSG00000138756 | ENSP00000373662 | 662 | BIKE_Mm_Other_NAK | Pkinase, 51 314 * |
| ENSG00000138769 | ENSP00000306340 | 493 | CDKL2_Hs_CMGC_CDKL | Pkinase, 4 287 * |
| ENSG00000139567 | ENSP00000373574 | 503 | ALK1_Hs_TKL_STKR | Activin_recp, 32 103 * TGF_beta_GS, 172 200 * Pkinase, 202 489 * |
| ENSG00000139625 | ENSP00000267079 | 859 | LZK_Sp_TKL_MLK | Pkinase, 125 364 * |
| ENSG00000139908 | ENSP00000287913 | 328 | TSSK4_Hs_CAMK_TSSK | Pkinase, 25 293 * |
| ENSG00000139908 | ENSP00000339179 | 338 | TSSK4_Hs_CAMK_TSSK | Pkinase, 25 303 * |
| ENSG00000140443 | ENSP00000268035 | 1367 | IGF1R_Hs_TK_InsR | Recep_L_domain, 51 161 * Furin-like, 175 333 * Recep_L_domain, 352 467 * fn3, 489 587 * fn3, 835 917 * Pkinase, 999 1266 * |
| ENSG00000140474 | ENSP00000268074 | 214 | ULK3_Mm_Other_ULK | Pkinase, 14 212 * |
| ENSG00000140538 | ENSP00000347397 | 825 | TRKC_Mm_TK_Trk | LRRNT, 31 58 * LRR_1, 104 126 * LRR_1, 128 150 * LRR_1, 151 173 * I-set, 210 301 * ig, 224 286 * Pkinase, 538 810 * |
| ENSG00000140538 | ENSP00000350356 | 831 | TRKC_Mm_TK_Trk | LRRNT, 31 58 * LRR_1, 104 126 * LRR_1, 128 150 * LRR_1, 151 173 * I-set, 210 301 * ig, 224 286 * Pkinase, 530 816 * |
| ENSG00000140538 | ENSP00000354207 | 839 | TRKC_Mm_TK_Trk | LRRNT, 31 58 * LRR_1, 104 126 * LRR_1, 128 150 * LRR_1, 151 173 * I-set, 210 301 * ig, 224 286 * Pkinase, 538 824 * |
| ENSG00000140795 | ENSP00000268446 | 795 | caMLCK_Hs_CAMK_MLCK | Pkinase, 491 746 * |
| ENSG00000140795 | ENSP00000378288 | 819 | caMLCK_Hs_CAMK_MLCK | Pkinase, 515 770 * |
| ENSG00000140992 | ENSP00000344220 | 556 | PDK1_Mm_AGC_PDK1 | Pkinase, 82 342 * |
| ENSG00000140992 | ENSP00000346895 | 532 | PDK1_Mm_AGC_PDK1 | Pkinase, 82 318 * |
| ENSG00000140992 | ENSP00000373876 | 529 | PDK1_Mm_AGC_PDK1 | Pkinase, 55 315 * |
| ENSG00000141068 | ENSP00000268763 | 786 | KSR1_Mm_TKL_RAF | C1_1, 211 258 * Pkinase, 476 740 * |
| ENSG00000141068 | ENSP00000323178 | 923 | KSR1_Mm_TKL_RAF | C1_1, 348 395 * Pkinase, 613 877 * |
| ENSG00000141068 | ENSP00000381952 | 762 | KSR1_Mm_TKL_RAF | C1_1, 211 258 * Pkinase, 476 740 * |
| ENSG00000141068 | ENSP00000381958 | 877 | KSR1_Mm_TKL_RAF | C1_1, 348 395 * Pkinase, 591 855 * |
| ENSG00000141503 | ENSP00000269296 | 1201 | ZC3_Mm_STE_STE20 | Pkinase, 1 187 * CNH, 888 1179 * |
| ENSG00000141503 | ENSP00000347427 | 1230 | ZC3_Mm_STE_STE20 | Pkinase, 1 187 * CNH, 917 1208 * |
| ENSG00000141551 | ENSP00000269361 | 342 | T09B4.7_Ce_CK1_Worm6 | Pkinase, 1 165 * |
| ENSG00000141551 | ENSP00000324464 | 415 | C03C10.1_Ce_CK1_CK1 | Pkinase, 9 273 * |
| ENSG00000141551 | ENSP00000376146 | 409 | C03C10.1_Ce_CK1_CK1 | Pkinase, 9 273 * |
| ENSG00000141639 | ENSP00000383234 | 587 | ERK4_Mm_CMGC_MAPK | Pkinase, 20 312 * |
| ENSG00000141736 | ENSP00000269571 | 1255 | ErbB2_Mm_TK_EGFR | Recep_L_domain, 52 173 * Furin-like, 189 343 * Recep_L_domain, 366 486 * Pkinase, 720 976 * |
| ENSG00000141736 | ENSP00000385185 | 1225 | ErbB2_Mm_TK_EGFR | Recep_L_domain, 22 143 * Furin-like, 159 313 * Recep_L_domain, 336 456 * Pkinase, 690 946 * |
| ENSG00000142149 | ENSP00000270112 | 714 | HUNK_Mm_CAMK_CAMKL | Pkinase, 62 320 * |
| ENSG00000142178 | ENSP00000270162 | 783 | SIK_Mm_CAMK_CAMKL | Pkinase, 27 278 * |
| ENSG00000142208 | ENSP00000385326 | 480 | AKT1_Mm_AGC_Akt | PH, 6 108 * Pkinase, 150 408 * Pkinase_C, 428 478 * |
| ENSG00000142235 | ENSP00000270238 | 1489 | LMR3_Mm_TK_Lmr | Pkinase, 162 437 * |
| ENSG00000142627 | ENSP00000384858 | 976 | EphA2_Hs_TK_Eph | Ephrin_lbd, 28 201 * fn3, 329 424 * fn3, 436 519 * Pkinase, 613 871 * SAM_2, 901 968 * SAM_1, 902 966 * |
| ENSG00000142731 | ENSP00000270861 | 970 | PLK4_Mm_Other_PLK | Pkinase, 12 265 * Sak_Polo, 844 965 * |
| ENSG00000142733 | ENSP00000363152 | 1288 | MAP3K5_Mm_STE_STE11 | Pkinase, 649 906 * |
| ENSG00000142733 | ENSP00000363153 | 797 | MAP3K5_Mm_STE_STE11 | Pkinase, 372 629 * |
| ENSG00000142875 | ENSP00000359713 | 321 | PKACb_Hs_AGC_PKA | Pkinase, 47 268 * |
| ENSG00000142875 | ENSP00000359714 | 264 | PKACb_Hs_AGC_PKA | Pkinase, 50 264 * |
| ENSG00000142875 | ENSP00000359715 | 339 | PKACb_Hs_AGC_PKA | Pkinase, 32 286 * |
| ENSG00000142875 | ENSP00000359716 | 358 | PKACb_Hs_AGC_PKA | Pkinase, 51 305 * |
| ENSG00000142875 | ENSP00000359718 | 245 | PKACb_Hs_AGC_PKA | Pkinase, 32 245 * |
| ENSG00000142875 | ENSP00000359719 | 398 | PKACb_Hs_AGC_PKA | Pkinase, 91 345 * |
| ENSG00000142875 | ENSP00000359722 | 257 | PKACb_Hs_AGC_PKA | Pkinase, 44 257 * |
| ENSG00000142875 | ENSP00000359723 | 351 | PKACb_Hs_AGC_PKA | Pkinase, 44 298 * |
| ENSG00000142875 | ENSP00000378314 | 354 | PKACb_Hs_AGC_PKA | Pkinase, 47 301 * |
| ENSG00000142875 | ENSP00000378315 | 357 | PKACb_Hs_AGC_PKA | Pkinase, 50 304 * |
| ENSG00000143322 | ENSP00000339209 | 1064 | ARG_Mm_TK_Abl | SH3_1, 95 150 * SH3_2, 96 150 * SH2, 158 233 * Pkinase, 273 524 * F_actin_bind, 902 1064 * |
| ENSG00000143322 | ENSP00000356595 | 1182 | ARG_Mm_TK_Abl | SH3_1, 110 165 * SH3_2, 111 165 * SH2, 173 248 * Pkinase, 288 539 * F_actin_bind, 1020 1182 * |
| ENSG00000143322 | ENSP00000375897 | 1008 | ARG_Mm_TK_Abl | SH3_1, 39 94 * SH3_2, 40 94 * SH2, 102 177 * Pkinase, 217 468 * F_actin_bind, 846 1008 * |
| ENSG00000143322 | ENSP00000386152 | 1146 | ARG_Mm_TK_Abl | SH3_1, 74 129 * SH3_2, 75 129 * SH2, 137 212 * Pkinase, 252 503 * F_actin_bind, 984 1146 * |
| ENSG00000143466 | ENSP00000356087 | 716 | IKKe_Mm_Other_IKK | Pkinase, 9 311 * RIO1, 22 170 * |
| ENSG00000143479 | ENSP00000356073 | 568 | DYRK3_Mm_CMGC_DYRK | Pkinase, 189 502 * |
| ENSG00000143479 | ENSP00000356076 | 588 | DYRK3_Mm_CMGC_DYRK | Pkinase, 209 522 * |
| ENSG00000143479 | ENSP00000375797 | 568 | DYRK3_Mm_CMGC_DYRK | Pkinase, 189 502 * |
| ENSG00000143674 | ENSP00000355582 | 570 | MLK4_Hs_TKL_MLK | SH3_1, 41 100 * SH3_2, 42 100 * Pkinase, 124 398 * |
| ENSG00000143674 | ENSP00000355583 | 1036 | MLK4_Hs_TKL_MLK | SH3_1, 41 100 * SH3_2, 42 100 * Pkinase, 124 398 * |
| ENSG00000143776 | ENSP00000355726 | 1691 | MRCKa_Hs_AGC_DMPK | Pkinase, 77 343 * Pkinase_C, 361 408 * M, 453 473 * M, 494 514 * M, 528 548 * M, 713 733 * M, 774 794 * M, 795 815 * DMPK_coil, 881 941 * C1_1, 972 1024 * PH, 1042 1160 * CNH, 1187 1458 * |
| ENSG00000143776 | ENSP00000355727 | 1732 | MRCKa_Hs_AGC_DMPK | Pkinase, 77 343 * Pkinase_C, 361 408 * M, 453 473 * M, 494 514 * M, 528 548 * M, 713 733 * M, 774 794 * M, 795 815 * DMPK_coil, 881 941 * C1_1, 1013 1065 * PH, 1083 1201 * CNH, 1228 1499 * |
| ENSG00000143776 | ENSP00000355728 | 1754 | MRCKa_Hs_AGC_DMPK | Pkinase, 77 343 * Pkinase_C, 361 408 * M, 453 473 * M, 494 514 * M, 528 548 * M, 713 733 * M, 774 794 * M, 795 815 * DMPK_coil, 881 941 * C1_1, 1035 1087 * PH, 1105 1223 * CNH, 1250 1521 * |
| ENSG00000143776 | ENSP00000355729 | 1638 | MRCKa_Hs_AGC_DMPK | Pkinase, 77 343 * Pkinase_C, 361 408 * M, 453 473 * M, 494 514 * M, 528 548 * M, 632 652 * M, 693 713 * M, 714 734 * DMPK_coil, 800 860 * C1_1, 919 971 * PH, 989 1107 * CNH, 1134 1405 * |
| ENSG00000143776 | ENSP00000355731 | 1719 | MRCKa_Hs_AGC_DMPK | Pkinase, 77 343 * Pkinase_C, 361 408 * M, 453 473 * M, 494 514 * M, 528 548 * M, 713 733 * M, 774 794 * M, 795 815 * DMPK_coil, 881 941 * C1_1, 1000 1052 * PH, 1070 1188 * CNH, 1215 1486 * |
| ENSG00000145242 | ENSP00000273854 | 1037 | EphB4_Mm_TK_Eph | Ephrin_lbd, 60 233 * fn3, 358 451 * fn3, 469 552 * Pkinase, 675 932 * SAM_2, 962 1029 * SAM_1, 963 1027 * |
| ENSG00000145242 | ENSP00000346899 | 1015 | EphB4_Mm_TK_Eph | Ephrin_lbd, 60 233 * fn3, 358 451 * fn3, 469 552 * Pkinase, 653 910 * SAM_2, 940 1007 * SAM_1, 941 1005 * |
| ENSG00000145349 | ENSP00000339740 | 499 | CaMK2g_Hs_CAMK_CAMK2 | Pkinase, 14 272 * CaMKII_AD, 346 473 * |
| ENSG00000145349 | ENSP00000369096 | 344 | CaMK2g_Hs_CAMK_CAMK2 | Pkinase, 14 272 * |
| ENSG00000145349 | ENSP00000378032 | 478 | CaMK2g_Hs_CAMK_CAMK2 | Pkinase, 14 272 * CaMKII_AD, 346 473 * |
| ENSG00000145349 | ENSP00000378034 | 489 | CaMK2g_Hs_CAMK_CAMK2 | Pkinase, 14 272 * CaMKII_AD, 357 484 * |
| ENSG00000145632 | ENSP00000274289 | 685 | PLK2_Hs_Other_PLK | Pkinase, 82 334 * POLO_box, 510 573 * POLO_box, 606 677 * |
| ENSG00000145949 | ENSP00000274643 | 388 | caMLCK_Hs_CAMK_MLCK | Pkinase, 106 361 * |
| ENSG00000146216 | ENSP00000259750 | 1321 | TTBK2_Mm_CK1_TTBK | Pkinase, 34 293 * |
| ENSG00000146216 | ENSP00000307357 | 621 | TTBK2_Mm_CK1_TTBK | Pkinase, 1 242 * |
| ENSG00000146216 | ENSP00000377553 | 622 | TTBK2_Mm_CK1_TTBK | Pkinase, 1 242 * |
| ENSG00000146648 | ENSP00000275493 | 1210 | EGFR_Mm_TK_EGFR | Recep_L_domain, 57 168 * Furin-like, 184 338 * Recep_L_domain, 361 481 * Pkinase, 712 968 * |
| ENSG00000146872 | ENSP00000316512 | 772 | TLK2_Hs_Other_TLK | Pkinase, 462 741 * |
| ENSG00000146872 | ENSP00000340800 | 718 | TLK2_Hs_Other_TLK | Pkinase, 408 687 * |
| ENSG00000146872 | ENSP00000275780 | 750 | TLK2_Hs_Other_TLK | Pkinase, 440 719 * |
| ENSG00000146904 | ENSP00000275815 | 976 | EphA1_Hs_TK_Eph | Ephrin_lbd, 27 204 * fn3, 333 431 * fn3, 448 528 * Pkinase, 624 880 * SAM_2, 910 976 * SAM_1, 911 975 * |
| ENSG00000147044 | ENSP00000322727 | 926 | CASK_Hs_CAMK_CASK | Pkinase, 12 276 * L27, 346 401 * L27, 405 458 * PDZ, 490 568 * SH3_1, 615 680 * SH3_2, 616 680 * Guanylate_kin, 775 879 * |
| ENSG00000147044 | ENSP00000354641 | 909 | CASK_Hs_CAMK_CASK | Pkinase, 12 276 * L27, 346 401 * L27, 405 458 * PDZ, 490 568 * SH3_1, 603 668 * SH3_2, 604 668 * Guanylate_kin, 758 862 * |
| ENSG00000147044 | ENSP00000367396 | 590 | CASK_Hs_CAMK_CASK | Pkinase, 12 276 * L27, 346 401 * L27, 405 458 * PDZ, 490 568 * |
| ENSG00000147044 | ENSP00000367400 | 909 | CASK_Hs_CAMK_CASK | Pkinase, 12 276 * L27, 346 401 * L27, 405 458 * PDZ, 490 568 * SH3_1, 603 668 * SH3_2, 604 668 * Guanylate_kin, 758 862 * |
| ENSG00000147044 | ENSP00000367405 | 926 | CASK_Hs_CAMK_CASK | Pkinase, 12 276 * L27, 346 401 * L27, 405 458 * PDZ, 490 568 * SH3_1, 615 680 * SH3_2, 616 680 * Guanylate_kin, 775 879 * |
| ENSG00000147044 | ENSP00000367408 | 921 | CASK_Hs_CAMK_CASK | Pkinase, 12 276 * L27, 346 401 * L27, 405 458 * PDZ, 490 568 * SH3_1, 615 680 * SH3_2, 616 680 * Guanylate_kin, 770 874 * |
| ENSG00000147044 | ENSP00000367410 | 859 | CASK_Hs_CAMK_CASK | Pkinase, 1 209 * L27, 279 334 * L27, 338 391 * PDZ, 423 501 * SH3_1, 548 613 * SH3_2, 549 613 * Guanylate_kin, 708 812 * |
| ENSG00000147044 | ENSP00000367421 | 897 | CASK_Hs_CAMK_CASK | Pkinase, 12 276 * L27, 340 395 * L27, 399 452 * PDZ, 484 562 * SH3_1, 586 651 * SH3_2, 587 651 * Guanylate_kin, 746 850 * |
| ENSG00000147507 | ENSP00000276497 | 582 | LYN_Mm_TK_Src | SH3_1, 136 191 * SH3_2, 137 191 * SH2, 199 281 * Pkinase, 317 570 * |
| ENSG00000147507 | ENSP00000385606 | 512 | LYN_Mm_TK_Src | SH3_1, 66 121 * SH3_2, 67 121 * SH2, 129 211 * Pkinase, 247 500 * |
| ENSG00000148053 | ENSP00000314586 | 822 | TRKB_Mm_TK_Trk | LRRNT, 31 60 * I-set, 197 283 * V-set, 198 284 * Pkinase, 538 810 * |
| ENSG00000148053 | ENSP00000365387 | 838 | TRKB_Mm_TK_Trk | LRRNT, 31 60 * I-set, 197 283 * V-set, 198 284 * Pkinase, 554 826 * |
| ENSG00000148660 | ENSP00000307082 | 529 | CaMK2g_Hs_CAMK_CAMK2 | Pkinase, 14 272 * CaMKII_AD, 397 524 * |
| ENSG00000148660 | ENSP00000315599 | 527 | CaMK2g_Hs_CAMK_CAMK2 | Pkinase, 14 272 * CaMKII_AD, 395 522 * |
| ENSG00000148660 | ENSP00000319060 | 556 | CaMK2g_Hs_CAMK_CAMK2 | Pkinase, 14 272 * CaMKII_AD, 424 551 * |
| ENSG00000148660 | ENSP00000277853 | 495 | CaMK2g_Hs_CAMK_CAMK2 | Pkinase, 14 272 * CaMKII_AD, 363 490 * |
| ENSG00000148660 | ENSP00000361851 | 516 | CaMK2g_Hs_CAMK_CAMK2 | Pkinase, 14 272 * CaMKII_AD, 384 511 * |
| ENSG00000148660 | ENSP00000378243 | 524 | CaMK2g_Hs_CAMK_CAMK2 | Pkinase, 14 272 * CaMKII_AD, 392 519 * |
| ENSG00000148660 | ENSP00000378244 | 558 | CaMK2g_Hs_CAMK_CAMK2 | Pkinase, 14 272 * CaMKII_AD, 426 553 * |
| ENSG00000149269 | ENSP00000278568 | 553 | PAK3_Mm_STE_STE20 | PBD, 74 132 * Pkinase, 270 521 * APH, 272 448 * |
| ENSG00000149269 | ENSP00000348696 | 545 | PAK3_Mm_STE_STE20 | PBD, 74 132 * Pkinase, 270 521 * APH, 272 448 * |
| ENSG00000149554 | ENSP00000278916 | 476 | CHK1_Mm_CAMK_CAMKL | Pkinase, 9 265 * |
| ENSG00000149930 | ENSP00000279394 | 1049 | TAO2_Mm_STE_STE20 | Pkinase, 28 281 * |
| ENSG00000149930 | ENSP00000310094 | 1235 | TAO2_Mm_STE_STE20 | Pkinase, 28 281 * |
| ENSG00000150457 | ENSP00000372035 | 1088 | LATS2_Hs_AGC_NDR | UBA, 99 136 * Pkinase, 668 973 * Pkinase_C, 992 1046 * |
| ENSG00000151292 | ENSP00000334735 | 423 | gish_Dm_CK1_CK1 | Pkinase, 43 308 * |
| ENSG00000151292 | ENSP00000353904 | 455 | gish_Dm_CK1_CK1 | Pkinase, 43 308 * |
| ENSG00000151292 | ENSP00000354942 | 447 | gish_Dm_CK1_CK1 | Pkinase, 43 308 * |
| ENSG00000151414 | ENSP00000356355 | 302 | NEK7_Mm_Other_NEK | Pkinase, 34 291 * |
| ENSG00000151422 | ENSP00000281092 | 822 | FER_Hs_TK_Fer | FCH, 1 92 * SH2, 460 531 * Pkinase, 563 814 * |
| ENSG00000152332 | ENSP00000282169 | 419 | KIS_Mm_Other_KIS | Pkinase, 23 304 * RRM_1, 345 400 * |
| ENSG00000152332 | ENSP00000375995 | 344 | KIS_Mm_Other_KIS | Pkinase, 23 304 * |
| ENSG00000152495 | ENSP00000282356 | 473 | CaMK4_Mm_CAMK_CAMK1 | Pkinase, 46 300 * |
| ENSG00000152953 | ENSP00000282908 | 414 | YANK2_Mm_AGC_YANK | Pkinase, 23 283 * |
| ENSG00000152953 | ENSP00000381030 | 402 | YANK2_Mm_AGC_YANK | Pkinase, 11 271 * |
| ENSG00000153208 | ENSP00000295408 | 999 | MER_Mm_TK_Axl | V-set, 64 194 * ig, 108 177 * ig, 211 264 * fn3, 284 368 * fn3, 383 473 * Pkinase, 587 854 * |
| ENSG00000153208 | ENSP00000376929 | 556 | MER_Mm_TK_Axl | Pkinase, 229 485 * |
| ENSG00000153208 | ENSP00000387277 | 823 | MER_Mm_TK_Axl | fn3, 108 192 * fn3, 207 297 * Pkinase, 411 678 * |
| ENSG00000154229 | ENSP00000284384 | 672 | PKCa_Hs_AGC_PKC | C1_1, 37 89 * C1_1, 102 154 * C1_3, 113 144 * C2, 173 260 * Pkinase, 339 597 * Pkinase_C, 617 662 * |
| ENSG00000154237 | ENSP00000284395 | 2039 | LRRK1_Hs_TKL_LRRK | LRR_1, 303 325 * LRR_1, 327 347 * LRR_1, 354 375 * LRR_1, 377 400 * LRR_1, 405 427 * LRR_1, 429 451 * LRR_1, 475 496 * LRR_1, 498 520 * LRR_1, 522 540 * LRR_1, 573 594 * LRR_1, 596 618 * Arf, 646 846 * Miro, 664 784 * LRR_1, 1208 1229 * Pkinase, 1266 1544 * |
| ENSG00000154237 | ENSP00000373600 | 2015 | LRRK1_Hs_TKL_LRRK | LRR_1, 279 301 * LRR_1, 303 323 * LRR_1, 330 351 * LRR_1, 353 376 * LRR_1, 381 403 * LRR_1, 405 427 * LRR_1, 451 472 * LRR_1, 474 496 * LRR_1, 498 516 * LRR_1, 549 570 * LRR_1, 572 594 * Arf, 622 822 * Miro, 640 760 * LRR_1, 1184 1205 * Pkinase, 1242 1520 * |
| ENSG00000154310 | ENSP00000284483 | 1367 | TNIK_Hs_STE_STE20 | Pkinase, 25 289 * CNH, 1054 1345 * |
| ENSG00000154310 | ENSP00000345352 | 1312 | TNIK_Hs_STE_STE20 | Pkinase, 25 289 * CNH, 999 1290 * |
| ENSG00000154310 | ENSP00000349880 | 1338 | TNIK_Hs_STE_STE20 | Pkinase, 25 289 * CNH, 1025 1316 * |
| ENSG00000154358 | ENSP00000355670 | 7969 | Obscn_Mm_CAMK_Trio | V-set, 9 100 * I-set, 10 99 * ig, 24 84 * V-set, 108 202 * I-set, 110 201 * ig, 124 186 * V-set, 237 329 * I-set, 241 328 * ig, 252 313 * V-set, 332 419 * I-set, 333 418 * ig, 347 406 * I-set, 420 505 * fn3, 513 601 * ig, 721 780 * V-set, 788 884 * I-set, 807 883 * ig, 812 872 * I-set, 889 975 * ig, 905 964 * Mastoparan_2, 921 934 * I-set, 982 1067 * V-set, 985 1066 * ig, 997 1056 * Mastoparan_2, 1013 1026 * I-set, 1074 1159 * ig, 1089 1148 * Mastoparan_2, 1105 1118 * I-set, 1166 1251 * V-set, 1169 1250 * ig, 1181 1240 * Mastoparan_2, 1197 1210 * I-set, 1258 1343 * V-set, 1261 1344 * ig, 1273 1332 * Mastoparan_2, 1289 1302 * I-set, 1349 1435 * ig, 1365 1424 * Mastoparan_2, 1381 1394 * I-set, 1442 1527 * ig, 1457 1516 * V-set, 1532 1620 * I-set, 1534 1619 * ig, 1549 1608 * Mastoparan_2, 1565 1578 * V-set, 1625 1712 * I-set, 1626 1711 * ig, 1641 1700 * Mastoparan_2, 1 |
| ENSG00000154928 | ENSP00000354191 | 943 | EphB4_Mm_TK_Eph | Ephrin_lbd, 19 196 * fn3, 323 414 * fn3, 434 518 * Pkinase, 619 837 * SAM_2, 867 934 * SAM_1, 868 932 * |
| ENSG00000154928 | ENSP00000381097 | 1057 | EphB4_Mm_TK_Eph | Ephrin_lbd, 92 269 * fn3, 396 487 * fn3, 507 591 * Pkinase, 692 951 * SAM_2, 981 1048 * SAM_1, 982 1046 * |
| ENSG00000155111 | ENSP00000317665 | 442 | CDK8_Mm_CMGC_CDK | Pkinase, 2 275 * |
| ENSG00000155111 | ENSP00000357907 | 502 | CDK8_Mm_CMGC_CDK | Pkinase, 20 335 * |
| ENSG00000155111 | ENSP00000376355 | 441 | CDK8_Mm_CMGC_CDK | Pkinase, 1 274 * |
| ENSG00000155657 | ENSP00000364178 | 31,972 | TTN_Mm_CAMK_MLCK | V-set, 5 98 * I-set, 6 97 * ig, 20 82 * I-set, 104 193 * V-set, 104 194 * ig, 118 178 * Titin_Z, 413 455 * Titin_Z, 464 505 * Titin_Z, 510 549 * Titin_Z, 552 593 * Titin_Z, 598 639 * Titin_Z, 644 685 * Titin_Z, 690 732 * C2-set_2, 943 1026 * I-set, 943 1032 * V-set, 943 1035 * ig, 957 1017 * I-set, 1082 1173 * V-set, 1082 1174 * ig, 1096 1158 * I-set, 1291 1381 * ig, 1305 1366 * I-set, 1457 1547 * ig, 1471 1532 * I-set, 1556 1647 * ig, 1570 1632 * I-set, 1703 1794 * I-set, 1841 1929 * V-set, 1842 1930 * ig, 1855 1914 * PPAK, 1908 1935 * I-set, 2078 2168 * ig, 2092 2153 * V-set, 2173 2263 * I-set, 2174 2262 * ig, 2189 2248 * I-set, 2267 2353 * ig, 2281 2340 * I-set, 2356 2442 * ig, 2370 2429 * V-set, 2444 2531 * I-set, 2445 2530 * ig, 2459 2519 * I-set, 2533 2617 * ig, 2547 2606 * I-set, 2620 2704 * ig, 2634 2693 * I-set, 2707 2792 * V-set, 2794 2880 * I-set, 2795 |
| ENSG00000156345 | ENSP00000286878 | 450 | CCRK_Mm_CMGC_CDK | Pkinase, 4 267 * |
| ENSG00000156345 | ENSP00000322343 | 346 | CCRK_Mm_CMGC_CDK | Pkinase, 4 288 * |
| ENSG00000156345 | ENSP00000338975 | 338 | CCRK_Mm_CMGC_CDK | Pkinase, 4 280 * |
| ENSG00000156345 | ENSP00000365043 | 325 | CCRK_Mm_CMGC_CDK | Pkinase, 4 267 * |
| ENSG00000156711 | ENSP00000211287 | 365 | p38d_Mm_CMGC_MAPK | Pkinase, 25 308 * |
| ENSG00000156711 | ENSP00000362866 | 355 | p38d_Mm_CMGC_MAPK | Pkinase, 25 298 * |
| ENSG00000156711 | ENSP00000362875 | 310 | p38d_Mm_CMGC_MAPK | Pkinase, 25 253 * |
| ENSG00000156873 | ENSP00000329968 | 406 | PHKg2_Mm_CAMK_PHK | Pkinase, 24 291 * |
| ENSG00000156970 | ENSP00000287598 | 1050 | BUBR1_Hs_Other_BUB | Mad3_BUB1_I, 55 179 * |
| ENSG00000157404 | ENSP00000288135 | 976 | KIT_Hs_TK_PDGFR | V-set, 210 308 * I-set, 212 307 * ig, 226 292 * Pkinase, 589 924 * |
| ENSG00000157540 | ENSP00000319032 | 541 | DYRK1A_Hs_CMGC_DYRK | Pkinase, 159 479 * |
| ENSG00000157540 | ENSP00000342690 | 584 | DYRK1A_Hs_CMGC_DYRK | Pkinase, 159 479 * |
| ENSG00000157540 | ENSP00000340373 | 754 | DYRK1A_Hs_CMGC_DYRK | Pkinase, 150 470 * |
| ENSG00000157540 | ENSP00000381929 | 529 | DYRK1A_Hs_CMGC_DYRK | Pkinase, 159 479 * |
| ENSG00000157540 | ENSP00000381932 | 763 | DYRK1A_Hs_CMGC_DYRK | Pkinase, 159 479 * |
| ENSG00000157764 | ENSP00000288602 | 766 | RAF1_Hs_TKL_RAF | RBD, 155 227 * C1_1, 235 283 * C1_3, 246 273 * Pkinase, 457 714 * |
| ENSG00000158828 | ENSP00000364204 | 581 | PINK1_Hs_Other_NKF2 | Pkinase, 156 512 * |
| ENSG00000159792 | ENSP00000291041 | 424 | PSKH1_Mm_CAMK_PSK | Pkinase, 98 355 * |
| ENSG00000160145 | ENSP00000291478 | 1289 | Trad_Hs_CAMK_Trio | RhoGEF, 236 406 * PH, 420 529 * V-set, 773 869 * I-set, 774 868 * ig, 788 853 * fn3, 872 957 * Pkinase, 987 1241 * |
| ENSG00000160145 | ENSP00000346122 | 1257 | Trad_Hs_CAMK_Trio | RhoGEF, 204 374 * PH, 388 497 * V-set, 741 837 * I-set, 742 836 * ig, 756 821 * fn3, 840 925 * Pkinase, 955 1209 * |
| ENSG00000160145 | ENSP00000353109 | 2986 | Trad_Hs_CAMK_Trio | Spectrin, 188 308 * Spectrin, 310 416 * Spectrin, 536 642 * Spectrin, 890 1004 * Spectrin, 1130 1236 * RhoGEF, 1285 1455 * PH, 1475 1580 * SH3_1, 1649 1709 * SH3_2, 1650 1709 * RhoGEF, 1933 2103 * PH, 2117 2226 * SH3_2, 2325 2384 * V-set, 2470 2566 * I-set, 2471 2565 * ig, 2485 2550 * fn3, 2569 2654 * Pkinase, 2684 2938 * |
| ENSG00000160396 | ENSP00000291823 | 616 | HIPK3_Mm_CMGC_DYRK | Pkinase, 11 347 * |
| ENSG00000160447 | ENSP00000291906 | 889 | PKN_Sp_AGC_PKN | HR1, 18 90 * HR1, 105 177 * HR1, 178 255 * Pkinase, 559 818 * Pkinase_C, 838 885 * |
| ENSG00000160469 | ENSP00000310649 | 778 | BRSK1_Hs_CAMK_CAMKL | Pkinase, 34 285 * |
| ENSG00000160469 | ENSP00000320853 | 793 | BRSK1_Hs_CAMK_CAMKL | Pkinase, 49 300 * |
| ENSG00000160551 | ENSP00000261716 | 1001 | TAO1_Mm_STE_STE20 | Pkinase, 28 281 * |
| ENSG00000160584 | ENSP00000292055 | 1263 | QSK_Mm_CAMK_CAMKL | Pkinase, 8 259 * |
| ENSG00000160584 | ENSP00000364449 | 1321 | QSK_Mm_CAMK_CAMKL | Pkinase, 66 317 * |
| ENSG00000160602 | ENSP00000268766 | 692 | NEK8_Hs_Other_NEK | Pkinase, 4 258 * RCC1, 409 458 * RCC1, 461 510 * RCC1, 579 628 * RCC1, 631 679 * |
| ENSG00000160867 | ENSP00000292408 | 802 | FGFR4_Mm_TK_FGFR | V-set, 35 119 * ig, 50 103 * V-set, 152 242 * I-set, 153 241 * ig, 165 226 * I-set, 249 350 * V-set, 249 351 * ig, 264 335 * Pkinase, 467 743 * |
| ENSG00000160867 | ENSP00000366412 | 1030 | FGFR4_Mm_TK_FGFR | V-set, 111 195 * ig, 126 179 * V-set, 264 354 * I-set, 265 353 * ig, 277 338 * I-set, 361 462 * V-set, 361 463 * ig, 376 447 * Pkinase, 695 971 * |
| ENSG00000160867 | ENSP00000377254 | 762 | FGFR4_Mm_TK_FGFR | V-set, 35 119 * ig, 50 103 * V-set, 152 242 * I-set, 153 241 * ig, 165 226 * I-set, 249 350 * V-set, 249 351 * ig, 264 335 * Pkinase, 427 703 * |
| ENSG00000162302 | ENSP00000294261 | 524 | MSK2_Mm_AGC_RSK | Pkinase, 33 301 * Pkinase_C, 321 365 * |
| ENSG00000162302 | ENSP00000333896 | 772 | MSK2_Mm_AGC_RSK | Pkinase, 33 301 * Pkinase_C, 321 365 * Pkinase, 411 674 * |
| ENSG00000162409 | ENSP00000360290 | 552 | AMPKa2_Mm_CAMK_CAMKL | Pkinase, 16 268 * |
| ENSG00000162526 | ENSP00000362634 | 268 | TSSK3_Hs_CAMK_TSSK | Pkinase, 10 265 * |
| ENSG00000162733 | ENSP00000356898 | 855 | DDR2_Hs_TK_DDR | F5_F8_type_C, 45 182 * Pkinase, 563 849 * |
| ENSG00000162889 | ENSP00000294981 | 370 | MAPKAPK2_Mm_CAMK_MAPKAPK | Pkinase, 64 325 * |
| ENSG00000162889 | ENSP00000356070 | 400 | MAPKAPK2_Mm_CAMK_MAPKAPK | Pkinase, 64 325 * |
| ENSG00000163349 | ENSP00000358568 | 1165 | HIPK2_Mm_CMGC_DYRK | Pkinase, 190 518 * |
| ENSG00000163349 | ENSP00000358571 | 1210 | HIPK2_Mm_CMGC_DYRK | Pkinase, 190 518 * |
| ENSG00000163349 | ENSP00000358572 | 1075 | HIPK2_Mm_CMGC_DYRK | Pkinase, 190 518 * |
| ENSG00000163482 | ENSP00000295709 | 1315 | Fused_Hs_Other_ULK | Pkinase, 4 254 * HEAT, 558 597 * HEAT, 1109 1140 * HEAT, 1147 1183 * HEAT, 1187 1223 * HEAT, 1232 1264 * |
| ENSG00000163482 | ENSP00000375954 | 1294 | Fused_Hs_Other_ULK | Pkinase, 4 254 * HEAT, 558 597 * HEAT, 1088 1119 * HEAT, 1126 1162 * HEAT, 1166 1202 * HEAT, 1211 1243 * |
| ENSG00000163482 | ENSP00000375955 | 1294 | Fused_Hs_Other_ULK | Pkinase, 4 254 * HEAT, 558 597 * HEAT, 1088 1119 * HEAT, 1126 1162 * HEAT, 1166 1202 * HEAT, 1211 1243 * |
| ENSG00000163491 | ENSP00000350059 | 404 | 6348_Tt_Other_NEK | Pkinase, 3 180 * |
| ENSG00000163491 | ENSP00000379867 | 717 | NEK10_Sp_Other_NEK | Arm, 198 238 * Arm, 239 279 * Arm, 280 320 * Pkinase, 519 715 * |
| ENSG00000163491 | ENSP00000379876 | 559 | 6348_Tt_Other_NEK | Pkinase, 3 180 * |
| ENSG00000163513 | ENSP00000295754 | 567 | TGFBR2_Mm_TKL_STKR | ecTbetaR2, 47 165 * Pkinase, 244 538 * |
| ENSG00000163513 | ENSP00000351905 | 592 | TGFBR2_Mm_TKL_STKR | ecTbetaR2, 72 190 * Pkinase, 269 563 * |
| ENSG00000163545 | ENSP00000356125 | 628 | NuaK2_Mm_CAMK_CAMKL | Pkinase, 53 303 * |
| ENSG00000163558 | ENSP00000376483 | 596 | aPKC_Dm_AGC_PKC | PB1, 25 108 * C1_1, 141 193 * Pkinase, 254 522 * Pkinase_C, 542 588 * |
| ENSG00000163673 | ENSP00000295943 | 648 | DCAMKL3_Mm_CAMK_DCAMKL | Pkinase, 356 613 * |
| ENSG00000163785 | ENSP00000296084 | 608 | RYK_Mm_TK_Ryk | WIF, 64 192 * Pkinase, 331 597 * |
| ENSG00000163788 | ENSP00000296088 | 765 | SNRK_Mm_CAMK_CAMKL | Pkinase, 16 269 * |
| ENSG00000163932 | ENSP00000331602 | 676 | PKCt_Mm_AGC_PKC | C1_1, 159 211 * C1_3, 170 201 * C1_1, 231 283 * C1_3, 242 273 * Pkinase, 349 603 * Pkinase_C, 623 669 * |
| ENSG00000164078 | ENSP00000296474 | 1400 | RON_Hs_TK_Met | Sema, 58 507 * PSI, 526 568 * TIG, 569 670 * TIG, 684 766 * TIG, 770 859 * Pkinase, 1082 1341 * |
| ENSG00000164078 | ENSP00000341325 | 1351 | RON_Hs_TK_Met | Sema, 58 507 * PSI, 526 568 * TIG, 569 670 * TIG, 684 766 * TIG, 770 859 * Pkinase, 1033 1292 * |
| ENSG00000164543 | ENSP00000319192 | 414 | DRAK2_Hs_CAMK_DAPK | Pkinase, 61 321 * |
| ENSG00000164715 | ENSP00000297293 | 1503 | LMR2_Hs_TK_Lmr | Pkinase, 137 407 * |
| ENSG00000164776 | ENSP00000297373 | 387 | PHKg1_Mm_CAMK_PHK | Pkinase, 20 288 * |
| ENSG00000164776 | ENSP00000378817 | 390 | PHKg1_Mm_CAMK_PHK | Pkinase, 20 291 * |
| ENSG00000164885 | ENSP00000297518 | 292 | CDK5_Mm_CMGC_CDK | Pkinase, 4 286 * |
| ENSG00000165025 | ENSP00000364898 | 635 | SYK_Mm_TK_Syk | SH2, 15 92 * SH2, 168 244 * Pkinase, 371 626 * |
| ENSG00000165025 | ENSP00000364904 | 612 | SYK_Mm_TK_Syk | SH2, 15 92 * SH2, 168 244 * Pkinase, 348 603 * |
| ENSG00000165059 | ENSP00000366488 | 351 | PKACa_Hs_AGC_PKA | Pkinase, 44 298 * |
| ENSG00000165238 | ENSP00000297954 | 2297 | Wnk3_Mm_Other_WNK | Pkinase, 195 453 * |
| ENSG00000165238 | ENSP00000297876 | 2224 | Wnk3_Mm_Other_WNK | Pkinase, 195 453 * |
| ENSG00000165238 | ENSP00000348347 | 2217 | Wnk3_Mm_Other_WNK | Pkinase, 195 453 * |
| ENSG00000165238 | ENSP00000378858 | 2193 | Wnk3_Mm_Other_WNK | Pkinase, 181 439 * |
| ENSG00000165238 | ENSP00000378860 | 2254 | Wnk3_Mm_Other_WNK | Pkinase, 195 453 * |
| ENSG00000165304 | ENSP00000298048 | 651 | MELK_Mm_CAMK_CAMKL | Pkinase, 11 263 * KA1, 605 651 * |
| ENSG00000165731 | ENSP00000344798 | 1114 | RET_Mm_TK_Ret | Cadherin, 172 261 * Pkinase, 724 1005 * |
| ENSG00000165731 | ENSP00000347942 | 1107 | RET_Mm_TK_Ret | Cadherin, 172 261 * Pkinase, 724 1005 * |
| ENSG00000165752 | ENSP00000298630 | 486 | YANK3_Mm_AGC_YANK | Pkinase, 93 353 * |
| ENSG00000165752 | ENSP00000357609 | 381 | YANK3_Mm_AGC_YANK | Pkinase, 32 292 * |
| ENSG00000165752 | ENSP00000357611 | 369 | YANK3_Mm_AGC_YANK | Pkinase, 2 236 * |
| ENSG00000166483 | ENSP00000299613 | 646 | Wee1B_Mm_Other_WEE | Pkinase, 299 569 * |
| ENSG00000166484 | ENSP00000311005 | 677 | ERK5_Mm_CMGC_MAPK | Pkinase, 1 208 * |
| ENSG00000166484 | ENSP00000378968 | 816 | ERK5_Mm_CMGC_MAPK | Pkinase, 55 347 * |
| ENSG00000166501 | ENSP00000305355 | 673 | PKCa_Hs_AGC_PKC | C1_1, 37 89 * C1_1, 102 154 * C2, 173 260 * Pkinase, 342 600 * Pkinase_C, 620 665 * |
| ENSG00000166501 | ENSP00000318315 | 671 | PKCa_Hs_AGC_PKC | C1_1, 37 89 * C1_1, 102 154 * C2, 173 260 * Pkinase, 342 600 * Pkinase_C, 620 666 * |
| ENSG00000166851 | ENSP00000300093 | 603 | PLK1_Hs_Other_PLK | Pkinase, 53 305 * POLO_box, 417 480 * POLO_box, 515 584 * |
| ENSG00000166851 | ENSP00000331713 | 247 | PLK1_Hs_Other_PLK | Pkinase, 53 247 * |
| ENSG00000167258 | ENSP00000300647 | 1490 | CRK7_Mm_CMGC_CDK | Pkinase, 727 1020 * |
| ENSG00000167524 | ENSP00000301037 | 274 | PRKY_Hs_AGC_PKA | Pkinase, 107 274 * |
| ENSG00000167601 | ENSP00000301178 | 894 | AXL_Mm_TK_Axl | V-set, 34 138 * ig, 49 119 * ig, 153 207 * fn3, 225 321 * fn3, 334 418 * Pkinase, 536 803 * |
| ENSG00000167601 | ENSP00000351995 | 885 | AXL_Mm_TK_Axl | V-set, 34 138 * ig, 49 119 * ig, 153 207 * fn3, 225 321 * fn3, 334 418 * Pkinase, 527 794 * |
| ENSG00000167657 | ENSP00000301264 | 454 | DAPK3_Mm_CAMK_DAPK | Pkinase, 13 275 * |
| ENSG00000168038 | ENSP00000301831 | 1275 | ULK4_Mm_Other_ULK | Pkinase, 4 280 * HEAT, 504 541 * HEAT, 641 679 * HEAT, 731 763 * HEAT, 926 962 * HEAT, 1151 1187 * |
| ENSG00000168067 | ENSP00000294066 | 820 | GCK_Mm_STE_STE20 | Pkinase, 16 273 * CNH, 488 800 * |
| ENSG00000168067 | ENSP00000366567 | 812 | GCK_Mm_STE_STE20 | Pkinase, 16 273 * CNH, 480 792 * |
| ENSG00000168078 | ENSP00000301905 | 322 | PBK_Mm_Other_TOPK | Pkinase, 32 318 * |
| ENSG00000169032 | ENSP00000302486 | 393 | MAP2K1_Hs_STE_STE7 | Pkinase, 68 361 * |
| ENSG00000169071 | ENSP00000364860 | 943 | ROR2_Mm_TK_Ror | V-set, 34 153 * I-set, 62 152 * ig, 76 137 * Fz, 168 301 * Kringle, 316 394 * Pkinase, 473 746 * |
| ENSG00000169071 | ENSP00000364862 | 704 | ROR2_Mm_TK_Ror | Fz, 28 161 * Kringle, 176 254 * Pkinase, 333 606 * |
| ENSG00000169071 | ENSP00000364867 | 704 | ROR2_Mm_TK_Ror | Fz, 28 161 * Kringle, 176 254 * Pkinase, 333 606 * |
| ENSG00000169118 | ENSP00000307753 | 393 | gish_Dm_CK1_CK1 | Pkinase, 44 311 * |
| ENSG00000169118 | ENSP00000305777 | 422 | gish_Dm_CK1_CK1 | Pkinase, 44 311 * |
| ENSG00000169302 | ENSP00000303165 | 358 | YANK1_Mm_AGC_YANK | Pkinase, 23 281 * |
| ENSG00000169302 | ENSP00000381535 | 396 | YANK1_Mm_AGC_YANK | Pkinase, 23 281 * |
| ENSG00000169398 | ENSP00000341189 | 1052 | FAK_Mm_TK_FAK | FERM_M, 139 258 * Pkinase, 422 676 * Focal_AT, 914 1052 * |
| ENSG00000169398 | ENSP00000342839 | 553 | FAK_Mm_TK_FAK | Pkinase, 240 522 * |
| ENSG00000169398 | ENSP00000346424 | 877 | FAK_Mm_TK_FAK | Pkinase, 240 522 * Focal_AT, 739 877 * |
| ENSG00000169398 | ENSP00000378640 | 894 | FAK_Mm_TK_FAK | Pkinase, 236 518 * Focal_AT, 756 894 * |
| ENSG00000169398 | ENSP00000378644 | 1006 | FAK_Mm_TK_FAK | FERM_M, 139 258 * Pkinase, 422 676 * Focal_AT, 868 1006 * |
| ENSG00000169398 | ENSP00000378647 | 1007 | FAK_Mm_TK_FAK | FERM_M, 49 168 * Pkinase, 374 628 * Focal_AT, 869 1007 * |
| ENSG00000169398 | ENSP00000378649 | 1073 | FAK_Mm_TK_FAK | FERM_M, 160 279 * Pkinase, 443 697 * Focal_AT, 935 1073 * |
| ENSG00000169679 | ENSP00000302530 | 1085 | BUB1_Hs_Other_BUB | Mad3_BUB1_I, 4 126 * Pkinase, 787 1073 * |
| ENSG00000169967 | ENSP00000343463 | 635 | MAP3K2_Hs_STE_STE11 | PB1, 59 138 * Pkinase, 372 632 * |
| ENSG00000170145 | ENSP00000305976 | 926 | QIK_Mm_CAMK_CAMKL | Pkinase, 20 271 * |
| ENSG00000170209 | ENSP00000306678 | 765 | SgK288_Hs_TKL_RIPK | Pkinase, 26 281 * Ank, 361 393 * Ank, 394 426 * Ank, 427 459 * Ank, 460 492 * Ank, 493 525 * Ank, 526 558 * Ank, 559 591 * Ank, 592 624 * Ank, 625 657 * Ank, 658 690 * Ank, 691 723 * |
| ENSG00000170312 | ENSP00000306043 | 297 | CDC2_Mm_CMGC_CDK | Pkinase, 4 287 * |
| ENSG00000170312 | ENSP00000378699 | 301 | CDC2_Mm_CMGC_CDK | Pkinase, 4 291 * |
| ENSG00000170390 | ENSP00000296550 | 766 | DCAMKL2_Mm_CAMK_DCAMKL | DCX, 89 153 * DCX, 214 275 * Pkinase, 394 651 * |
| ENSG00000170390 | ENSP00000303887 | 783 | DCAMKL2_Mm_CAMK_DCAMKL | DCX, 89 153 * DCX, 214 275 * Pkinase, 411 668 * |
| ENSG00000170390 | ENSP00000377578 | 695 | DCAMKL2_Mm_CAMK_DCAMKL | DCX, 89 153 * DCX, 214 275 * Pkinase, 393 650 * |
| ENSG00000171094 | ENSP00000373700 | 1620 | ALK_Mm_TK_ALK | Ldl_recept_a, 423 471 * MAM, 480 636 * Pkinase, 1116 1383 * |
| ENSG00000171105 | ENSP00000303830 | 1382 | INSR_Mm_TK_InsR | Recep_L_domain, 52 164 * Furin-like, 179 340 * Recep_L_domain, 359 474 * fn3, 854 937 * Pkinase, 1023 1290 * |
| ENSG00000171105 | ENSP00000342838 | 1370 | INSR_Mm_TK_InsR | Recep_L_domain, 52 164 * Furin-like, 179 340 * Recep_L_domain, 359 474 * fn3, 842 925 * Pkinase, 1011 1278 * |
| ENSG00000171132 | ENSP00000306124 | 737 | PKCeps_Mm_AGC_PKC | C2, 8 99 * C1_1, 170 223 * C1_1, 243 295 * Pkinase, 408 668 * Pkinase_C, 688 734 * |
| ENSG00000171219 | ENSP00000345133 | 1551 | DMPK2_Hs_AGC_DMPK | Pkinase, 71 337 * Pkinase_C, 355 402 * DMPK_coil, 744 801 * C1_1, 879 931 * PH, 948 1066 * CNH, 1093 1366 * |
| ENSG00000171435 | ENSP00000305466 | 646 | KSR2_Hs_TKL_RAF | C1_1, 110 157 * Pkinase, 363 625 * |
| ENSG00000171435 | ENSP00000339952 | 829 | KSR2_Hs_TKL_RAF | C1_1, 292 339 * Pkinase, 545 807 * |
| ENSG00000172071 | ENSP00000307235 | 1116 | PEK_Hs_Other_PEK | PQQ, 104 142 * PQQ, 234 271 * Pkinase, 593 1072 * |
| ENSG00000172680 | ENSP00000310722 | 346 | MOS_Hs_Other_MOS | Pkinase, 60 338 * |
| ENSG00000172939 | ENSP00000311713 | 527 | OSR1_Mm_STE_STE20 | Pkinase, 17 291 * |
| ENSG00000173020 | ENSP00000312262 | 689 | BARK1_Hs_AGC_GRK | RGS, 54 174 * Pkinase, 191 453 * PH, 559 652 * |
| ENSG00000173327 | ENSP00000309597 | 847 | MLK3_Mm_TKL_MLK | SH3_1, 44 103 * SH3_2, 45 103 * Pkinase, 117 376 * |
| ENSG00000173334 | ENSP00000312150 | 372 | Trb1_Hs_CAMK_Trbl | Pkinase, 104 338 * |
| ENSG00000173517 | ENSP00000309230 | 1746 | SgK269_Mm_Other_NKF3 | Pkinase, 1345 1663 * |
| ENSG00000173846 | ENSP00000361275 | 646 | PLK3_Mm_Other_PLK | Pkinase, 62 314 * POLO_box, 470 533 * POLO_box, 567 637 * |
| ENSG00000174292 | ENSP00000312309 | 666 | TNK1_Hs_TK_Ack | Pkinase, 116 377 * |
| ENSG00000174672 | ENSP00000310697 | 668 | BRSK2_Hs_CAMK_CAMKL | Pkinase, 19 270 * |
| ENSG00000174672 | ENSP00000310805 | 696 | BRSK2_Hs_CAMK_CAMKL | Pkinase, 19 270 * |
| ENSG00000174672 | ENSP00000371614 | 766 | BRSK2_Hs_CAMK_CAMKL | Pkinase, 77 316 * |
| ENSG00000175087 | ENSP00000363387 | 341 | CLIK1L_Hs_Other_NKF4 | Pkinase, 8 329 * |
| ENSG00000175634 | ENSP00000308413 | 482 | p70S6Kb_Mm_AGC_RSK | Pkinase, 67 328 * Pkinase_C, 348 393 * |
| ENSG00000176105 | ENSP00000352892 | 543 | YES_Mm_TK_Src | SH3_1, 94 150 * SH3_2, 95 150 * SH2, 158 240 * Pkinase, 277 526 * |
| ENSG00000176444 | ENSP00000347759 | 497 | CLK2_Mm_CMGC_CLK | Pkinase, 161 477 * |
| ENSG00000176444 | ENSP00000354856 | 498 | CLK2_Mm_CMGC_CLK | Pkinase, 162 478 * |
| ENSG00000176444 | ENSP00000357345 | 499 | CLK2_Mm_CMGC_CLK | Pkinase, 163 479 * |
| ENSG00000176444 | ENSP00000384480 | 271 | CLK2_Mm_CMGC_CLK | Pkinase, 1 251 * |
| ENSG00000176601 | ENSP00000351140 | 1215 | MAP3K8_Mm_STE_STE11 | Pkinase, 948 1211 * |
| ENSG00000176601 | ENSP00000365004 | 510 | MAP3K8_Mm_STE_STE11 | Pkinase, 243 506 * |
| ENSG00000176601 | ENSP00000365005 | 1328 | MAP3K8_Mm_STE_STE11 | Pkinase, 1061 1324 * |
| ENSG00000176601 | ENSP00000376650 | 462 | MAP3K8_Mm_STE_STE11 | Pkinase, 215 458 * |
| ENSG00000177169 | ENSP00000324560 | 1050 | ULK1_Mm_Other_ULK | Pkinase, 16 278 * |
| ENSG00000177189 | ENSP00000368865 | 227 | RSK1_Mm_AGC_RSK | Pkinase, 39 227 * |
| ENSG00000177189 | ENSP00000368884 | 740 | RSK1_Mm_AGC_RSK | Pkinase, 68 327 * Pkinase_C, 347 391 * Pkinase, 422 679 * |
| ENSG00000177453 | ENSP00000313572 | 436 | NIM1_Mm_CAMK_CAMKL | Pkinase, 74 325 * |
| ENSG00000177602 | ENSP00000325290 | 798 | Haspin_Mm_Other_Haspin | Pkinase, 484 797 * |
| ENSG00000178093 | ENSP00000354168 | 273 | SSTK_Mm_CAMK_TSSK | Pkinase, 12 267 * |
| ENSG00000178568 | ENSP00000260943 | 1292 | ErbB4_Mm_TK_EGFR | Recep_L_domain, 55 167 * Furin-like, 183 335 * Recep_L_domain, 358 478 * Pkinase, 718 974 * |
| ENSG00000178568 | ENSP00000342235 | 1308 | ErbB4_Mm_TK_EGFR | Recep_L_domain, 55 167 * Furin-like, 183 335 * Recep_L_domain, 358 478 * Pkinase, 718 974 * |
| ENSG00000178568 | ENSP00000385565 | 1298 | ErbB4_Mm_TK_EGFR | Recep_L_domain, 55 167 * Furin-like, 183 335 * Recep_L_domain, 358 478 * Pkinase, 708 964 * |
| ENSG00000178607 | ENSP00000225768 | 976 | IRE1_Mm_Other_IRE | PQQ, 30 67 * PQQ, 194 231 * Pkinase, 570 831 * Ribonuc_2-5A, 836 962 * |
| ENSG00000178950 | ENSP00000314499 | 1311 | GAK_Mm_Other_NAK | Pkinase, 40 313 * PTEN_C2, 570 709 * |
| ENSG00000178999 | ENSP00000313950 | 344 | AurC_Mm_Other_AUR | Pkinase, 77 327 * |
| ENSG00000179335 | ENSP00000328452 | 566 | CLK2_Mm_CMGC_CLK | Pkinase, 232 548 * |
| ENSG00000179335 | ENSP00000344112 | 490 | CLK2_Mm_CMGC_CLK | Pkinase, 156 472 * |
| ENSG00000179335 | ENSP00000323106 | 467 | CLK2_Mm_CMGC_CLK | Pkinase, 142 449 * |
| ENSG00000180138 | ENSP00000369126 | 337 | C03C10.1_Ce_CK1_CK1 | Pkinase, 17 281 * |
| ENSG00000180370 | ENSP00000314067 | 524 | PAK3_Mm_STE_STE20 | PBD, 73 131 * Pkinase, 249 500 * APH, 251 427 * |
| ENSG00000180815 | ENSP00000345629 | 1313 | MAP3K5_Mm_STE_STE11 | Pkinase, 653 908 * |
| ENSG00000180815 | ENSP00000352093 | 748 | MAP3K5_Mm_STE_STE11 | Pkinase, 88 343 * |
| ENSG00000180815 | ENSP00000374000 | 788 | MAP3K5_Mm_STE_STE11 | Pkinase, 128 383 * |
| ENSG00000181085 | ENSP00000313643 | 561 | ERK7_Mm_CMGC_MAPK | Pkinase, 13 321 * |
| ENSG00000181085 | ENSP00000337691 | 544 | ERK7_Mm_CMGC_MAPK | Pkinase, 13 304 * |
| ENSG00000181085 | ENSP00000378540 | 254 | ERK7_Mm_CMGC_MAPK | Pkinase, 13 249 * |
| ENSG00000181409 | ENSP00000324196 | 1374 | LMR1_Mm_TK_Lmr | Pkinase, 125 395 * |
| ENSG00000181409 | ENSP00000363924 | 879 | LMR1_Mm_TK_Lmr | Pkinase, 125 395 * |
| ENSG00000182319 | ENSP00000330930 | 1402 | SgK223_Hs_Other_NKF3 | Pkinase, 992 1321 * |
| ENSG00000182511 | ENSP00000331504 | 822 | FES_Mm_TK_Fer | FCH, 1 94 * SH2, 460 530 * Pkinase, 561 814 * |
| ENSG00000182511 | ENSP00000377837 | 764 | FES_Mm_TK_Fer | FCH, 1 98 * SH2, 402 472 * Pkinase, 503 756 * |
| ENSG00000182511 | ENSP00000377839 | 681 | FES_Mm_TK_Fer | FCH, 1 98 * Pkinase, 433 673 * |
| ENSG00000182511 | ENSP00000377841 | 752 | FES_Mm_TK_Fer | FCH, 1 94 * Pkinase, 491 744 * |
| ENSG00000182541 | ENSP00000332687 | 638 | LIMK2_Mm_TKL_LISK | LIM, 12 68 * LIM, 72 129 * PDZ, 152 236 * Kdo, 319 511 * Pkinase, 331 601 * |
| ENSG00000182541 | ENSP00000330470 | 617 | LIMK2_Mm_TKL_LISK | LIM, 6 47 * LIM, 51 108 * PDZ, 131 215 * Kdo, 298 490 * Pkinase, 310 580 * |
| ENSG00000182541 | ENSP00000339916 | 686 | LIMK2_Mm_TKL_LISK | LIM, 6 47 * LIM, 51 108 * PDZ, 131 215 * Kdo, 298 490 * Pkinase, 310 608 * PP1_inhibitor, 558 686 * |
| ENSG00000182541 | ENSP00000384602 | 629 | LIMK2_Mm_TKL_LISK | LIM, 2 51 * PDZ, 74 158 * Kdo, 241 433 * Pkinase, 253 551 * PP1_inhibitor, 501 629 * |
| ENSG00000182578 | ENSP00000286301 | 972 | FMS_Hs_TK_PDGFR | V-set, 19 102 * ig, 35 86 * V-set, 202 296 * ig, 217 280 * ig, 412 487 * Pkinase, 582 910 * |
| ENSG00000182580 | ENSP00000332118 | 998 | EphB4_Mm_TK_Eph | Ephrin_lbd, 39 212 * GCC2_GCC3, 274 320 * fn3, 340 435 * fn3, 453 535 * Pkinase, 633 892 * SAM_2, 922 989 * SAM_1, 923 987 * |
| ENSG00000182866 | ENSP00000362663 | 539 | LCK_Hs_TK_Src | SH3_1, 64 119 * SH3_2, 65 119 * SH2, 127 209 * Pkinase, 245 527 * |
| ENSG00000182866 | ENSP00000362665 | 516 | LCK_Hs_TK_Src | SH3_1, 122 177 * SH3_2, 123 177 * SH2, 185 267 * Pkinase, 210 504 * |
| ENSG00000182866 | ENSP00000381387 | 509 | LCK_Hs_TK_Src | SH3_1, 64 119 * SH3_2, 65 119 * SH2, 127 209 * Pkinase, 245 497 * |
| ENSG00000183049 | ENSP00000368122 | 357 | CaMK1a_Hs_CAMK_CAMK1 | Pkinase, 23 279 * |
| ENSG00000183049 | ENSP00000368124 | 385 | CaMK1a_Hs_CAMK_CAMK1 | Pkinase, 23 279 * |
| ENSG00000183421 | ENSP00000332454 | 784 | ANKRD3_Mm_TKL_RIPK | Pkinase, 22 282 * Ank, 437 469 * Ank, 470 502 * Ank, 503 535 * Ank, 536 568 * Ank, 569 602 * Ank, 603 635 * Ank, 636 668 * Ank, 669 701 * Ank, 702 732 * Ank, 734 766 * |
| ENSG00000183421 | ENSP00000330161 | 832 | ANKRD3_Mm_TKL_RIPK | Pkinase, 22 293 * Ank, 485 517 * Ank, 518 550 * Ank, 551 583 * Ank, 584 616 * Ank, 617 650 * Ank, 651 683 * Ank, 684 716 * Ank, 717 749 * Ank, 750 780 * Ank, 782 814 * |
| ENSG00000183735 | ENSP00000329967 | 729 | IKKe_Mm_Other_IKK | Pkinase, 9 301 * |
| ENSG00000183765 | ENSP00000372021 | 452 | CHK2_Mm_CAMK_RAD53 | Pkinase, 129 395 * |
| ENSG00000183765 | ENSP00000372023 | 586 | CHK2_Mm_CAMK_RAD53 | FHA, 156 235 * Pkinase, 263 529 * |
| ENSG00000183765 | ENSP00000386087 | 543 | CHK2_Mm_CAMK_RAD53 | FHA, 113 192 * Pkinase, 220 486 * |
| ENSG00000183943 | ENSP00000262848 | 358 | Pka-C1_Dm_AGC_PKA | Pkinase, 49 303 * |
| ENSG00000184216 | ENSP00000358990 | 708 | IRAK1_Mm_TKL_IRAK | Death, 55 129 * Pkinase, 238 544 * |
| ENSG00000184216 | ENSP00000358991 | 633 | IRAK1_Mm_TKL_IRAK | Death, 28 103 * Pkinase, 212 540 * |
| ENSG00000184216 | ENSP00000358997 | 712 | IRAK1_Mm_TKL_IRAK | Death, 28 103 * Pkinase, 212 518 * |
| ENSG00000184216 | ENSP00000377287 | 693 | IRAK1_Mm_TKL_IRAK | Death, 55 129 * Pkinase, 238 529 * |
| ENSG00000184216 | ENSP00000377291 | 682 | IRAK1_Mm_TKL_IRAK | Death, 28 103 * Pkinase, 212 518 * |
| ENSG00000184304 | ENSP00000333568 | 912 | PKD3_Hs_CAMK_PKD | C1_1, 147 199 * C1_3, 158 189 * C1_1, 271 323 * C1_3, 282 313 * PH, 423 541 * Pkinase, 583 839 * |
| ENSG00000184304 | ENSP00000374634 | 875 | PKD3_Hs_CAMK_PKD | C1_1, 147 199 * C1_3, 158 189 * C1_1, 271 323 * C1_3, 282 313 * PH, 423 541 * Pkinase, 583 802 * |
| ENSG00000184304 | ENSP00000374635 | 912 | PKD3_Hs_CAMK_PKD | C1_1, 147 199 * C1_3, 158 189 * C1_1, 271 323 * C1_3, 282 313 * PH, 423 541 * Pkinase, 583 839 * |
| ENSG00000184304 | ENSP00000379907 | 849 | PKD3_Hs_CAMK_PKD | C1_1, 147 199 * C1_1, 271 320 * PH, 397 515 * Pkinase, 557 776 * |
| ENSG00000184343 | ENSP00000359118 | 492 | MSSK1_Hs_CMGC_SRPK | Pkinase, 37 490 * |
| ENSG00000184343 | ENSP00000359119 | 567 | MSSK1_Mm_CMGC_SRPK | Pkinase, 79 565 * |
| ENSG00000184343 | ENSP00000359122 | 566 | MSSK1_Mm_CMGC_SRPK | Pkinase, 79 564 * |
| ENSG00000184343 | ENSP00000359126 | 534 | MSSK1_Hs_CMGC_SRPK | Pkinase, 79 532 * |
| ENSG00000184343 | ENSP00000377376 | 533 | MSSK1_Hs_CMGC_SRPK | Pkinase, 79 531 * |
| ENSG00000185324 | ENSP00000329957 | 314 | CDK10_Mm_CMGC_CDK | Pkinase, 10 294 * |
| ENSG00000185324 | ENSP00000338673 | 360 | CDK10_Mm_CMGC_CDK | Pkinase, 39 323 * |
| ENSG00000185386 | ENSP00000333685 | 364 | p38b_Mm_CMGC_MAPK | Pkinase, 24 308 * |
| ENSG00000185483 | ENSP00000360120 | 937 | ROR1_Mm_TK_Ror | V-set, 37 150 * I-set, 58 148 * ig, 72 133 * Fz, 164 297 * Kringle, 313 391 * Pkinase, 473 746 * |
| ENSG00000185532 | ENSP00000363086 | 283 | PKG1_Hs_AGC_PKG | Pkinase, 1 231 * |
| ENSG00000185532 | ENSP00000363092 | 686 | PKG1_Hs_AGC_PKG | cNMP_binding, 136 221 * cNMP_binding, 254 345 * Pkinase, 375 634 * |
| ENSG00000185532 | ENSP00000384200 | 671 | PKG1_Hs_AGC_PKG | cNMP_binding, 121 206 * cNMP_binding, 239 330 * Pkinase, 360 619 * |
| ENSG00000187550 | ENSP00000345044 | 348 | NKF1_Sp_Other_NKF1 | Pkinase, 62 325 * |
| ENSG00000188130 | ENSP00000215659 | 367 | p38a_Mm_CMGC_MAPK | Pkinase, 27 311 * |
| ENSG00000188130 | ENSP00000379126 | 277 | p38a_Mm_CMGC_MAPK | Pkinase, 1 221 * |
| ENSG00000188322 | ENSP00000343248 | 424 | SBK_Mm_Other_NKF1 | Pkinase, 53 313 * |
| ENSG00000188906 | ENSP00000298910 | 2527 | LRRK2_Hs_TKL_LRRK | LRR_1, 983 1004 * LRR_1, 1012 1034 * LRR_1, 1036 1057 * LRR_1, 1084 1105 * LRR_1, 1108 1128 * LRR_1, 1130 1152 * LRR_1, 1174 1195 * LRR_1, 1197 1217 * LRR_1, 1221 1237 * LRR_1, 1246 1267 * LRR_1, 1269 1291 * MMR_HSR1, 1335 1455 * Miro, 1336 1455 * Ras, 1336 1510 * Pkinase, 1879 2132 * |
| ENSG00000189229 | ENSP00000345856 | 785 | PITSLRE_Mm_CMGC_CDK | Pkinase, 428 713 * |
| ENSG00000189229 | ENSP00000341389 | 738 | PITSLRE_Mm_CMGC_CDK | Pkinase, 381 666 * |
| ENSG00000189229 | ENSP00000349411 | 772 | PITSLRE_Mm_CMGC_CDK | Pkinase, 415 700 * |
| ENSG00000189229 | ENSP00000385067 | 439 | PITSLRE_Mm_CMGC_CDK | Pkinase, 82 367 * |
| ENSG00000196182 | ENSP00000352245 | 417 | Snf1_Dd_CAMK_CAMKL | Pkinase, 35 328 * |
| ENSG00000196182 | ENSP00000362222 | 440 | Snf1_Dd_CAMK_CAMKL | Pkinase, 40 333 * |
| ENSG00000196182 | ENSP00000362224 | 435 | Snf1_Dd_CAMK_CAMKL | Pkinase, 35 328 * |
| ENSG00000196411 | ENSP00000350896 | 987 | EphB4_Mm_TK_Eph | Ephrin_lbd, 17 197 * NCD3G, 248 294 * GCC2_GCC3, 258 302 * fn3, 324 414 * fn3, 434 519 * Pkinase, 615 874 * SAM_2, 904 971 * SAM_1, 905 969 * |
| ENSG00000196411 | ENSP00000353833 | 935 | EphB4_Mm_TK_Eph | Ephrin_lbd, 17 197 * NCD3G, 248 294 * GCC2_GCC3, 258 302 * fn3, 324 414 * fn3, 434 519 * Pkinase, 615 874 * SAM_1, 863 917 * |
| ENSG00000196455 | ENSP00000349205 | 1358 | PIK3R4_Mm_Other_VPS15 | Pkinase, 26 310 * HEAT, 412 448 * HEAT, 457 493 * HEAT, 571 608 * HEAT, 614 646 * HEAT, 651 686 * WD40, 983 1021 * WD40, 1032 1070 * WD40, 1133 1169 * WD40, 1229 1269 * WD40, 1319 1358 * |
| ENSG00000196632 | ENSP00000346667 | 1800 | Wnk3_Mm_Other_WNK | Pkinase, 147 405 * |
| ENSG00000196632 | ENSP00000351601 | 1790 | Wnk3_Mm_Other_WNK | Pkinase, 147 405 * |
| ENSG00000196632 | ENSP00000364312 | 1743 | Wnk3_Mm_Other_WNK | Pkinase, 147 405 * |
| ENSG00000196730 | ENSP00000350785 | 1455 | DAPK1_Mm_CAMK_DAPK | Pkinase, 13 275 * Ank, 378 410 * Ank, 411 443 * Ank, 444 476 * Ank, 477 509 * Ank, 510 542 * Ank, 543 575 * Ank, 576 608 * Ank, 609 641 * Death, 1336 1421 * |
| ENSG00000196730 | ENSP00000386135 | 1430 | DAPK1_Mm_CAMK_DAPK | Pkinase, 13 275 * Ank, 378 410 * Ank, 411 443 * Ank, 444 476 * Ank, 477 509 * Ank, 510 542 * Ank, 543 575 * Ank, 576 608 * Ank, 609 641 * Death, 1311 1396 * |
| ENSG00000197122 | ENSP00000350941 | 536 | SRC_Mm_TK_Src | SH3_1, 87 143 * SH3_2, 88 143 * SH2, 151 233 * Pkinase, 270 519 * |
| ENSG00000197122 | ENSP00000353950 | 542 | SRC_Mm_TK_Src | SH3_1, 87 149 * SH3_2, 88 149 * SH2, 157 239 * Pkinase, 276 525 * |
| ENSG00000197168 | ENSP00000347767 | 708 | 28433_Tt_Other_NEK | Pkinase, 4 259 * |
| ENSG00000197442 | ENSP00000348104 | 1374 | MAP3K5_Mm_STE_STE11 | Pkinase, 675 938 * |
| ENSG00000197442 | ENSP00000351908 | 1374 | MAP3K5_Mm_STE_STE11 | Pkinase, 675 938 * |
| ENSG00000197442 | ENSP00000356742 | 1256 | MAP3K5_Mm_STE_STE11 | Pkinase, 755 1018 * |
| ENSG00000198001 | ENSP00000349096 | 460 | IRAK4_Mm_TKL_IRAK | Pkinase, 186 454 * |
| ENSG00000198055 | ENSP00000347655 | 576 | GPRK6_Hs_AGC_GRK | RGS, 52 170 * Pkinase, 186 448 * |
| ENSG00000198055 | ENSP00000377204 | 589 | GPRK6_Hs_AGC_GRK | RGS, 52 170 * Pkinase, 186 448 * |
| ENSG00000198208 | ENSP00000346644 | 541 | RSKL2_Hs_AGC_RSKL | MIT, 49 117 * Pkinase, 116 508 * |
| ENSG00000198208 | ENSP00000351086 | 572 | RSKL2_Hs_AGC_RSKL | MIT, 49 117 * Pkinase, 153 539 * |
| ENSG00000198355 | ENSP00000353824 | 326 | PIM3_Mm_CAMK_PIM | Kdo, 34 222 * Pkinase, 40 293 * |
| ENSG00000198400 | ENSP00000351486 | 796 | TRKA_Mm_TK_Trk | LRR_1, 67 90 * LRR_1, 92 114 * LRR_1, 116 138 * LRR_1, 139 162 * I-set, 194 282 * Pkinase, 510 781 * |
| ENSG00000198400 | ENSP00000357179 | 790 | TRKA_Mm_TK_Trk | LRR_1, 67 90 * LRR_1, 92 114 * LRR_1, 116 138 * LRR_1, 139 162 * I-set, 194 282 * Pkinase, 504 775 * |
| ENSG00000198400 | ENSP00000357180 | 760 | TRKA_Mm_TK_Trk | I-set, 164 252 * Pkinase, 474 745 * |
| ENSG00000198400 | ENSP00000376120 | 760 | TRKA_Mm_TK_Trk | I-set, 164 252 * Pkinase, 474 745 * |
| ENSG00000198586 | ENSP00000352810 | 766 | TLK1_Hs_Other_TLK | Pkinase, 456 734 * |
| ENSG00000198586 | ENSP00000354089 | 787 | TLK1_Hs_Other_TLK | Pkinase, 477 755 * |
| ENSG00000198586 | ENSP00000387313 | 670 | TLK1_Hs_Other_TLK | Pkinase, 360 638 * |
| ENSG00000198648 | ENSP00000348278 | 545 | OSR1_Mm_STE_STE20 | Pkinase, 63 337 * |
| ENSG00000198752 | ENSP00000355237 | 1711 | MRCKb_Hs_AGC_DMPK | Pkinase, 76 342 * Pkinase_C, 360 407 * M, 452 472 * M, 575 595 * M, 624 644 * M, 771 791 * M, 792 812 * DMPK_coil, 878 939 * M, 909 929 * C1_1, 1026 1078 * PH, 1096 1214 * CNH, 1241 1513 * |
| ENSG00000198873 | ENSP00000358104 | 636 | GPRK5_Hs_AGC_GRK | RGS, 98 216 * Pkinase, 232 494 * |
| ENSG00000198873 | ENSP00000376609 | 590 | GPRK5_Hs_AGC_GRK | RGS, 52 170 * Pkinase, 186 448 * |
| ENSG00000198909 | ENSP00000354927 | 657 | MAP3K3_Hs_STE_STE11 | PB1, 75 154 * Pkinase, 393 653 * |
| ENSG00000198909 | ENSP00000354485 | 626 | MAP3K3_Hs_STE_STE11 | PB1, 44 123 * Pkinase, 362 622 * |
| ENSG00000204217 | ENSP00000363702 | 530 | BMPR2_Mm_TKL_STKR | Activin_recp, 32 131 * Pkinase, 203 501 * |
| ENSG00000204217 | ENSP00000363708 | 1038 | BMPR2_Mm_TKL_STKR | Activin_recp, 32 131 * Pkinase, 203 501 * |
| ENSG00000205111 | ENSP00000368080 | 315 | CDKL4_Hs_CMGC_CDKL | Pkinase, 4 286 * |
| ENSG00000205111 | ENSP00000378476 | 379 | CDKL4_Hs_CMGC_CDKL | Pkinase, 4 286 * |
| ENSG00000205918 | ENSP00000371763 | 396 | PDK1_Mm_AGC_PDK1 | Pkinase, 55 315 * |
| ENSG00000206203 | ENSP00000382544 | 358 | TSSK2_Hs_CAMK_TSSK | Pkinase, 12 272 * |
| ENSG00000211455 | ENSP00000373684 | 464 | NDR2_Mm_AGC_NDR | Pkinase, 90 383 * Pkinase_C, 401 447 * |
| ENSG00000212122 | ENSP00000375081 | 367 | TSSK2_Hs_CAMK_TSSK | Pkinase, 12 272 * |
| ENSG00000213341 | ENSP00000359424 | 745 | IKKa_Mm_Other_IKK | Pkinase, 15 307 * |
| ENSG00000213923 | ENSP00000381531 | 311 | C03C10.1_Ce_CK1_CK1 | Pkinase, 9 273 * |
| ENSG00000213923 | ENSP00000385582 | 416 | C03C10.1_Ce_CK1_CK1 | Pkinase, 9 273 * |
| ENSG00000213923 | ENSP00000384426 | 336 | C03C10.1_Ce_CK1_CK1 | Pkinase, 9 273 * |
| ENSG00000214102 | ENSP00000380675 | 567 | Wee1B_Hs_Other_WEE | Pkinase, 212 486 * |
| ENSG00000215522 | ENSP00000383339 | 913 | DDR1_Mm_TK_DDR | F5_F8_type_C, 46 182 * Pkinase, 610 913 * |
