# Supplementary material for: Functional diversity of human protein kinase splice variants marks significant expansion of human kinome
Source: BMC Genomics. 2009 Dec 22;10:622. doi: 10.1186/1471-2164-10-622 (PMC2805699; doi:10.1186/1471-2164-10-622)
Supplement: Additional file 2 — List of human protein kinase splice variants with different domain organisations. [file 1471-2164-10-622-S2.DOC]

**Additional file 2:** List of human protein kinase splice variants with different domain organisations. Gene accession code, protein accession code, number of residues, subfamily classification and domain architecture are also provided. Abbreviations followed are: CAMK, Calcium/Calmodulin dependent protein kinase; CAMKL, Calcium/Calmodulin dependent protein kinase like; Pkinase, protein kinase; UBA, Ubiquitin associated domain; KA1, Kinase associated domain 1; PH, Pleckstrin homology; BTK, Bruton's tyrosine kinase; SH2, Src homology2; SH3, Src homology3; TK, Tyrosine protein kinase; I-set, immunoglobulin; RhoGEF, Guanine nucleotide exchange factor for Rho/Rac/Cdc42-like GTPases; MLCK, Myosin light chain kinase; fn3, Fibronectin3; V-set, Immunoglobulin; DAPK, Death associated protein kinase; PKC, Protein kinase C; AGC, Protein kinase A protein kinase G Protein kinase C; EGF, Epidermal growth factor; ig, Immunoglobulin; FGFR, Fibroblast growth factor receptor; PB1, Phox and Bem1p; VEGFR, Vascular endothelial growth factor receptor; DUF, Domain of unknown function; MAST, Microtubule associated serine/threonine kinase; TKL, Tyrosine kinase like; MLK, Mixed lineage kinase; SAM, Sterile alpha motif; Activin_recp, Activin receptor; STKR, Serine/threonine kinase receptor; TGF_beta_GS, Transforming growth factor beta type I GS-motif; CCK4, Colon carcinoma kinase 4; FAK, Focal adhesion kinase; Focal_AT, Focal adhesion targeting region; TTBK, Tau tubulin kinase; CK1, Casein kinase1; CaMKII_AD, Calcium/calmodulin dependent protein kinase II association domain; Guanylate_kin, Guanylate kinase; L27, Lin-2 and Lin-7; CASK, Calcium/calmodulin-dependent serine protein kinase; RRM_1, RNA recognition motif; KIS, kinase interacting with stathmin; Ror, Regeneron orphan receptor; Fz, Frizzled; NEK, NimA kinase; PLK, Polo like kinase; RSK, Ribosomal S6 kinase; FCH, Fes/CIP4 homology; LIM, LIN-11, Isl1 and MEC-3; PP1_inhibitor, PKC-activated protein phosphatase-1 inhibitor; FHA, Forkhead-associated; C1, Phorbol esters/diacylglycerol binding; PKG, Protein kinase G; cNMP_binding, cyclic nucleotide monophosphate binding; PKD, Protein kinase D; LRR, Leucine rich repeat.

| Gene accession code | Protein accession code | Number of residues | Protein kinase subfamily | Domain name and domain boundary |
| --- | --- | --- | --- | --- |
| ENSG00000007047 | ENSP00000262891 | 752 | MARK1_Hs_CAMK_CAMKL | Pkinase, 59 310 * UBA, 330 366 * KA1, 706 752 * |
| ENSG00000007047 | ENSP00000262893 | 462 | MARK2_Hs_CAMK_CAMKL | Pkinase, 59 340 * UBA, 360 396 * |
| ENSG00000007047 | ENSP00000300843 | 688 | MARK2_Hs_CAMK_CAMKL | Pkinase, 59 310 * UBA, 330 366 * |
|  |  |  |  |  |
| ENSG00000010671 | ENSP00000308176 | 659 | BTK_Mm_TK_Tec | PH, 4 133 * BTK, 140 171 * SH3, 217 272 * SH2, 281 362 * Pkinase, 402 651 * |
| ENSG00000010671 | ENSP00000361946 | 252 | BTK_Mm_TK_Tec | Pkinase, 7 244 * |
|  |  |  |  |  |
| ENSG00000010810 | ENSP00000346671 | 485 | FYN_Hs_TK_Src | SH2, 100 182 * Pkinase, 219 468 * |
| ENSG00000010810 | ENSP00000348295 | 482 | FYN_Hs_TK_Src | SH3, 85 141 * SH2, 149 231 * Pkinase, 221 465* |
| ENSG00000010810 | ENSP00000357656 | 537 | FYN_Hs_TK_Src | SH3, 85 141 * SH2, 149 231 * Pkinase, 271 520* |
| ENSG00000010810 | ENSP00000357671 | 534 | FYN_Hs_TK_Src | SH3, 85 141 * SH2, 149 231 * Pkinase, 268 517* |
|  |  |  |  |  |
| ENSG00000038382 | ENSP00000339291 | 596 | Trio_Hs_CAMK_Trio | I-set, 184 275 * Pkinase, 295 549 * |
| ENSG00000038382 | ENSP00000339299 | 3097 | Trio_Hs_CAMK_Trio | Spectrin, 218 338 * Spectrin, 340 446 * Spectrin, 566 670 * Spectrin, 671 784 * Spectrin, 907 1012 * Spectrin, 1138 1244 * RhoGEF, 1296 1466 * PH, 1480 1591 * SH3, 1659 1719 * RhoGEF, 1973 2144 * PH, 2158 2271 * I-set, 2685 2776 * Pkinase, 2796 3050 * |
|  |  |  |  |  |
| ENSG00000065534 | ENSP00000320622 | 1845 | smMLCK_Hs_CAMK_MLCK | I-set, 33 123 * I-set, 161 250 * I-set, 445 531 * I-set, 554 643 * I-set, 652 742 * I-set, 1029 1118 * V-set, 1168 1259 * fn3, 1262 1347 * Pkinase, 1395 1650 * I-set, 1740 1830 * |
| ENSG00000065534 | ENSP00000346846 | 1845 | smMLCK_Hs_CAMK_MLCK | I-set, 33 123 * I-set, 161 250 * I-set, 445 531 * I-set, 554 643 * I-set, 652 742 * I-set, 1029 1118 * V-set, 1168 1259 * fn3, 1262 1347 * Pkinase, 1395 1650 * I-set, 1740 1830 * |
| ENSG00000065534 | ENSP00000352088 | 1863 | smMLCK_Hs_CAMK_MLCK | I-set, 33 123 * I-set, 161 250 * V-set, 349 505 * I-set, 514 600 * I-set, 623 712 * I-set, 721 811 * I-set, 1098 1187 * V-set, 1237 1328 * fn3, 1331 1416 * Pkinase, 1464 1668 * I-set, 1758 1848 * |
| ENSG00000065534 | ENSP00000353452 | 1914 | smMLCK_Hs_CAMK_MLCK | I-set, 33 123 * I-set, 161 250 * V-set, 349 505 * I-set, 514 600 * I-set, 623 712 * I-set, 721 811 * I-set, 1098 1187 * V-set, 1237 1328 * fn3, 1331 1416 * Pkinase, 1464 1719 * I-set, 1809 1899 * |
| ENSG00000065534 | ENSP00000353530 | 1841 | DRAK_Sp_CAMK_DAPK | I-set, 33 123 * I-set, 161 250 * V-set, 349 505 * I-set, 514 600 * I-set, 623 712 * I-set, 721 811 * I-set, 1098 1187 * V-set, 1237 1328 * fn3, 1331 1416 * Pkinase, 1421 1646 * I-set, 1736 1826 * |
| ENSG00000065534 | ENSP00000355024 | 992 | smMLCK_Hs_CAMK_MLCK | I-set, 176 265 * V-set, 315 406 * fn3, 409 494 * Pkinase, 542 797 * I-set, 887 977 * |
|  |  |  |  |  |
| ENSG00000065675 | ENSP00000380360 | 478 | PKCt_Mm_AGC_PKC | Pkinase, 152 406 * Pkinase_C, 426 472 * |
| ENSG00000065675 | ENSP00000380361 | 643 | AktA_Sp_AGC_Akt | I-set, 176 265 * V-set, 315 406 * fn3, 409 494 * Pkinase, 542 797 * I-set, 887 977 * |
| ENSG00000065675 | ENSP00000380363 | 706 | PKCt_Mm_AGC_PKC | C1, 160 212 * C1, 232 284 * Pkinase, 380 634 * Pkinase_C, 654 700 * |
|  |  |  |  |  |
| ENSG00000066056 | ENSP00000361553 | 541 | TIE1_Mm_TK_Tie | fn3, 47 132 * Pkinase, 242 510 * |
| ENSG00000066056 | ENSP00000361554 | 1138 | TIE1_Mm_TK_Tie | ig, 139 197 * EGF, 224 255 * EGF, 315 344 * ig, 365 428 * fn3, 446 533 * fn3, 546 632 * fn3, 644 729 * Pkinase, 839 1107 * |
|  |  |  |  |  |
| ENSG00000066468 | ENSP00000263451 | 819 | FGFR2_Mm_TK_FGFR | V-set, 41 125 * V-set, 159 249 * I-set, 256 359 * Pkinase, 479 755 * |
| ENSG00000066468 | ENSP00000263454 | 731 | FGFR2_Mm_TK_FGFR | V-set, 70 160 * I-set, 167 268 * Pkinase, 391 667* |
| ENSG00000066468 | ENSP00000309878 | 841 | FGFR2_Mm_TK_FGFR | V-set, 60 144 * V-set, 178 268 * I-set, 275 376 * Pkinase, 501 777 * |
| ENSG00000066468 | ENSP00000337665 | 785 | FGFR2_Mm_TK_FGFR | V-set, 41 125 * V-set, 159 249 * I-set, 256 359 * Pkinase, 479 755 * |
| ENSG00000066468 | ENSP00000348559 | 704 | FGFR2_Mm_TK_FGFR | V-set, 44 134 * I-set, 141 244 * Pkinase, 364 640* |
| ENSG00000066468 | ENSP00000350166 | 840 | FGFR2_Mm_TK_FGFR | V-set, 60 144 * V-set, 178 268 * I-set, 275 378 * Pkinase, 500 776 * |
| ENSG00000066468 | ENSP00000353262 | 680 | FGFR2_Mm_TK_FGFR | V-set, 70 160 * I-set, 167 268 * Pkinase, 393 669* |
| ENSG00000066468 | ENSP00000358054 | 772 | FGFR2_Mm_TK_FGFR | V-set, 41 125 * V-set, 159 249 * I-set, 256 357 * Pkinase, 482 758 * |
| ENSG00000066468 | ENSP00000358055 | 707 | FGFR2_Mm_TK_FGFR | V-set, 44 134 * I-set, 141 242 * Pkinase, 367 643 * |
| ENSG00000066468 | ENSP00000358056 | 705 | FGFR2_Mm_TK_FGFR | V-set, 41 125 * V-set, 159 249 * Pkinase, 365 641* |
| ENSG00000066468 | ENSP00000358057 | 709 | FGFR2_Mm_TK_FGFR | V-set, 41 125 * V-set, 159 249 * Pkinase, 369 645* |
| ENSG00000066468 | ENSP00000358058 | 822 | FGFR2_Mm_TK_FGFR | V-set, 41 125 * V-set, 159 249 * I-set, 256 362 * Pkinase, 482 758* |
|  |  |  |  |  |
| ENSG00000067606 | ENSP00000367830 | 592 | PKCz_Hs_AGC_PKC | PB1, 15 98 * C1_1, 131 183 * Pkinase, 252 518 * Pkinase_C, 538 584 * |
| ENSG00000067606 | ENSP00000383712 | 409 | PKCz_Hs_AGC_PKC | Pkinase, 69 335 * Pkinase_C, 355 401 * |
|  |  |  |  |  |
| ENSG00000068078 | ENSP00000231803 | 694 | FGFR3_Mm_TK_FGFR | ig, 54 111 * V-set, 122 246 * Pkinase, 360 636 * |
| ENSG00000068078 | ENSP00000260795 | 806 | FGFR3_Mm_TK_FGFR | ig, 54 111 * V-set, 122 246 * I-set, 253 356 * Pkinase, 472 748 * |
| ENSG00000068078 | ENSP00000339824 | 808 | FGFR3_Mm_TK_FGFR | ig, 54 111 * V-set, 122 246 * I-set, 253 355 * Pkinase, 474 750 * |
| ENSG00000068078 | ENSP00000354170 | 879 | FLT4_Mm_TK_VEGFR | ig, 141 198 * V-set, 209 333 * I-set, 340 443 * Pkinase, 560 875 * |
|  |  |  |  |  |
| ENSG00000071909 | ENSP00000314213 | 1277 | MYO3B_Mm_STE_STE20 | Pkinase, 26 292 * Myosin_head, 344 1045 * IQ, 1060 1080 * IQ, 1087 1107 * |
| ENSG00000071909 | ENSP00000314650 | 1250 | MYO3B_Mm_STE_STE20 | Pkinase, 26 292 * Myosin_head, 344 1045 * |
| ENSG00000071909 | ENSP00000335100 | 1274 | MYO3B_Mm_STE_STE20 | Pkinase, 26 292 * Myosin_head, 344 1045 * IQ, 1060 1080 * IQ, 1087 1107 * |
| ENSG00000071909 | ENSP00000386213 | 1341 | MYO3B_Mm_STE_STE20 | Pkinase, 27 293 * Myosin_head, 345 1046 * IQ, 1061 1081 * IQ, 1088 1108 * |
| ENSG00000071909 | ENSP00000386497 | 1314 | MYO3B_Mm_STE_STE20 | Pkinase, 27 293 * Myosin_head, 345 1046 * |
| ENSG00000071909 | ENSP00000386573 | 1191 | MYO3B_Mm_STE_STE20 | Pkinase, 27 293 * Myosin_head, 345 1045 * IQ, 1060 1080 * IQ, 1087 1107 * |
|  |  |  |  |  |
| ENSG00000077782 | ENSP00000327229 | 820 | FGFR2_Mm_TK_FGFR | I-set, 34 118 * V-set, 158 248 * I-set, 255 358 * Pkinase, 476 752 * |
| ENSG00000077782 | ENSP00000337247 | 812 | FGFR2_Mm_TK_FGFR | I-set, 26 110 * V-set, 150 240 * I-set, 247 350 * Pkinase, 468 744 * |
| ENSG00000077782 | ENSP00000340636 | 822 | FGFR2_Mm_TK_FGFR | I-set, 34 118 * V-set, 158 248 * I-set, 255 358 * Pkinase, 478 754 * |
| ENSG00000077782 | ENSP00000348537 | 733 | FGFR2_Mm_TK_FGFR | V-set, 69 159 * I-set, 166 269 * Pkinase, 389 665 * |
| ENSG00000077782 | ENSP00000380292 | 733 | FGFR2_Mm_TK_FGFR | V-set, 69 159 * I-set, 166 269 * Pkinase, 389 665 * |
| ENSG00000077782 | ENSP00000380297 | 731 | FGFR2_Mm_TK_FGFR | V-set, 67 157 * I-set, 164 267 * Pkinase, 387 663 * |
| ENSG00000077782 | ENSP00000380302 | 820 | FGFR2_Mm_TK_FGFR | I-set, 34 118 * V-set, 156 246 * I-set, 253 356 * Pkinase, 476 752 * |
|  |  |  |  |  |
| ENSG00000086015 | ENSP00000354671 | 1798 | MAST2_Mm_AGC_MAST | DUF1908, 199 476 * Pkinase, 512 785 * Pkinase_C, 803 848 * PDZ, 1104 1189 * |
| ENSG00000086015 | ENSP00000361078 | 792 | MAST2_Mm_AGC_MAST | DUF1908, 84 361 * Pkinase, 397 670 * Pkinase_C, 688 733 * |
| ENSG00000086015 | ENSP00000361079 | 1608 | MAST2_Mm_AGC_MAST | DUF1908, 199 406 * Pkinase, 442 715 * Pkinase_C, 733 778 * PDZ, 1009 1096 * |
|  |  |  |  |  |
| ENSG00000091436 | ENSP00000340257 | 455 | ZAK_Mm_TKL_MLK | Pkinase, 16 260 * |
| ENSG00000091436 | ENSP00000364361 | 800 | ZAK_Mm_TKL_MLK | Pkinase, 16 260 * SAM, 336 410 * |
| ENSG00000091436 | ENSP00000387259 | 800 | ZAK_Mm_TKL_MLK | Pkinase, 16 260 * SAM, 336 410 * |
|  |  |  |  |  |
| ENSG00000095777 | ENSP00000265944 | 1616 | MYO3A_Mm_STE_STE20 | Pkinase, 21 287 * Myosin_head, 340 1041 * IQ, 1056 1076 * IQ, 1083 1103 * IQ, 1347 1367 * |
| ENSG00000095777 | ENSP00000365478 | 197 | 7147_Tt_STE_STE11 | Pkinase, 21 197 * |
| ENSG00000095777 | ENSP00000365479 | 247 | MYO3A_Mm_STE_STE20 | Pkinase, 21 246 * |
| ENSG00000095777 | ENSP00000379679 | 664 | MYO3A_Mm_STE_STE20 | Pkinase, 21 287 * Myosin_head, 340 663 * |
|  |  |  |  |  |
| ENSG00000106799 | ENSP00000364129 | 426 | ALK4_Hs_TKL_STKR | Activin_recp, 34 114 * Pkinase, 128 415 * |
| ENSG00000106799 | ENSP00000364133 | 503 | ALK4_Hs_TKL_STKR | Activin_recp, 34 114 * TGF_beta_GS, 175 203 * Pkinase, 205 492 * |
|  |  |  |  |  |
| ENSG00000112655 | ENSP00000230419 | 1070 | CCK4_Mm_TK_CCK4 | I-set, 32 121 * I-set, 128 217 * I-set, 225 319 * I-set, 331 408 * I-set, 412 498 * I-set, 502 588 * V-set, 591 682 * Pkinase, 796 1061 * |
| ENSG00000112655 | ENSP00000324119 | 396 | CCK4_Mm_TK_CCK4 | I-set, 7 84 * Pkinase, 122 387 * |
| ENSG00000112655 | ENSP00000325462 | 940 | CCK4_Mm_TK_CCK4 | I-set, 32 121 * I-set, 128 217 * I-set, 225 319 * I-set, 331 408 * ig, 401 442 * V-set, 461 552 * Pkinase, 666 931 * |
| ENSG00000112655 | ENSP00000325992 | 1030 | CCK4_Mm_TK_CCK4 | I-set, 32 121 * I-set, 128 217 * I-set, 225 319 * I-set, 331 408 * I-set, 412 548 * V-set, 551 642 * Pkinase, 756 1021 * |
| ENSG00000112655 | ENSP00000326029 | 1014 | CCK4_Mm_TK_CCK4 | I-set, 32 121 * I-set, 128 217 * I-set, 225 319 * I-set, 331 408 * I-set, 412 498 * I-set, 502 588 * Pkinase, 740 1005 * |
|  |  |  |  |  |
| ENSG00000115085 | ENSP00000264972 | 619 | ZAP70_Mm_TK_Syk | SH2, 10 87 * SH2, 163 239 * Pkinase, 338 593 * |
| ENSG00000115085 | ENSP00000374169 | 312 | ZAP70_Mm_TK_Syk | Pkinase, 31 286 * |
|  |  |  |  |  |
| ENSG00000120899 | ENSP00000332816 | 1009 | PYK2_Mm_TK_FAK | FERM_M, 143 265 * Pkinase, 425 679 * Focal_AT, 870 1008 * |
| ENSG00000120899 | ENSP00000342242 | 967 | PYK2_Mm_TK_FAK | FERM_M, 143 265 * Pkinase, 425 679 * Focal_AT, 828 966 * |
| ENSG00000120899 | ENSP00000380634 | 596 | PYK2_Mm_TK_FAK | Pkinase, 171 425 * |
|  |  |  |  |  |
| ENSG00000123612 | ENSP00000335139 | 336 | ALK7_Mm_TKL_STKR | Activin_recp, 26 100 * Pkinase, 38 325 * |
| ENSG00000123612 | ENSP00000335178 | 413 | ALK7_Mm_TKL_STKR | Activin_recp, 26 100 * Pkinase, 115 402 * |
| ENSG00000123612 | ENSP00000387168 | 443 | ALK7_Mm_TKL_STKR | TGF_beta_GS, 115 143 * Pkinase, 145 432 * |
|  |  |  |  |  |
| ENSG00000128881 | ENSP00000263802 | 1649 | TTBK2_Mm_CK1_TTBK | Pkinase, 13 277 * Filament, 342 591 * |
| ENSG00000128881 | ENSP00000267890 | 1244 | TTBK2_Mm_CK1_TTBK | Pkinase, 21 279 * |
| ENSG00000128881 | ENSP00000382403 | 1174 | TTBK2_Mm_CK1_TTBK | Pkinase, 1 209 * |
|  |  |  |  |  |
| ENSG00000137764 | ENSP00000178640 | 448 | MAP2K5_Mm_STE_STE7 | PB1, 18 97 * Pkinase, 166 419 * |
| ENSG00000137764 | ENSP00000342101 | 258 | MAP2K5_Mm_STE_STE7 | Pkinase, 4 229 * |
| ENSG00000137764 | ENSP00000346493 | 442 | MAP2K5_Hs_STE_STE7 | PB1, 18 97 * Pkinase, 166 409 * |
| ENSG00000137764 | ENSP00000378859 | 438 | MAP2K5_Hs_STE_STE7 | PB1, 18 97 * Pkinase, 166 409 * |
|  |  |  |  |  |
| ENSG00000145349 | ENSP00000339740 | 499 | CaMK2g_Hs_CAMK_CAMK2 | Pkinase, 14 272 * CaMKII_AD, 346 473 * |
| ENSG00000145349 | ENSP00000369096 | 344 | CaMK2g_Hs_CAMK_CAMK2 | Pkinase, 14 272 * |
| ENSG00000145349 | ENSP00000378032 | 478 | CaMK2g_Hs_CAMK_CAMK2 | Pkinase, 14 272 * CaMKII_AD, 346 473 * |
| ENSG00000145349 | ENSP00000378034 | 489 | CaMK2g_Hs_CAMK_CAMK2 | Pkinase, 14 272 * CaMKII_AD, 357 484 * |
|  |  |  |  |  |
| ENSG00000147044 | ENSP00000354641 | 909 | CASK_Hs_CAMK_CASK | Pkinase, 12 276 * L27, 346 401 * L27, 405 458 * PDZ, 490 568 * SH3, 603 668 * Guanylate_kin, 758 862 * |
| ENSG00000147044 | ENSP00000367396 | 590 | CASK_Hs_CAMK_CASK | Pkinase, 12 276 * L27, 346 401 * L27, 405 458 * PDZ, 490 568 * |
| ENSG00000147044 | ENSP00000367400 | 909 | CASK_Hs_CAMK_CASK | Pkinase, 12 276 * L27, 346 401 * L27, 405 458 * PDZ, 490 568 * SH3, 603 668 * Guanylate_kin, 758 862 * |
| ENSG00000147044 | ENSP00000367405 | 926 | CASK_Hs_CAMK_CASK | Pkinase, 12 276 * L27, 346 401 * L27, 405 458 * PDZ, 490 568 * SH3, 615 680 * Guanylate_kin, 775 879 * |
| ENSG00000147044 | ENSP00000367408 | 921 | CASK_Hs_CAMK_CASK | Pkinase, 12 276 * L27, 346 401 * L27, 405 458 * PDZ, 490 568 * SH3, 615 680 * Guanylate_kin, 770 874 * |
| ENSG00000147044 | ENSP00000367410 | 859 | CASK_Hs_CAMK_CASK | Pkinase, 1 209 * L27, 279 334 * L27, 338 391 * PDZ, 423 501 * SH3, 548 613 * Guanylate_kin, 708 812 * |
| ENSG00000147044 | ENSP00000367421 | 897 | CASK_Hs_CAMK_CASK | Pkinase, 12 276 * L27, 340 395 * L27, 399 452 * PDZ, 484 562 * SH3, 586 651 * Guanylate_kin, 746 850 * |
|  |  |  |  |  |
| ENSG00000152332 | ENSP00000282169 | 419 | KIS_Mm_Other_KIS | Pkinase, 23 304 * RRM_1, 345 400 * |
| ENSG00000152332 | ENSP00000375995 | 344 | KIS_Mm_Other_KIS | Pkinase, 23 304 * |
|  |  |  |  |  |
| ENSG00000153208 | ENSP00000295408 | 999 | MER_Mm_TK_Axl | V-set, 64 194 * ig, 108 177 * ig, 211 264 * fn3, 284 368 * fn3, 383 473 * Pkinase, 587 854 * |
| ENSG00000153208 | ENSP00000376929 | 556 | MER_Mm_TK_Axl | Pkinase, 229 485 * |
| ENSG00000153208 | ENSP00000387277 | 823 | MER_Mm_TK_Axl | fn3, 108 192 * fn3, 207 297 * Pkinase, 411 678 * |
|  |  |  |  |  |
| ENSG00000160145 | ENSP00000291478 | 1289 | Trad_Hs_CAMK_Trio | RhoGEF, 236 406 * PH, 420 529 * V-set, 773 869 * fn3, 872 957 * Pkinase, 987 1241 * |
| ENSG00000160145 | ENSP00000346122 | 1257 | Trad_Hs_CAMK_Trio | RhoGEF, 204 374 * PH, 388 497 * V-set, 741 837 * fn3, 840 925 * Pkinase, 955 1209 * |
| ENSG00000160145 | ENSP00000353109 | 2986 | Trad_Hs_CAMK_Trio | Spectrin, 188 308 * Spectrin, 310 416 * Spectrin, 536 642 * Spectrin, 890 1004 * Spectrin, 1130 1236 * RhoGEF, 1285 1455 * PH, 1475 1580 * SH3, 1649 1709 * RhoGEF, 1933 2103 * PH, 2117 2226 * SH3, 2325 2384 * V-set, 2470 2566 * fn3, 2569 2654 * Pkinase, 2684 2938 * |
|  |  |  |  |  |
| ENSG00000162302 | ENSP00000294261 | 524 | MSK2_Mm_AGC_RSK | Pkinase, 33 301 * Pkinase_C, 321 365 * |
| ENSG00000162302 | ENSP00000333896 | 772 | MSK2_Mm_AGC_RSK | Pkinase, 33 301 * Pkinase_C, 321 365 * Pkinase, 411 674 * |
|  |  |  |  |  |
| ENSG00000163491 | ENSP00000350059 | 404 | 6348_Tt_Other_NEK | Pkinase, 3 180 * |
| ENSG00000163491 | ENSP00000379867 | 717 | NEK10_Sp_Other_NEK | Arm, 198 238 * Arm, 239 279 * Arm, 280 320 * Pkinase, 519 715 * |
| ENSG00000163491 | ENSP00000379876 | 559 | 6348_Tt_Other_NEK | Pkinase, 3 180 * |
|  |  |  |  |  |
| ENSG00000166851 | ENSP00000300093 | 603 | PLK1_Hs_Other_PLK | Pkinase, 53 305 * POLO_box, 417 480 * POLO_box, 515 584 * |
| ENSG00000166851 | ENSP00000331713 | 247 | PLK1_Hs_Other_PLK | Pkinase, 53 247 * |
|  |  |  |  |  |
| ENSG00000169071 | ENSP00000364860 | 943 | ROR2_Mm_TK_Ror | V-set, 34 153 * I-set, 62 152 * ig, 76 137 * Fz, 168 301 * Kringle, 316 394 * Pkinase, 473 746 * |
| ENSG00000169071 | ENSP00000364862 | 704 | ROR2_Mm_TK_Ror | Fz, 28 161 * Kringle, 176 254 * Pkinase, 333 606* |
| ENSG00000169071 | ENSP00000364867 | 704 | ROR2_Mm_TK_Ror | Fz, 28 161 * Kringle, 176 254 * Pkinase, 333 606* |
|  |  |  |  |  |
| ENSG00000169398 | ENSP00000341189 | 1052 | FAK_Mm_TK_FAK | FERM_M, 139 258 * Pkinase, 422 676 * Focal_AT, 914 1052 * |
| ENSG00000169398 | ENSP00000342839 | 553 | FAK_Mm_TK_FAK | Pkinase, 240 522 * |
| ENSG00000169398 | ENSP00000346424 | 877 | FAK_Mm_TK_FAK | Pkinase, 240 522 * Focal_AT, 739 877 * |
| ENSG00000169398 | ENSP00000378640 | 894 | FAK_Mm_TK_FAK | Pkinase, 236 518 * Focal_AT, 756 894 * |
| ENSG00000169398 | ENSP00000378644 | 1006 | FAK_Mm_TK_FAK | FERM_M, 139 258 * Pkinase, 422 676 * Focal_AT, 868 1006 * |
| ENSG00000169398 | ENSP00000378647 | 1007 | FAK_Mm_TK_FAK | FERM_M, 49 168 * Pkinase, 374 628 * Focal_AT, 869 1007 * |
| ENSG00000169398 | ENSP00000378649 | 1073 | FAK_Mm_TK_FAK | FERM_M, 160 279 * Pkinase, 443 697 * Focal_AT, 935 1073 * |
|  |  |  |  |  |
| ENSG00000177189 | ENSP00000368865 | 227 | RSK1_Mm_AGC_RSK | Pkinase, 39 227 * |
| ENSG00000177189 | ENSP00000368884 | 740 | RSK1_Mm_AGC_RSK | Pkinase, 68 327 * Pkinase_C, 347 391 * Pkinase, 422 679 * |
|  |  |  |  |  |
| ENSG00000182511 | ENSP00000331504 | 822 | FES_Mm_TK_Fer | FCH, 1 94 * SH2, 460 530 * Pkinase, 561 814 * |
| ENSG00000182511 | ENSP00000377837 | 764 | FES_Mm_TK_Fer | FCH, 1 98 * SH2, 402 472 * Pkinase, 503 756 * |
| ENSG00000182511 | ENSP00000377839 | 681 | FES_Mm_TK_Fer | FCH, 1 98 * Pkinase, 433 673 * |
| ENSG00000182511 | ENSP00000377841 | 752 | FES_Mm_TK_Fer | FCH, 1 94 * Pkinase, 491 744 * |
|  |  |  |  |  |
| ENSG00000182541 | ENSP00000330470 | 617 | LIMK2_Mm_TKL_LISK | LIM, 6 47 * LIM, 51 108 * PDZ, 131 215 * Kdo, 298 490 * Pkinase, 310 580 * |
| ENSG00000182541 | ENSP00000332687 | 638 | LIMK2_Mm_TKL_LISK | LIM, 12 68 * LIM, 72 129 * PDZ, 152 236 * Kdo, 319 511 * Pkinase, 331 601 * |
| ENSG00000182541 | ENSP00000339916 | 686 | LIMK2_Mm_TKL_LISK | LIM, 6 47 * LIM, 51 108 * PDZ, 131 215 * Kdo, 298 490 * Pkinase, 310 608 * PP1_inhibitor, 558 686 * |
| ENSG00000182541 | ENSP00000384602 | 629 | LIMK2_Mm_TKL_LISK | LIM, 2 51 * PDZ, 74 158 * Kdo, 241 433 * Pkinase, 253 551 * PP1_inhibitor, 501 629 * |
|  |  |  |  |  |
| ENSG00000183765 | ENSP00000372021 | 452 | CHK2_Mm_CAMK_RAD53 | Pkinase, 129 395 * |
| ENSG00000183765 | ENSP00000372023 | 586 | CHK2_Mm_CAMK_RAD53 | FHA, 156 235 * Pkinase, 263 529 * |
| ENSG00000183765 | ENSP00000386087 | 543 | CHK2_Mm_CAMK_RAD53 | FHA, 113 192 * Pkinase, 220 486 * |
|  |  |  |  |  |
| ENSG00000184304 | ENSP00000374634 | 875 | PKD3_Hs_CAMK_PKD | C1_1, 147 199 * C1_3, 158 189 * C1_1, 271 323 * C1_3, 282 313 * PH, 423 541 * Pkinase, 583 802 * |
| ENSG00000184304 | ENSP00000374635 | 912 | PKD3_Hs_CAMK_PKD | C1_1, 147 199 * C1_3, 158 189 * C1_1, 271 323 * C1_3, 282 313 * PH, 423 541 * Pkinase, 583 839 * |
| ENSG00000184304 | ENSP00000379907 | 849 | PKD3_Hs_CAMK_PKD | C1_1, 147 199 * C1_1, 271 320 * PH, 397 515 * Pkinase, 557 776 * |
|  |  |  |  |  |
| ENSG00000185532 | ENSP00000363086 | 283 | PKG1_Hs_AGC_PKG | Pkinase, 1 231 * |
| ENSG00000185532 | ENSP00000363092 | 686 | PKG1_Hs_AGC_PKG | cNMP_binding, 136 221 * cNMP_binding, 254 345 * Pkinase, 375 634 * |
| ENSG00000185532 | ENSP00000384200 | 671 | PKG1_Hs_AGC_PKG | cNMP_binding, 121 206 * cNMP_binding, 239 330 * Pkinase, 360 619 * |
|  |  |  |  |  |
| ENSG00000198400 | ENSP00000351486 | 796 | TRKA_Mm_TK_Trk | LRR_1, 67 90 * LRR_1, 92 114 * LRR_1, 116 138 * LRR_1, 139 162 * I-set, 194 282 * Pkinase, 510 781 * |
| ENSG00000198400 | ENSP00000357179 | 790 | TRKA_Mm_TK_Trk | LRR_1, 67 90 * LRR_1, 92 114 * LRR_1, 116 138 * LRR_1, 139 162 * I-set, 194 282 * Pkinase, 504 775 * |
| ENSG00000198400 | ENSP00000357180 | 760 | TRKA_Mm_TK_Trk | I-set, 164 252 * Pkinase, 474 745 * |
| ENSG00000198400 | ENSP00000376120 | 760 | TRKA_Mm_TK_Trk | I-set, 164 252 * Pkinase, 474 745 * |
